# Sex-specific correlation of IGFBP-2 and IGFBP-3 with vitamin D status in adults with obesity: a cross-sectional serum proteomics study

**DOI:** 10.1038/s41387-018-0063-8

**Published:** 2018-10-04

**Authors:** Nasser M. Al-Daghri, Antigoni Manousopoulou, Majed S. Alokail, Sobhy Yakout, Amal Alenad, Diana J. Garay-Baquero, Miltiadis Fotopoulos, Jie Teng, Omar Al-Attas, Yousef Al-Saleh, Shaun Sabico, George P. Chrousos, Spiros D. Garbis

**Affiliations:** 1 Biochemistry Department, College of Science, Biomarkers Research Program, King Saud University, Riyadh, Saudi Arabia; 2 Biochemistry Department, Prince Mutaib Chair for Biomarkers of Osteoporosis, King Saud University, Riyadh, Saudi Arabia; 3 Centre for Proteomic Research, Institute for Life Sciences, University of Southampton, Southampton, UK; 4 School of Pharmacy, Tianjin Medical University, Tianjin, China; 5 1st Department of Pediatrics, University of Athens, Athens, Greece; 6 Cancer Sciences Unit, Faculty of Medicine, University of Southampton, Southampton, UK; 7 Present Address: Proteome Exploration Laboratory, Beckman Institute, Division of Biology and Biological Engineering, California Institute of Technology, Pasadena, CA 91125 USA

## Abstract

**Objective:**

Subjects with low vitamin D levels are at risk of cardiometabolic disease. The aim of this study was to identify novel serological markers linking vitamin D status with cardiometabolic profile in non-diabetic adults with obesity.

**Methods:**

For the discovery phase, we used quantitative serum proteomics in sex-matched, age-matched and BMI-matched subjects with obesity [BMI: 25–35 kg/m^2^] and low [25(OH)D < 50 nmol/L] vs. high vitamin D status [25(OH)D > 50 nmol/L] (*n* = 16). For the validation phase, we performed ELISA in a larger cohort with similar characteristics (*n* = 179).

**Results:**

We identified 423 and 549 differentially expressed proteins in the high vs. low vitamin D groups of the male and female cohorts, respectively. The *small molecule biochemistry* protein networks and the *glycolysis|gluconeogenesis* pathway were significantly enriched in the DEPs of both sexes. As surrogate markers to these processes, the insulin-like growth factor binding protein -2  (IGFBP-2) was upregulated in males, whereas   IGFBP-3 was upregulated in females from the high Vitamin D status. This sex-specific trend  was confirmed using Luminex ELISA to an independent but clinically analogous cohort of males (*n* = 84, *p* *=* 0.002) and females (*n* = 95, *p* *=* 0.03).

**Conclusions:**

The high Vitamin D status correlated with the serological upregulation of IGFBP-2 in males and IGFBP-3 in females with obesity and may constitute surrogate markers of risk reduction of cardiometabolic disease.

## Introduction

Vitamin D is an ancient hormone, originally produced by archaebacteria, phytoplankton and zooplankton dating back to over 500 million years^1^. Almost all mammalian cell types express the vitamin D receptor, suggesting that vitamin D may exhibit a pleiotropic effect in addition to its well-established role in calcium and phosphorus homoeostasis^2^. A clinically relevant marker used to measure vitamin D status is circulating levels of 25(OH)D^3^. Although there is significant controversy in this field, vitamin D insufficiency is defined by the Institute of Medicine (IOM) as serum 25(OH)D levels lower than 50 nmol/L^4^. Notably, the Endocrine Society suggested that circulating 25(OH)D should be maintained at higher concentrations (75 to 80 nmol/L) for extra-skeletal health benefits^5^ with no known toxicity at this level^6,7^. Across countries in all continents, the mean serum concentration of 25(OH)D is around 50 nmol/L, suggesting that approximately 50% of these populations have vitamin D insufficiency^8,9^.

Emerging evidence suggests  the extra-skeletal, pluripotent effects of vitamin D in reducing the risk of adverse cardiometabolic outcomes. A meta-analysis, including 65,000 prospectively monitored participants, showed that the group with the lowest compared to the highest serum 25(OH)D levels had a relative risk of 1.5 (1.3 to 1.8) for total incidents of cardiovascular disease and 1.6 (1.3 to 2.1) for stroke^10^. Another meta-analysis of approximately 500,000 participants found an inverse association with all-cause mortality for circulating 25(OH)D levels up to 75 nmol/L^11^. An additional dose-response meta-analysis from 34 studies involving 180,667 participants demonstrated that serum 25(OH)D levels were inversely correlated with total number of CVD events and CVD mortality^12^. Decreased 25(OH)D levels may affect cardiovascular risk either directly, for example by increasing blood pressure through the renin-angiotensin system, or indirectly, by influencing inflammation, myocardial function, vascular calcification and parathyroid hormone levels^13^.

An additional potential extra-skeletal effect of vitamin D is its chemopreventive effects against type II diabetes mellitus (T2DM). The meta-analysis of well-powered clinical studies have shown that subjects with low 25(OH)D levels have an increased risk of developing T2DM compared to those with a normal vitamin D status^14^. Results from a population study in Victoria, Australia showed an inverse association between vitamin D status and risk factors of T2DM, including fasting plasma glucose and HbA1c levels^15^. A recent systematic review and meta-analysis that included almost 30,000 subjects, found that lower 25(OH)D levels were significantly associated with an increased risk of developing diabetes among older adults^16^. Low vitamin D status may lead to insulin resistance by impairing insulin secretion and compromising pancreatic beta-cell function, all hallmark features of T2DM^17,18^. Recent findings suggest that supplementation with vitamin D could positively affect insulin secretion and glucose homoeostasis^19,20^. An additional recently published Mendelian randomisation study in European and Chinese adults provided first ever evidence for a causal effect of higher 25(OH)D serum levels for the prevention of T2DM^21^. As a corollary to these observations, the chemoprevention of T2D prior to its clinical diagnosis in individuals with obesity by means of Vitamin D status correction may also reduce the risk of cardiometabolic disease. However, surrogate protein markers that can easily be measured in serum to gauge such a risk reduction due to Vitamin D status improvement are currently lacking. 

**Fig. 1:**
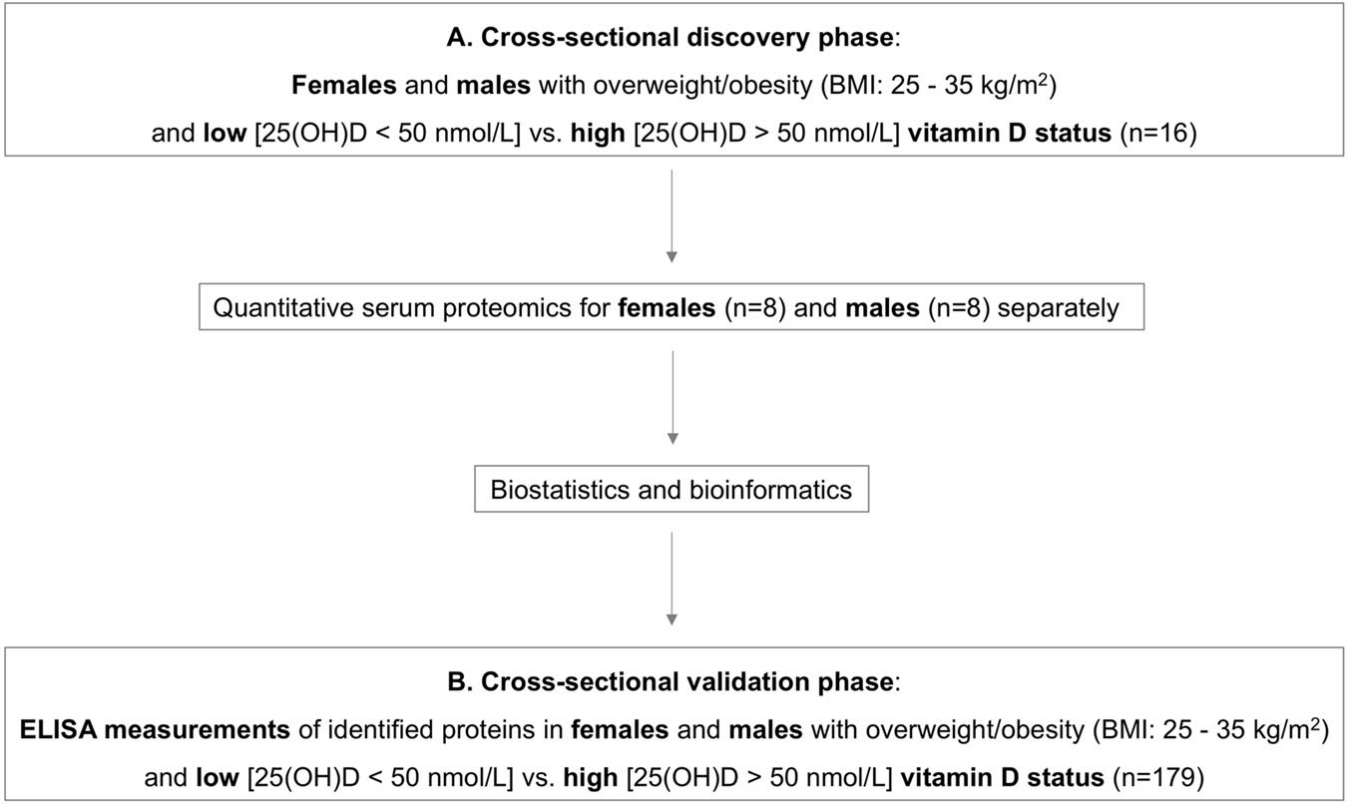
Study design: a cross-sectional quantitative serum proteomics discovery phase with an ELISA validation phase for the identification of novel serological markers of vitamin D status linked to cardiometabolic outcomes among adults with overweight/obesity

The aim of the present study was to perform quantitative serum proteomics in a cross-sectional cohort of non-diabetic adults with obesity and low vs. high vitamin D status in order to identify novel serological markers linking vitamin D status to cardiometabolic disease risk. The relevant differentially expressed proteins chosen as candidate markers were further verified against a larger cross-sectional cohort with the same inclusion/exclusion criteria as for the discovery phase. An overview of the study design is presented in Fig. 1.

## Materials and methods

### Discovery phase: serum proteomics in cross-sectional samples

The study received ethics approval by the King Saud University Ethics Committee. Reporting of the cross-sectional study conforms to the STROBE statement and the broader EQUATOR guidelines^22^. For the cross-sectional serum proteomics study, participants were randomly selected from an existing cohort, the Riyadh cohort 2^23^. Written informed consent was obtained from all participants. Adults with overweight/obesity (BMI between 25 and 35 kg/m^2^) and with normal fasting plasma glucose levels (3.9 to 5.5 mmol/L) and serum 25(OH)D < 50nmol/L or >50 nmol/L were included in the study. Subjects pregnant or breastfeeding, with morbid obesity (BMI > 35 kg/m^2^), diagnosed with type 1 or type 2 diabetes mellitus, or with non-alcoholic fatty liver disease were excluded from the study. An abdominal ultrasound was performed to exclude non-alcoholic fatty liver disease among the study participants. Anthropometry and morning blood withdrawal was performed after overnight fasting. Blood collection for all participants took place during the winter months (December to February). The discovery cohort comprised 16 participants (four males with serum 25(OH)D < 50nmol/L; four males with serum 25(OH)D > 80 nmol/L; four females with serum 25(OH)D < 50nmol/L; four females with serum 25(OH)D > 80 nmol/L). Participants were asked about their sun exposure habits using a questionnaire as reported before^24^. Total energy and micronutrient (vitamin D, calcium and EPA/DHA) intake was estimated using food frequency questionnaires as reported before^24^. Physical activity levels of the participants were assessed using the WHO Global Physical Activity Questionnaire, as reported previously^24^.

### Serum procurement and proteomic analysis

The procurement and handling of sera was in accordance with the recommendations of the Standard Operating Procedure Integration Working Group (SOPIWG) as adopted by the author’s method^25^. Two eight-plex serum proteomics experiments were performed for male and female subjects separately, as we have shown previously that vitamin D has sex-specific non-skeletal cardiovascular effects^24^. The serum specimens were freshly thawed and vortexed for 2 min. For each participant, 100 µL of unprocessed serum were immediately mixed with 400 µL 6 M Guanidine Hydrochloride and subjected to global quantitative serum proteomic analysis using our previously published method^24,25^. In summary, high-performance Size Exclusion Chromatography using three serially connected Waters KW-804 columns at 0.75 ml/min flow rate and 30 °C was used to separate the proteins based on their molecular weight differences. The separated low-molecular weight protein segments (molecular weight cutoff 3 kDa) were dialysis purified and lyophilised to dryness at 4 °C. One-hundred μg of protein from each sample was subjected to trypsin proteolysis and the peptides were chemically labelled using the eight-plex isobaric Tag for Relative and Absolute Quantitation (iTRAQ) reagents, pooled, and offline fractionated with C_4_ reverse phase chromatography at pH 8.0 (2.1 mm X 150 mm, 3 μm particle, 120 Å pore, Kromasil, Germany). As a multiplex proteomics workflow, samples were analysed under the same exact experimental conditions. Each fraction was analysed using ultra-high performance C_18_ nano-liquid chromatography at pH 3.5 (75μm ID x 50 cm, 2 μm particle, 100 Å pore, Hypersil, USA) hyphenated with high-resolution tandem mass spectrometry using the FT-Orbitrap Elite platform.

Unprocessed raw files were submitted to Proteome Discoverer 1.4 for target decoy search against the UniProtKB Homo Sapiens database (release date 10-Jan-2015) using SequestHT. Only reporter ion ratios from unique peptides were considered for the quantitation of the respective protein. Median normalisation and log_2_ transformation was performed for the reporter ion quantification ratios. A protein was considered differentially expressed between the high vs. low vitamin D status when its one-sample, two-tailed, *T*-test *p*-value was ≤0.05 and the mean iTRAQ log_2_ratio of high vs. low vitamin D status was higher than ±0.3. All mass spectrometry proteomics data have been deposited to the ProteomeXchange Consortium via the PRIDE partner repository with the dataset identifier PXD009606.

### Principal component analysis and bioinformatics with biostatistics informed selection of candidate verification markers

Principal component analysis using the reporter ion ratios of the all analysed proteins in the male and female cohorts, respectively was performed using ClustVis (https://biit.cs.ut.ee/clustvis/). DAVID (https://david.ncifcrf.gov/) and Ingenuity Pathway Analysis (IPA) (Qiagen, Hilden, Germany) software tools were used to identify canonical pathways and protein networks significantly over-represented in the differentially expressed proteins between high and low vitamin D status groups of the male and female cohorts. Significance was set at *p*-value ≤0.05. Surrogate markers to the identified enriched pathways were further evaluated as candidate verification markers with bibliographic research. As an additional qualifier, retrospective statistical power analysis using the function *pwr.2p.test()* available within R (https://www.R-project.org/) was applied to the suitable candidate marker as reported^26^. The minimum statistical power threshold was set at 0.8, which factored in the *p*-value, variation, differential expression ratio, and the number of replicate biological observations for the chosen protein analysed from the discovery experiment.

### Verification phase: ELISA measurements in an independent cross-sectional cohort

ELISA measurements of selected proteins identified at the discovery phase were validated in a larger cross-sectional cohort of adults with obesity randomly selected from the Riyadh cohort 2^23^, using the same inclusion and exclusion criteria as described above for the discovery phase. In total, 179 adults were included, of which 84 males [*n* = 40 with 25(OH) < 50 nmol/L; *n* = 44 with 25(OH) > 50 nmol/L] and 95 females [*n* = 47 with 25(OH) < 50 nmol/L; *n* = 48 with 25(OH) > 50 nmol/L]. The size of the verification cohort was based on the logistic regression models requiring a minimum of 10 events per predictor variable^27^, which in our study included sex and 25(OH)D levels. All other clinical parameters listed in Table 2 were not considered, as they remained constant between the cohorts examined.

### Insulin-like growth factor binding protein IGFBP-2 and IGFBP-3 ELISA measurements

IGFBP-2 and IGFBP-3 were targeted for measurement using the commercially available Luminex ELISA kit (Catalog number: HIGFBMAG-53K; Millipore, Billerica, MA, USA) according to manufacturer’s instructions. The captured bead complexes were measured with the FLEXMAP 3D system (Luminex Corporation, Austin, TX). The raw data (mean fluorescence intensity) were collected and further processed for calculating protein concentration. The intra-assay and inter-assay coefficient of variation (CV) was <10 and <15%, respectively.

### Biochemical analyses

Fasting glucose levels were measured using an enzymatic assay (hexokinase coupled with glucose 6-phosphate dehydrogenase) with a chemical analyser (Konelab, Espoo, Finland). The inter-assay CV was 2.2%. Serum 25(OH)D levels were measured by a specific enzyme-linked immunosorbent assay (IDS, Tyne and Wear, UK). The inter- and intra-assay variability of this assay was 5.1 and 4.6%, respectively.

### Clinical data analysis

Clinical data were analysed using SPSS (Version 25). An unpaired, two-tailed Student *T*-test was applied to compare the clinical and lifestyle characteristics of the low vs. high vitamin D status groups of males and females in the discovery and validation cohorts. A Mann–Whitney *U* Test was used to compare IGFBP-2 and IGFBP-3 levels in low vs. high vitamin D status groups of males and females in the validation cohort. Parameters are presented as mean ± standard deviation or median (25th to 75th percentile). A *p*-value less than 0.05 was considered significant.

## Results

The anthropometric, lifestyle and clinical characteristics of the participants in the discovery phase are presented in Table 1. The low and high vitamin D status groups of the male and female participants were similar with regards to age, BMI, fasting glucose, sun exposure, physical activity and total energy, DHA/EPA and calcium intake. The two groups had significantly different serum 25(OH)D levels (*p* < 0.0001) as per the inclusion criteria and vitamin D intake (*p* < 0.0001).

**Table 1:** Clinical characteristics of discovery cohort. Numerical values in bold font applied to statistically significant differences

Parameters	Males		*P*-value	Females		*P*-value
	Low 25 (OH)D	High 25 (OH)D		Low 25 (OH)D	High 25 (OH)D	
*N*	4	4		4	4	
Age (years)	42.0 ± 8.5	43.0 ± 8.3	0.80	39.8 ± 10.8	43.4 ± 9.7	0.68
BMI (kg/m^2^)	30.0 ± 4.0	31.2 ± 3.8	0.72	31.8 ± 2.5	32.7 ± 3.9	0.75
Systolic BP (mmHg)	130.1 ± 9.7	131.3 ± 10.0	0.90	125.7 ± 14.2	126.1 ± 15.7	0.97
Diastolic BP (mmHg)	82.9 ± 8.0	81.0 ± 8.5	0.75	80.3 ± 10.1	78.2 ± 10.5	0.81
Fasting glucose (mmol/l)	4.4 ± 0.8	4.5 ± 0.8	0.89	4.7 ± 0.7	4.6 ± 0.8	0.87
Triglycerides (mmol)	1.8 (1.5–2.5)	1.7 (1.2–2.3)	0.82	1.6 (1.2–2.0)	1.7 (1.5–2.3)	0.48
Total cholesterol (mmol/l)	5.6 ± 1.0	5.3 ± 1.6	0.80	5.3 ± 1.4	5.2 ± 1.4	0.93
HDL-cholesterol (mmol/l)	1.0 ± 0.3	1.2 ± 0.2	0.39	1.1 ± 0.3	1.3 ± 0.2	0.39
25(OH)D (nmol/l)	15.0 ± 4.5	105.0 ± 15.0	**<0.0001**	18.5 ± 5.0	100.0 ± 19.0	**<0.0001**
Sun exposure (hr/week)	3.1 ± 0.4	3.3 ± 0.5	0.62	3.0 ± 0.7	3.2 ± 0.5	0.71
Physical activity (MET-min/week)	500 ± 180	510 ± 200	0.95	480 ± 200	470 ± 220	0.95
Total energy intake (kcal/day)	2330 ± 350	2490 ± 460	0.64	2150 ± 270	2100 ± 350	0.85
DHA/EPA intake (mg/day)	340 ± 60	355 ± 60	0.77	325 ± 65	338 ± 79	0.83
Calcium intake (mg/day)	800 ± 260	820 ± 250	0.92	770 ± 210	775 ± 190	0.97
Vitamin D intake (iU/day)	300 ± 150	1500 ± 500	**<0.0001**	280 ± 160	1440 ± 380	**<0.0001**

*Note*: Clinical characteristics of the subjects in the cross-sectional discovery phase

Data are presented as mean ± SD or median (25th–75th percentile)

**Fig. 2:**
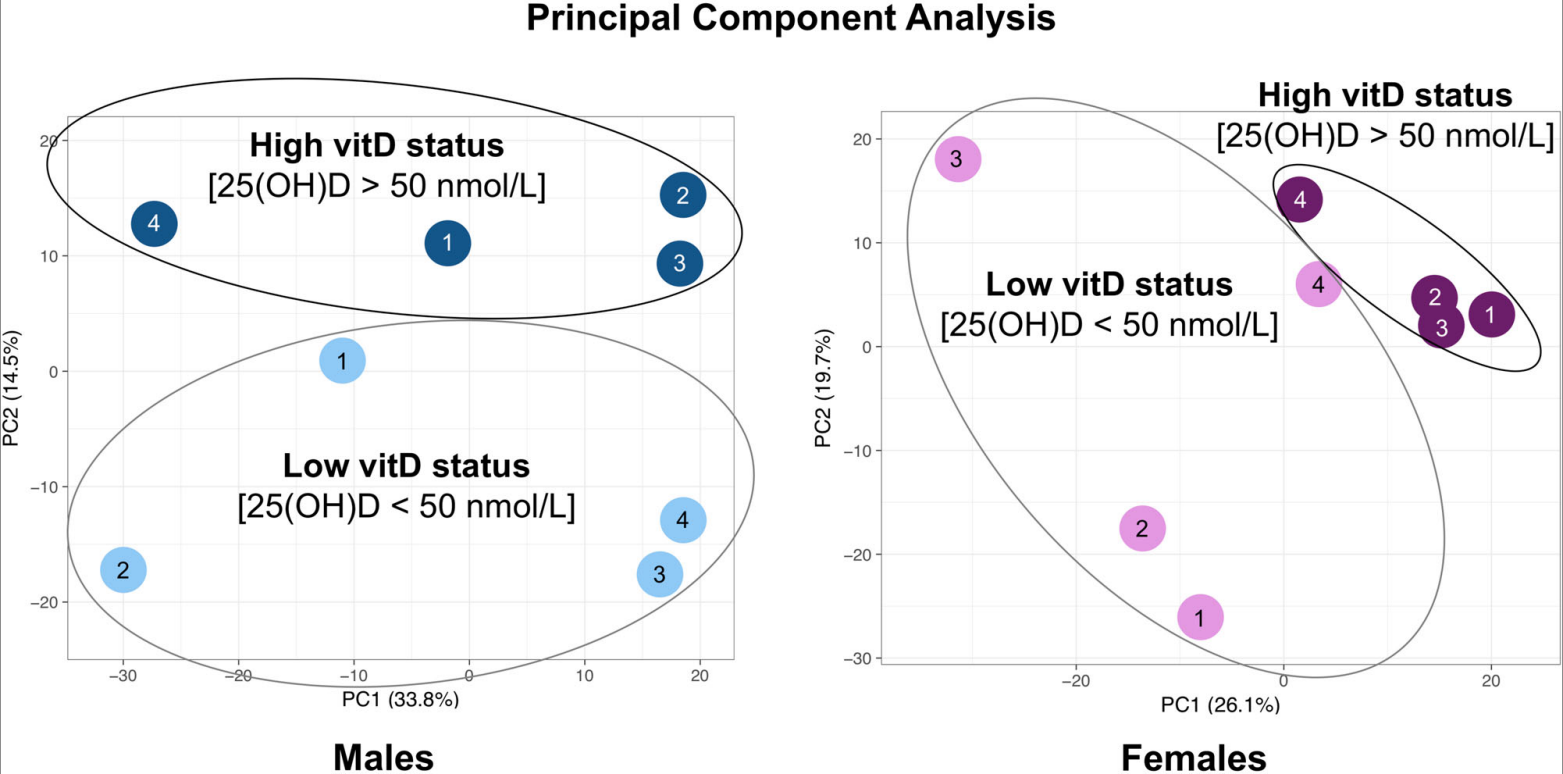
Principal component analysis using the iTRAQ log_2_ratio of all analysed proteins in high vs. low vitamin D status groups for male and female subjects of the cross-sectional study

**Fig. 3:**
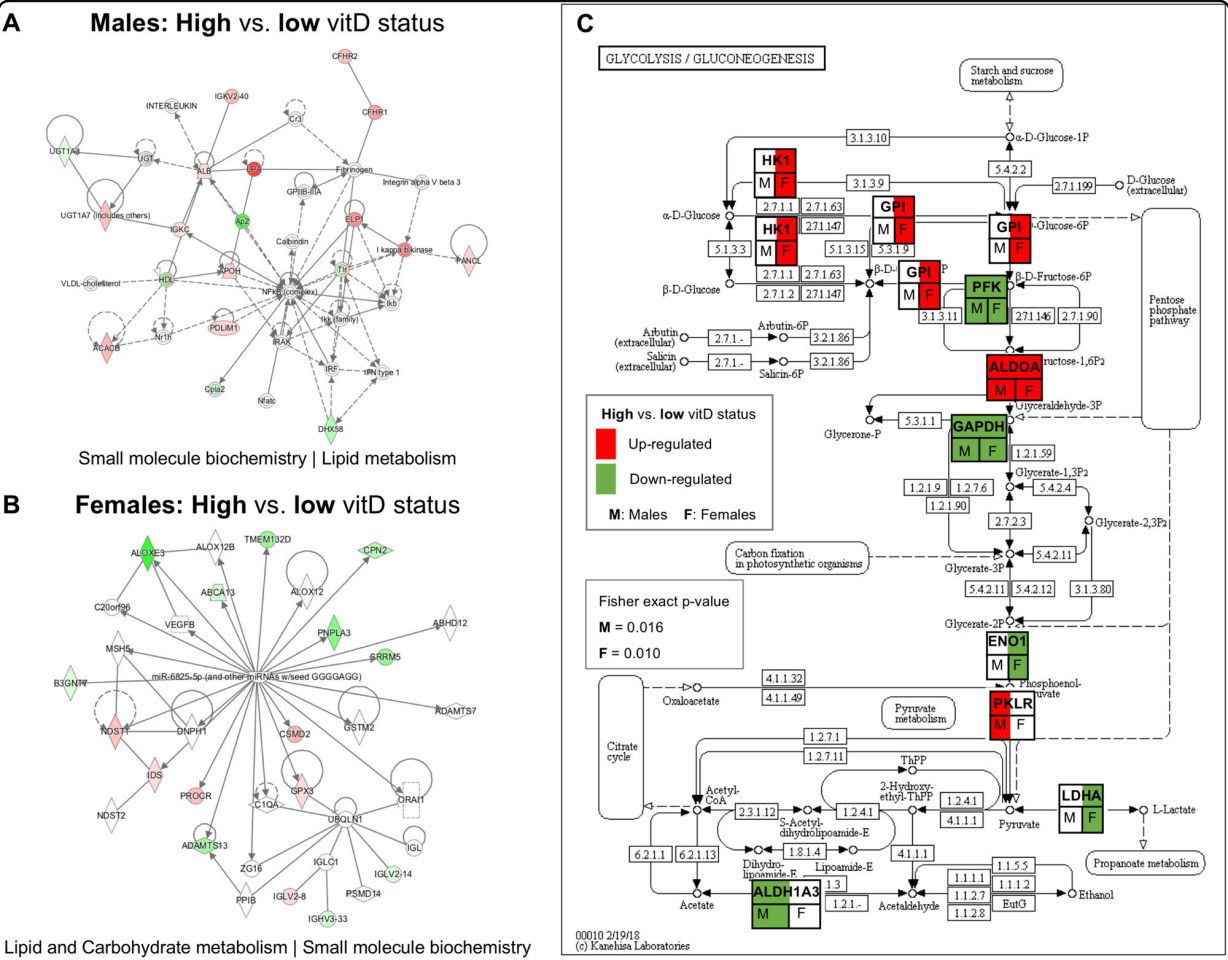
**a**, **b**. IPA analysis showed that protein networks related to *small molecule biochemistry* were enriched in both male and female cohorts (score = 17 and 19 for males and females, respectively). **c** KEGG canonical pathway analysis showed that *glycolysis|gluconeogenesis* was significantly enriched in the differentially expressed proteins (DEPs) of the male and female cohorts (Fisher exact *p* = 0.002 and 0.028, respectively for males and females). Protein name abbreviations: ALDH1A3 Aldehyde dehydrogenase family 1 member A3, ALDOA Fructose-biphosphate aldolase a, ENO1 Alpha-enolase, GAPDH Glyceraldehyde 3-phosphate dehydrogenase, GPI Glucose-6-phosphate isomerase, HK1 Hexokinase 1, LDHA L-lactate dehydrogenase A chain, PFK ATP dependent 6-phosphofructokinase, PKLR Pyruvate kinase

In the male and female cohorts of the cross-sectional study, 1297 and 1114 protein groups were profiled, respectively (peptide level FDR *p* < 0.05). Principal component analysis using the iTRAQ log_2_ratio of all analysed proteins in high vs. low vitamin D status groups for male and female subjects of the cross-sectional study is presented in Fig. 2. Of the quantitatively analysed serum proteins, 423 and 549 proteins were differentially expressed in the high vs. low vitamin D status conditions of the male and female cohorts, respectively (Supplementary Tables 1 and 2). IPA analysis showed that protein networks related to *small molecule biochemistry* were enriched in both male and female cohorts (score = 17 and 19 for males and females, respectively) (Fig. 3a, b). KEGG canonical pathway analysis showed that *glycolysis|gluconeogenesis* was significantly enriched in the differentially expressed proteins (DEPs) of the male and female cohorts (Fisher exact *p* = 0.002 and 0.028, respectively for males and females) (Fig. 3c). The secreted IGFBP2 and IGFBP3 proteins, as surrogate markers of glucose and fatty acid homeostasis through their engagement with the IGF/Insulin complex^55^, were found to be differentially expressed between low and high Vitamin D status of both male and female cohorts.

**Fig. 4:**
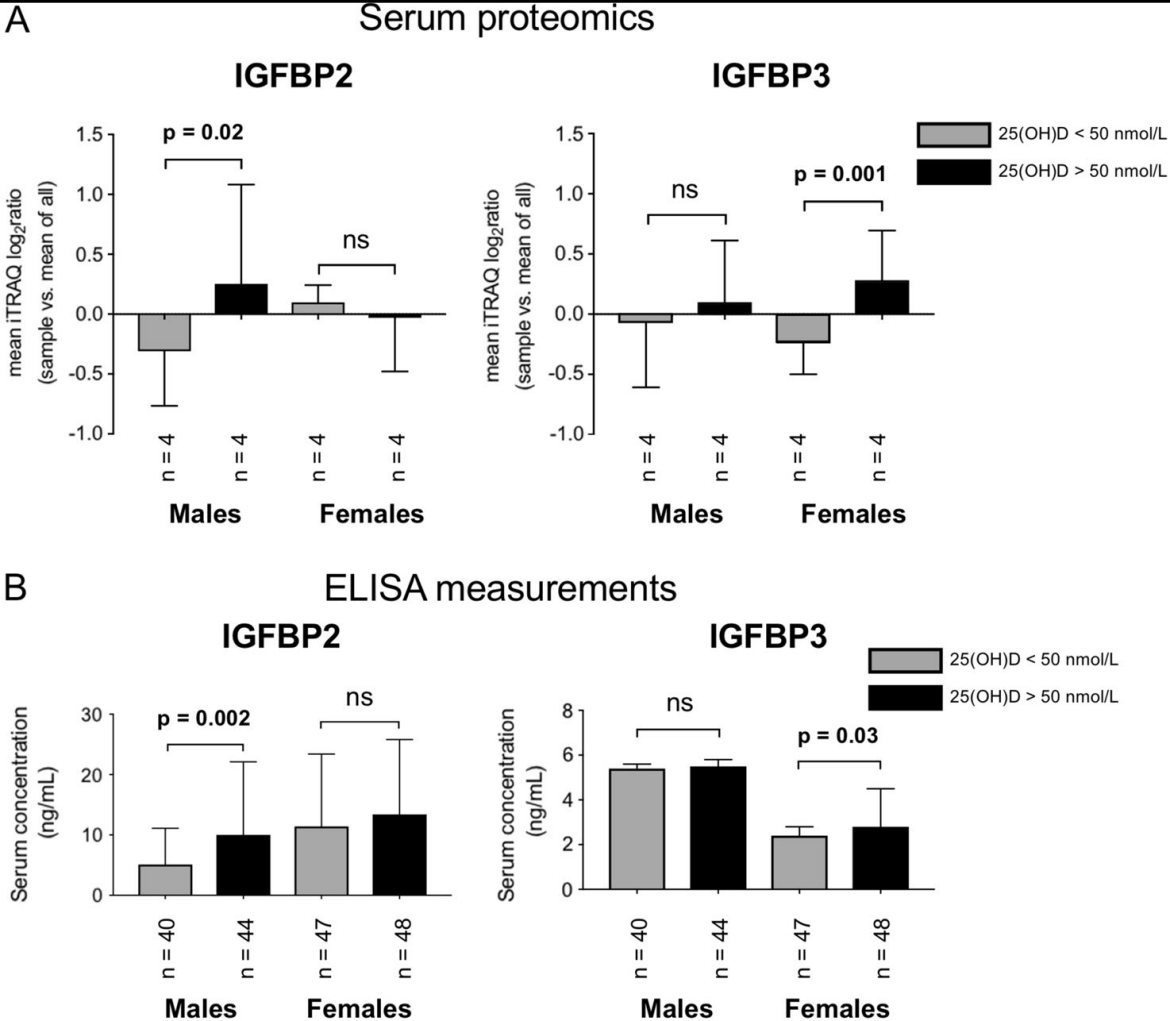
**a** Quantitative proteomic analysis of IGFBP-2 and IGFBP-3 in the male and female cross-sectional cohorts [Males: IGFBP-2 mean iTRAQ log_2_ratio in high vs. low vitamin D status (SD) = 0.6 (0.8), *p* = 0.02; Females: IGFBP-3 mean iTRAQ log_2_ratio in high vs. low vitamin D status (SD) = 0.5 (0.5), *p* = 0.001]. **b** ELISA measurements confirmed the sexually dimorphic correlation of IGFBP-2 and IGFBP-3 with vitamin D status among adults with obesity [Males: IGFBP-2 Low vitamin D status median (25th to 75th percentile) = 5.1 (2.9–11.1), High vitamin D status median (25th to 75th percentile) = 10.0 (5.6–22.1), *p* = 0.002; IGFBP-3 Low vitamin D status median (25th to 75th percentile) = 5.4 (3.4–5.6), High vitamin D status median (25th to 75th percentile) = 5.5 (3.9–5.8), *p* = 0.66] [Females: IGFBP-2 Low vitamin D status median (25th to 75th percentile) = 11.4 (6.6–23.4), High vitamin D status median (25th to 75th percentile) = 13.4 (5.7–25.8), *p* = 0.99; IGFBP-3 Low vitamin D status median (25th to 75th percentile) = 2.4 (1.6–2.8), High vitamin D status median (25th to 75th percentile) = 2.8 (2.7–4.5). *p* = 0.03]

In particular,  the discovery serum proteomics analysis showed that IGFBP-2 was over-expressed in men with high compared to low vitamin D status [Males: IGFBP-2 mean iTRAQ log_2_ratio in high vs. low vitamin D status (SD) = 0.6 (0.8), *p* = 0.02] whereas IGFBP-3 was expressed at higher levels in women with high compared to low vitamin D status [Females: IGFBP-3 mean iTRAQ log_2_ratio in high vs. low vitamin D status (SD) = 0.5 (0.5), *p* = 0.001] (Fig. 4a). This sex-specific correlation of vitamin D status with IGFBP-2 and IGFBP-3 was further examined using Luminex ELISA analysis against an independent cross-sectional cohort.

The clinical characteristics of the validation cohort are presented in Table 2. As for the discovery cohort, the low and high vitamin D status groups of the male and female participants were similar with regards to age, BMI, fasting glucose, sun exposure, physical activity and total energy, DHA/EPA and calcium intake. The two groups had significantly different vitamin D intake (*p* < 0.0001) and vitamin D status (*p* < 0.0001). ELISA measurements confirmed the sexually dimorphic correlation of IGFBP-2 and IGFBP-3 with vitamin D status among adults with obesity [Males: IGFBP-2 Low vitamin D status median (25th to 75th percentile) = 5.1 (2.9–11.1), High vitamin D status median (25th to 75th percentile) = 10.0 (5.6–22.1), *p* = 0.002; IGFBP-3 Low vitamin D status median (25th to 75th percentile) = 5.4 (3.4–5.6), High vitamin D status median (25th to 75th percentile) = 5.5 (3.9–5.8), *p* = 0.66] [Females: IGFBP-2 Low vitamin D status median (25th to 75th percentile) = 11.4 (6.6–23.4), High vitamin D status median (25th to 75th percentile) = 13.4 (5.7–25.8), *p* = 0.99; IGFBP-3 Low vitamin D status median (25th to 75th percentile) = 2.4 (1.6–2.8), High vitamin D status median (25th to 75th percentile) = 2.8 (2.7–4.5), *p* = 0.03] (Fig. 4b).

**Table 2:** Clinical characteristics of validation cohort. Numerical values in bold font applied to statistically significant differences

Parameters	Males		*P*-value	Females		*P*-value
	Low 25 (OH)D	High 25 (OH)D		Low 25 (OH)D	High 25 (OH)D	
*N*	40	44		47	48	
Age (years)	43.0 ± 8.7	43.8 ± 8.9	0.66	39.8 ± 10.8	43.4 ± 9.7	0.10
BMI (kg/m^2^)	30.6 ± 3.3	31.6 ± 3.5	0.20	31.8 ± 2.5	32.7 ± 3.9	0.20
Systolic BP (mmHg)	133.2 ± 10.2	131.9 ± 10.9	0.61	125.7 ± 14.2	126.1 ± 15.7	0.90
Diastolic BP (mmHg)	83.4 ± 7.8	80.8 ± 7.9	0.14	80.3 ± 10.1	78.2 ± 10.5	0.33
Fasting glucose (mmol/l)	4.8 ± 0.6	4.5 ± 0.7	0.60	4.5 ± 0.9	4.7 ± 0.6	0.76
Triglycerides (mmol)	1.7 (1.4–2.7)	1.9 (1.3–2.4)	0.61	1.5 (0.96–1.90)	1.7 (1.2–2.44)	0.13
Total Cholesterol (mmol/l)	5.5 ± 1.2	5.5 ± 1.4	0.96	5.1 ± 1.2	5.0 ± 1.0	0.72
HDL-Cholesterol (mmol/l)	1.0 ± 0.4	1.1 ± 0.4	0.66	1.1 ± 0.4	1.2 ± 0.4	0.29
25(OH)D (nmol/l)	31.5 ± 7.8	61.1 ± 9.8	**<0.0001**	26.6 ± 8.9	67.8 ± 13.8	**<0.0001**
IGFBP-2 (ng/ml)	5.1 (2.9–11.1)	10.0 (5.6–22.1)	**0.002**	11.4 (6.6–23.4)	13.4 (5.7–25.8)	0.99
IGFBP-3 (µg/ml)	5.4 (3.4–5.6)	5.5 (3.9–5.8)	0.66	2.4 (1.6–2.8)	2.8 (2.7–4.5)	**0.03**
Sun exposure (hr/week)	3.9 ± 0.5	3.5 ± 0.4	0.46	3.8 ± 0.6	3.9 ± 0.4	0.82
Physical activity (MET-min/week)	530 ± 190	520 ± 200	0.95	500 ± 210	490 ± 230	0.95
Total energy intake (kcal/day)	2350 ± 380	2400 ± 400	0.88	2180 ± 250	2200 ± 300	0.93
DHA/EPA intake (mg/day)	350 ± 80	365 ± 70	0.81	330 ± 70	340 ± 90	0.88
Calcium intake (mg/day)	810 ± 250	830 ± 250	0.52	750 ± 200	800 ± 180	0.76
Vitamin D intake (iU/day)	250 ± 100	1460 ± 450	**<0.0001**	230 ± 120	1500 ± 350	**<0.0001**

*Note*: Clinical characteristics of the subjects in the cross-sectional validation phase

## Discussion

Individuals with obesity and low Vitamin D status exhibit a substantially increased risk of cardiometabolic disease including its key component, T2D^2,10–21^. Such an increased risk is manifested well before the clinical diagnosis of cardiometabolic disease. Identifying novel protein markers at the minimally invasive blood serum level may improve our understanding and aggressive management of such at risk individuals. In this capacity, such serological protein markers may guage the effectiveness of Vitamin D intervention on reducing risk of cardiometabolic disease to individuals with obesity and low Vitamin D status. To address this need, the main aim of this study was to identify novel serum proteins as surrogate markers that may correlate Vitamin D status with cardiometabolic disease risk in non-diabetic adults with obesity using an agnostic quantitative serum proteomics approach (Fig. 1) based on the method developed by the authors^24,25^. The Principle Component Analysis of the entire serum proteome observed (Fig. 2) captured the anticipated sexual dimorphic protein expression, as previouly reported^24,25^. Futher bioinformatics interpretation (using Ingenuity and KEGG Pathway Analysis) of the differentially expressed proteins (*p<0.05*) between the high vs.low Vitamin D status conditions of the male and female cohorts (Suppl. Tables 1 and 2) resulted in the sexual dimorphic enrichment of the *small molecule biochemistry* protein networks (male and female enrichment scores of 17 and 19, respectively, Fig. 3 a and b) and the *glycolysis|gluconeogenesis* pathway (Fisher exact *p=0.02* for males and *p=0.028* for females, Fig. 3c). Interestingly, and consequent to the serum proteomic method used in this study, key participatory proteins observed in these enriched metabolic processes were of exosomal origin, as listed in the highly curated ExoCarta database (http://www.exocarta.org). Notable exosomal derived proteins found  to be differentially exprerssed between the male high vs. low Vitamin D status conditions in the *small molecule biochemistry* network were the PDZ and LIM domain 1 (*PDLIM1*); acetyl-CoA carboxylase beta (*ACACB*); and UDP glycosyltransferace 1 family, polypeptide A8 (*UGT1A8*) (Fig. 3a), whereas the differentially expressed proteins found in the female high vs. low Vitamin D status condition of the same network were carboxypeptidase N, polypeptide 2 (*CPN2*), protein C receptor, endothelial (*PROCR*); and ADAM metallopeptidase with thrombospondin type 1 motif, 13 (*ADAMTS13*) (Fig. 3b). For the *glycolysis|gluconeogenesis* pathway, key exosomal derived proteins differentially expressed in the high vs. low Vitamin D status included aldolase A, fructose-bisphosphate (*ALDOA*) found to be upregulated in both sexes; pyruvate kinase liver and red blood cell (*PKLR*) found upregulated in males; enolase 1, alpha (*ENO1*) found downregulated in females; and hexokinase 1 (*HK1*) found upregulated in females (Fig. 3c). An overarching regulatory process controlling the above described sugar and fatty acid metabolic traits is the growth hormone--insulin-like growth factor axis^28,29^. More precisely, the growth hormone—insulin-like growth factor axis is an evolutionary conserved system that controls somatic growth and metabolism^28^. Growth hormone (GH), a peptide hormone secreted by the anterior pituitary gland, is a stress hormone that counteracts the action of insulin and directly increases the concentration of glucose in the blood^29^. GH administration has been shown to increase gluconeogenesis and glycogenolysis from the kidney and liver^30,31^. Along these lines, patients with acromegaly exhibit increased gluconeogenic activity in the liver^30^ and are at risk of developing type 2 diabetes^32^. Furthermore, studies have found that GH suppresses glucose uptake by the adipose tissue, through the down-regulation of glucose transporter substrates on the plasma membrane of adipocytes^33^.

However, the effects of GH on glycaemic control are complex, since GH stimulates the production of insulin-like growth factor I (IGF1). Thus, GH deficiency is paradoxically associated with insulin resistance and abdominal obesity, a phenomenon possibly attributed to decreased IGF1 activity^34^. IGF1, a hormone primarily produced by the liver, has growth-promoting properties and insulin-like effects that are exerted through binding with the IGF1 receptor and insulin receptor^34,35^. Insulin-like growth factor II (IGF2) is a hormone closely related to IGF1, that also exerts growth-regulating and insulin-like activities.

The IGF1 and IGF2 components are carried in the systemic circulation by the soluble insulin-like growth factor binding proteins (IGFBPs)^36^. There are six members in the IGFBP protein family (numbered 1 through 6) and their molecular weight varies from 24 to 45 kDa^37^. The primary role of IGFBPs is to extend the half-life of IGFs in plasma^38,55^. However, studies have shown that IGFBPs can also inhibit the binding of IGF1 and IGF2 to their respective receptors^39,40^.

Vitamin D and IGFBPs have been suggested to act synergistically to affect insulin sensitivity, although the mechanism remains elusive^40,41^. IGFBPs have also been found to reflect risk for coronary heart disease and stroke as part of a randomised control trial using hormone therapy^42^. A recent study demonstrated the relationships between IGF1 and its binding proteins, with cardiometabolic risk in hypertensive perimenopausal females^43^. Furthermore, it has been reported in patients with T2DM, that the IGF system is strongly associated with cardiovascular disease damage^44^ and may constitute alternative risk factor markers^45^. In this study, the non-targeted high-precision serum proteomics analysis of a cross-sectional discovery cohort with bioinformatics interpretation, literature research and biostatistical assessment, identified a novel sexually dimorphic correlation of IGFBP-2 and IGFBP-3 with vitamin D status in non-diabetic males and females with obesity (Fig. 4a). Additionally, the IGFBP-2 and IGFBP-3 proteomic findings were verified for relative quantitative accuracy with Luminex ELISA against a statistically significant number of samples from an independent cohort (Fig. 4b) but with analogous clinical conditions with the discovery cohort (Tables 1 and 2). 

Although the precise function of IGFBP-2 in cardiometabolic pathophysiology is unclear, it has been implicated in the decrease of the biological activity of IGF1, thus regulating insulin sensitivity^46^. A study by Hedbacker et al.^47^ showed that the mRNA expression for IGFBP2 mediated the regulation of glucose metabolism, in response to the administration of physiologic and extra-physiologic doses of leptin in mice. In particular, high mRNA levels of IGFBP2 were associated with reduced blood glucose in wild type, as well as diabetic mice, and also suppressed hepatic glucose production and reduced expression of genes involved in hepatic fatty acid synthesis and gluconeogenesis. This study suggested that the induction of IGFBP2 as a result of leptin administration might play a preventive role in the pathogenesis of T2DM.

IGFBP-3 is the most prominent member of the IGFBP family, transporting 70–90% of the circulating IGF-1 and -2^39^. Serum IGFBP-3 has been shown to increase as a result of vitamin D administration^48^. Vitamin D may increase IGFBP-3 levels either through direct transcriptional induction in the liver, since IGFBP-3 is a transcriptional target of the vitamin D receptor^49^, or through an indirect enhancement of growth hormone stimulation^50^. IGFBP-3 decreases insulin-mediated uptake of glucose in the adipocytes^51^ and inhibits adipogenesis^52^.

We have previously shown that the serum proteomic profile of men is distinct from that of women, with men over-expressing proteins associated with an increased risk of cardiovascular disease^25^. Furthermore, sex hormones have been shown to affect circulating IGFBP levels^53,54^. Finally, we have described a sex-specific effect of vitamin D administration on serum proteins related to cardiovascular risk^24^. The above-mentioned associations could partly explain the results of the present study on a sexually dimorphic correlation of IGFBP-2 and IGFBP-3 with vitamin D status.

Despite the significant statistical power of this pilot study, the present findings should be examined in larger cross-sectional cohorts and randomised placebo-controlled studies to verify their translational importance. One study limitation is that some of the study participants were borderline hypertensive (systolic blood pressure up to 145 mmHg), an anticipated trend within a cohort of adults with overweight/obesity. Another study limitation is that serum IGF-1 levels were not measured in the present cohort. However, examining the association between IGF-1 levels and sex, vitamin D status and BMI constitutes a future perspective.

In conclusion, IGFBP-2 and IGFBP-3 were found to correlate with vitamin D status in males and female adults with obesity, respectively. In this population group, IGFBP-2 and IGFBP-3 warrant further examination as potential sexually dimorphic serological markers linking vitamin D status with cardiometabolic outcomes.

## Electronic supplementary material

Supplementary Table 1. Differentially expressed proteins in high vs. low vitamin D status (males)

Supplementary Table 2. Differentially expressed proteins in high vs. low vitamin D status (females)
