# Supplementary material for: Sex-specific correlation of IGFBP-2 and IGFBP-3 with vitamin D status in adults with obesity: a cross-sectional serum proteomics study
Source: Nutr Diabetes. 2018 Oct 4;8:54. doi: 10.1038/s41387-018-0063-8 (PMC6172285; doi:10.1038/s41387-018-0063-8)
Supplement: Supplementary file 1 — Supplementary Table 1. Differentially expressed proteins in high vs. low vitamin D status (males) [file 41387_2018_63_MOESM1_ESM.pdf]

pressed proteins in high vs. low vitD status (males)

ccessi	Description	Σ# Unique Peptides	Mean iTRAQ log2ratio high vs. lkow vitD status	117/113	117/114	117/115	117/116	118/113	118/114	118/115	118/116	119/113	119/114	119/115	119/116	121/113	121/114	121/115	121/116	ttest
Q6PIV	Solute carrier family 25 member 34 OS=Homo sapiens GN=SLC25A34 PE=2 SV=1 -	1	2.4	2.80	2.44	2.39	1.32	2.74	2.39	2.36	1.29	4.30	4.00	3.89	2.81	1.87	1.54	1.52	0.43	0.00
8NDV	Alpha-N-acetylgalactosaminide alpha-2,6-sialyltransferase 3 OS=Homo sapiens	1	2.1	-1.15	-1.93	0.01	-0.24	3.29	2.53	4.49	4.25	2.58	1.88	3.75	3.50	2.37	1.62	3.58	3.32	0.00
96MC	Dynein regulatory complex protein 1 OS=Homo sapiens	1	1.6	3.14	2.79	-0.16	-2.14	3.37	3.04	0.11	-1.87	5.15	4.87	1.86	-0.13	3.57	3.25	0.32	-1.68	0.02
Q6P1J	Phospholipase B1, membrane-associated OS=Homo sapiens GN=PLB1 PE=1 SV=3 -	1	1.5	1.05	-0.36	1.02	0.66	0.82	-0.56	0.84	0.49	0.73	-0.60	0.72	0.35	5.22	3.84	5.25	4.88	0.01
Q9290	60S ribosomal protein L3-like OS=Homo sapiens GN=RPL3L PE=1 SV=3 -	1	1.5	2.37	2.06	2.40	1.81	1.98	1.69	2.05	1.47	0.97	0.73	1.01	0.42	1.48	1.20	1.55	0.96	0.00
P0851	Apolipoprotein(a) OS=Homo sapiens GN=LPA PE=1 SV=1 -	4	1.5	1.80	1.69	1.16	1.04	1.45	1.33	0.58	0.55	1.88	1.77	0.97	0.98	2.54	2.37	1.76	1.83	0.00
P5169	Amyloid-like protein 1 OS=Homo sapiens GN=APLP1 PE=1 SV=3 -	1	1.5	3.79	2.51	2.67	1.09	3.20	1.94	2.11	0.54	2.54	1.33	1.42	-0.16	1.30	0.05	0.23	-1.36	0.00
Q1468	Structural maintenance of chromosomes protein 1A OS=Homo sapiens GN=SMC1A PE=1 SV=2 -	1	1.4	1.55	2.92	1.85	1.85	-0.05	1.34	0.29	0.30	2.80	4.24	3.11	3.11	-0.61	0.80	-0.25	-0.27	0.00
Q1591	Zinc finger and BTB domain-containing protein 6 OS=Homo sapiens GN=ZBTB6 PE=2 SV=1 -	1	1.3	-0.01	-0.17	0.03	0.67	1.40	1.26	1.49	2.13	2.51	2.42	2.56	3.19	0.70	0.58	0.80	1.42	0.00
Q5S00	Leucine-rich repeat serine/threonine-protein kinase 2 OS=Homo sapiens GN=LRRK2	1	1.3	0.45	0.30	0.12	0.91	4.41	4.27	4.12	4.92	0.44	0.35	0.11	0.90	-0.14	-0.27	-0.42	0.36	0.02
P2600	Integrin alpha-3 OS=Homo sapiens GN=ITGA3 PE=1 SV=5 -	1	1.3	0.93	2.05	2.28	2.22	0.68	1.82	2.07	2.02	-0.73	0.47	0.63	0.57	0.35	1.51	1.76	1.69	0.00
Q6NT0	Tigger transposable element-derived protein 7 OS=Homo sapiens GN=TIGD7 PE=2 SV=1 -	1	1.2	1.92	0.27	1.12	0.39	4.48	2.71	3.72	3.00	0.99	-0.59	0.20	-0.54	1.37	-0.25	0.62	-0.12	0.01
O9496	WD repeat-containing protein 47 OS=Homo sapiens GN=WDR47	1	1.2	1.58	1.93	0.19	1.86	2.15	2.51	0.80	2.47	1.41	1.83	0.03	1.69	0.29	0.68	-1.04	0.61	0.00
Q8N74	Killer cell immunoglobulin-like receptor 3DL3 OS=Homo sapiens GN=KIR3DL3 PE=2	1	1.2	3.74	2.29	0.98	2.64	3.44	2.01	0.71	2.38	1.44	0.06	-1.32	0.34	1.35	-0.07	-1.36	0.29	0.01
A6NFI	Zinc finger protein 316 OS=Homo sapiens GN=ZNF316 PE=1 SV=1 -	1	1.2	0.56	1.64	0.31	1.10	1.51	2.61	1.31	2.10	0.89	2.04	0.65	1.43	0.28	1.39	0.09	0.86	0.00
Q1466	UPF0378 protein KIAA0100 OS=Homo sapiens GN=KIAA0100	1	1.1	0.97	1.35	0.29	0.59	2.27	2.66	1.62	1.92	0.29	0.74	-0.38	-0.09	1.68	2.09	1.05	1.33	0.00
9UKA	F-box/LRR-repeat protein 4 OS=Homo sapiens GN=FBXL4 PE=1 SV=2 -	1	1.1	1.40	1.22	1.14	0.94	1.36	1.20	1.14	0.95	1.93	1.82	1.67	1.47	0.59	0.44	0.39	0.17	0.00

Q70CQ	Ubiquitin carboxyl-terminal hydrolase 34 OS=Homo sapiens GN=USP34 PE=1 SV=2 -	1	1.1	-0.38	1.36	2.35	2.04	-1.65	0.16	1.12	0.77	1.14	2.95	3.87	3.59	-1.64	0.14	1.14	0.85	0.01
P2198	Protein-glutamine gamma-glutamyltransferase 2 OS=Homo sapiens GN=TGM2 PE=1 SV=2 -	1	1.1	-1.71	2.36	2.42	1.49	-1.41	2.67	2.75	1.83	-2.29	1.84	1.84	0.91	-1.74	2.36	2.44	1.50	0.03
Q86UK	ATP-binding cassette sub-family A member 12 OS=Homo sapiens GN=ABCA12 PE=1	1	1.0	1.28	2.10	0.88	-1.34	1.13	1.97	0.77	-1.45	3.51	4.40	3.11	0.88	0.35	1.21	0.00	-2.23	0.04
P4324	Matrin-3 OS=Homo sapiens GN=MATR3 PE=1 SV=2 -	1	1.0	-0.45	0.63	1.01	0.28	1.39	2.50	2.89	2.17	0.62	1.78	2.10	1.36	-0.80	0.32	0.72	-0.03	0.00
Q86YW	Trem-like transcript 1 protein OS=Homo sapiens GN=TREML1	3	1.0	0.23	2.17	0.95	1.31	-0.06	1.71	0.72	0.74	0.57	2.59	1.03	0.89	-0.22	1.78	0.74	1.29	0.00
Q3KR1	Pleckstrin homology domain-containing family G member 6 OS=Homo sapiens GN=PLEKHG6	1	1.0	2.15	3.93	1.81	2.76	-0.74	1.06	-1.05	-0.09	-0.49	1.36	-0.83	0.11	1.06	2.88	0.77	1.71	0.01
P4637	Large proline-rich protein BAG6 OS=Homo sapiens GN=BAG6 PE=1 SV=2 -	2	1.0	1.89	0.83	1.99	2.97	2.69	1.65	2.56	3.24	-0.91	-1.90	-0.45	0.46	0.16	-0.78	0.23	1.38	0.02
Q8TBZ	Uncharacterized protein C7orf62 OS=Homo sapiens GN=C7orf62	1	1.0	1.05	0.43	0.16	-0.56	0.92	0.31	0.06	-0.65	2.03	1.47	1.14	0.42	3.00	2.41	2.16	1.42	0.00
Q5T6X	Protein FAM162B OS=Homo sapiens GN=FAM162B PE=2	1	1.0	-0.15	-0.86	0.17	0.22	0.70	0.00	1.05	1.12	3.12	2.48	3.44	3.49	0.14	-0.55	0.50	0.55	0.01
Q5FBB	Shugoshin-like 1 OS=Homo sapiens GN=SGOL1 PE=1 SV=1 -	1	1.0	0.88	0.65	1.33	1.45	0.95	0.74	1.44	1.57	0.72	0.57	1.18	1.30	0.41	0.22	0.92	1.02	0.00
P3061	Pyruvate kinase PKLR OS=Homo sapiens GN=PKLR PE=1 SV=2 -	1	0.9	0.92	-2.09	1.55	1.11	1.82	-1.17	2.48	2.05	1.43	-1.51	2.06	1.62	1.70	-1.28	2.38	1.92	0.03
Q1469	Neutral alpha-glucosidase AB OS=Homo sapiens GN=GANAB PE=1 SV=3	1	0.9	1.46	0.64	1.28	1.41	1.33	0.53	1.19	1.08	1.51	0.76	1.34	1.72	0.47	-0.40	0.09	0.45	0.00
Q86WI	Fibrocystin-L OS=Homo sapiens GN=PKHD1L1 PE=2 SV=2 -	1	0.9	0.87	1.32	0.15	1.22	0.92	1.39	0.24	1.31	1.12	1.64	0.40	1.46	0.64	1.12	-0.02	1.03	0.00
Q9BUB	MAP kinase-interacting serine/threonine-protein kinase 1 OS=Homo sapiens GN=MKNK1	1	0.9	0.82	1.08	0.27	-0.63	1.08	1.36	0.57	-0.33	1.99	2.32	1.45	0.53	1.46	1.75	0.96	0.03	0.00
P5117	Amiloride-sensitive sodium channel subunit gamma OS=Homo sapiens GN=SCNN1G	2	0.9	-0.34	-0.87	0.58	0.38	0.31	-0.20	1.27	1.07	0.87	0.41	1.80	1.59	1.63	1.13	2.60	2.38	0.00
Q5VWX	KH domain-containing, RNA-binding, signal transduction-associated protein 2 OS=Homo sapiens GN=KHDRBS2	1	0.9	0.15	1.12	0.81	-0.07	0.02	1.02	0.72	-0.15	1.62	2.67	2.29	1.41	0.32	1.32	1.03	0.14	0.00
O4326	Centromere/kinetochore protein zw10 homolog OS=Homo sapiens GN=ZW10 PE=1 SV=3 -	1	0.9	1.16	0.29	2.53	2.68	-1.47	-2.31	-0.06	0.10	0.50	-0.30	1.87	2.02	1.27	0.44	2.69	2.83	0.03
O9516	Elongator complex protein 1 OS=Homo sapiens GN=IKBKAP	1	0.9	0.49	0.78	-0.27	0.65	2.34	2.65	1.62	2.55	0.41	0.77	-0.34	0.58	0.54	0.87	-0.16	0.75	0.00
Q0298	Complement factor H-related protein 3 OS=Homo sapiens GN=CFHR3 PE=1 SV=2 -	2	0.9	0.14	2.20	0.44	0.00	0.70	2.83	1.05	0.62	0.87	3.03	1.15	0.81	-0.48	1.54	-0.16	-0.52	0.00
Q9NYA	Myotubularin-related protein 4 OS=Homo sapiens GN=MTMR4	1	0.9	-0.50	-0.93	-1.23	-0.41	2.80	2.40	2.11	2.94	2.21	1.86	1.49	2.30	0.00	-0.39	-0.67	0.13	0.03
O1480	Ras-related protein M-Ras OS=Homo sapiens GN=MRAS PE=1 SV=2 -	1	0.9	-0.65	1.64	2.59	1.08	-1.48	0.83	1.81	0.30	-1.37	0.98	1.88	0.36	-0.31	2.00	2.98	1.45	0.02

O0039	Sulfhydryl oxidase 1 OS=Homo sapiens GN=QSOX1 PE=1 SV=3 -	2	0.9	-0.56	0.88	0.89	-0.71	-0.33	1.12	1.16	-0.44	0.54	2.05	2.00	0.39	0.94	2.40	2.44	0.82	0.00
Q9UKN	Unconventional myosin-XV OS=Homo sapiens GN=MYO15A PE=1	2	0.8	0.30	0.12	0.65	1.19	0.18	-0.37	0.24	0.27	0.24	0.39	0.71	0.36	1.95	3.38	3.81	-0.09	0.01
Q9218	Alpha-N-acetylneuraminide alpha-2,8-sialyltransferase OS=Homo sapiens	1	0.8	0.62	1.08	0.10	0.29	1.08	1.56	0.60	0.79	2.48	3.01	1.97	2.15	-0.56	-0.07	-1.03	-0.86	0.01
Q9972	BRCA1-associated RING domain protein 1 OS=Homo sapiens GN=BARD1 PE=1 SV=2 -	1	0.8	-0.15	-0.30	-0.12	-0.09	0.68	0.55	0.75	0.79	0.63	0.55	0.66	0.69	2.12	2.00	2.20	2.22	0.00
Q9P2D	Protein unc-79 homolog OS=Homo sapiens GN=UNC79 PE=2 SV=4 -	2	0.8	0.65	1.71	-0.06	0.54	1.31	2.94	0.95	1.66	1.32	1.28	1.31	0.87	-0.75	0.20	-0.85	-0.04	0.00
Q96JB	Dynein heavy chain 8, axonemal OS=Homo sapiens GN=DNAH8	1	0.8	0.76	0.68	1.03	0.71	0.97	0.90	1.28	0.96	0.19	0.18	0.47	0.14	1.09	1.03	1.41	1.07	0.00
Q1058	ADP-ribosyl cyclase/cyclic ADP-ribose hydrolase 2 OS=Homo sapiens	7	0.8	0.92	-0.20	0.44	1.05	1.40	0.37	0.97	1.75	1.08	0.16	0.74	1.39	0.86	-0.14	0.52	1.35	0.00
Q8N6Q	Methyltransferase-like protein 25 OS=Homo sapiens GN=METTL25	1	0.8	-0.12	-0.26	1.15	0.30	1.64	1.53	2.96	2.11	-0.12	-0.18	1.16	0.31	0.11	0.01	1.44	0.57	0.00
Q9P2S	WD repeat-containing protein WRAP73 OS=Homo sapiens GN=WRAP73 PE=2	1	0.8	1.05	1.19	0.81	1.18	0.37	0.53	0.17	0.55	-0.55	-0.34	-0.79	-0.42	2.17	2.34	1.98	2.34	0.01
P13647	Keratin, type II cytoskeletal 5 OS=Homo sapiens GN=KRT5 PE=1	3	0.8	2.03	2.57	1.53	0.54	0.48	1.04	0.03	-0.21	1.52	2.13	1.03	-0.08	0.26	0.83	-0.18	-1.05	0.01
Q96QK	Vacuolar protein sorting-associated protein 35 OS=Homo sapiens GN=VPS35 PE=1 SV=2 -	1	0.8	-0.26	0.27	0.62	1.10	0.95	1.50	1.87	2.35	-0.17	0.44	0.72	1.19	-0.30	0.26	0.63	1.09	0.00
Q9P27	Teneurin-3 OS=Homo sapiens GN=TENM3 PE=2 SV=3 -	1	0.8	-1.02	-0.92	-0.73	-1.58	2.22	2.35	2.55	1.70	1.15	1.33	1.45	0.59	0.78	0.91	1.12	0.25	0.03
P12883	Myosin-7 OS=Homo sapiens GN=MYH7 PE=1 SV=5 - [MYH7_HUMAN]	1	0.8	-1.56	-0.77	0.07	-0.35	1.54	2.35	3.21	2.79	0.14	0.99	1.77	1.34	-0.78	0.04	0.90	0.46	0.04
Q09MP	RAD51-associated protein 2 OS=Homo sapiens GN=RAD51AP2	1	0.8	0.02	-0.25	0.39	1.77	-0.42	-0.66	-0.01	1.38	-0.48	-0.67	-0.10	1.28	1.97	1.74	2.39	3.76	0.04
Q9Y6H	Synphilin-1 OS=Homo sapiens GN=SNCAIP PE=1 SV=2 -	1	0.8	1.03	1.50	0.37	-0.83	1.77	2.26	1.15	-0.04	1.92	2.46	1.26	0.07	0.27	0.78	-0.33	-1.54	0.02
H7BZ5	Putative ciliary rootlet coiled-coil protein-like 3 protein OS=Homo sapiens PE=5 SV=2 -	1	0.7	0.92	1.65	0.32	-0.11	0.45	1.20	-0.12	-0.54	2.86	3.66	2.26	1.82	-0.40	0.37	-0.95	-1.39	0.05
P05019	Insulin-like growth factor I OS=Homo sapiens GN=IGF1 PE=1 SV=1 -	5	0.7	1.40	1.77	0.19	-0.73	2.04	2.54	0.96	0.17	2.32	2.66	1.38	0.42	-0.07	0.17	-1.26	-2.01	0.05
Q1410	Heterogeneous nuclear ribonucleoprotein D0 OS=Homo sapiens GN=HNRNPD PE=1	1	0.7	0.68	0.82	1.13	-0.11	1.14	1.29	1.62	0.39	1.15	1.35	1.60	0.36	0.14	0.30	0.63	-0.62	0.00
Q1364	Four and a half LIM domains protein 1 OS=Homo sapiens GN=FHL1 PE=1 SV=4 -	1	0.7	0.63	0.31	0.83	1.35	0.32	0.01	0.55	1.08	-0.54	-0.79	-0.34	0.18	1.88	1.59	2.13	2.64	0.01
O0026	Transcription elongation factor SPT5 OS=Homo sapiens GN=SUPT5H PE=1 SV=1 -	1	0.7	0.81	0.43	1.55	0.28	0.08	-0.29	0.85	-0.41	0.97	0.65	1.70	0.43	1.21	0.85	1.99	0.71	0.00
Q0359	Complement factor H-related protein 1 OS=Homo sapiens GN=CFHR1 PE=1 SV=2 -	2	0.7	0.67	0.92	0.03	0.31	0.86	1.22	0.41	0.53	1.41	1.59	0.64	1.10	0.64	1.04	-0.01	0.40	0.00

P00480	Ornithine carbamoyltransferase, mitochondrial OS=Homo sapiens GN=OTC PE=1	2	0.7	1.39	1.02	1.17	1.81	0.01	-0.34	-0.17	0.48	1.16	0.86	0.95	1.59	0.43	0.09	0.26	0.89	0.00
Q1575	Probable E3 ubiquitin-protein ligase HERC1 OS=Homo sapiens GN=HERC1 PE=1 SV=2 -	1	0.7	0.43	0.16	1.08	1.01	0.61	0.36	1.30	1.23	0.08	-0.12	0.74	0.66	0.66	0.42	1.37	1.28	0.00
Q9UKN	Mucin-12 OS=Homo sapiens GN=MUC12 PE=1 SV=2 -	1	0.7	0.75	0.87	0.70	0.41	0.74	0.88	0.73	0.44	0.73	0.92	0.69	0.39	0.76	0.92	0.77	0.46	0.00
P11279	Lysosome-associated membrane glycoprotein 1 OS=Homo sapiens GN=LAMP1 PE=1 SV=3 -	1	0.7	0.16	0.47	1.26	0.80	0.03	0.36	1.16	0.71	0.19	0.57	1.29	0.83	0.29	0.63	1.44	0.96	0.00
Q9NUU	ATP-dependent RNA helicase DDX19A OS=Homo sapiens GN=DDX19A PE=1 SV=1	1	0.7	0.90	1.43	0.62	0.89	0.95	1.50	0.71	0.98	0.39	0.99	0.11	0.37	0.21	0.78	-0.02	0.23	0.00
Q96EP	E3 ubiquitin-protein ligase CHFR OS=Homo sapiens GN=CHFR PE=1 SV=2 -	1	0.7	0.36	1.24	-0.09	-0.01	0.82	1.72	0.41	0.50	0.94	1.90	0.50	0.58	0.48	1.40	0.08	0.15	0.00
P13473	Lysosome-associated membrane glycoprotein 2 OS=Homo sapiens GN=LAMP2 PE=1 SV=2 -	1	0.7	0.24	-0.04	0.78	0.90	0.55	0.29	1.13	1.26	0.22	0.02	0.77	0.89	0.72	0.48	1.32	1.43	0.00
P78560	Death domain-containing protein CRADD OS=Homo sapiens GN=CRADD PE=1 SV=1	1	0.7	0.58	0.34	0.43	0.89	0.19	-0.03	0.08	0.54	-0.21	-0.38	-0.36	0.09	2.19	1.98	2.09	2.53	0.01
P69891	Hemoglobin subunit gamma-1 OS=Homo sapiens GN=HBG1 PE=1	1	0.7	1.21	1.79	1.25	1.22	0.29	0.89	0.37	0.34	-0.11	0.54	-0.06	-0.10	0.62	1.23	0.71	0.67	0.00
Q8NHV	Protein NEDD1 OS=Homo sapiens GN=NEDD1 PE=1 SV=1 -	1	0.7	0.29	1.36	0.57	1.09	0.52	1.61	0.84	1.37	-0.30	0.83	0.64	0.50	-0.19	0.91	0.14	0.65	0.00
P01595	Ig kappa chain V-I region Bi OS=Homo sapiens PE=1 SV=1 -	1	0.7	1.36	0.16	0.94	0.54	1.51	0.33	1.13	0.73	1.19	0.07	0.75	0.38	1.01	-0.16	0.64	0.23	0.00
P06310	Ig kappa chain V-II region RPMI 6410 OS=Homo sapiens PE=4	2	0.7	0.68	0.57	0.65	0.50	0.28	0.31	0.23	0.32	1.74	1.67	1.75	1.52	0.19	0.08	0.12	0.17	0.00
P01876	Ig alpha-1 chain C region OS=Homo sapiens GN=IGHA1 PE=1 SV=2 -	4	0.7	0.64	0.58	0.86	0.72	0.70	0.65	0.82	0.74	0.19	0.24	0.58	0.43	0.84	0.79	1.06	0.87	0.00
Q494V	Coiled-coil domain-containing protein 37 OS=Homo sapiens GN=CCDC37 PE=2	1	0.7	0.75	0.62	1.29	1.56	0.04	-0.07	0.62	0.89	-0.31	-0.36	0.24	0.50	0.84	0.74	1.43	1.68	0.00
A4D0V	Cadherin-like and PC-esterase domain-containing protein 1 OS=Homo sapiens	1	0.7	0.04	0.61	1.10	0.69	-0.07	0.52	1.03	0.63	0.36	1.00	1.43	1.02	-0.08	0.52	1.03	0.61	0.00
Q96EY	Multivesicular body subunit 12A OS=Homo sapiens GN=MVB12A	1	0.6	0.26	0.85	0.35	-0.29	0.41	1.02	0.54	-0.09	0.97	1.63	1.07	0.43	0.74	1.36	0.88	0.23	0.00
P00973	2'-5'-oligoadenylate synthase 1 OS=Homo sapiens GN=OAS1 PE=1	1	0.6	0.43	0.52	0.50	0.64	0.61	0.72	0.72	0.87	0.55	0.70	0.62	0.76	0.53	0.65	0.65	0.77	0.00
Q9UP9	Solute carrier family 12 member 4 OS=Homo sapiens GN=SLC12A4	1	0.6	1.28	0.36	-0.30	1.11	1.61	0.71	0.07	1.48	1.20	0.35	-0.38	1.03	1.04	0.16	-0.49	0.91	0.00
P14151	L-selectin OS=Homo sapiens GN=SELL PE=1 SV=2 -	3	0.6	0.66	0.36	0.79	1.16	0.49	0.21	0.70	1.04	0.26	-0.12	0.25	0.70	0.85	0.39	0.94	1.45	0.00
Q9UHV	Mediator of RNA polymerase II transcription subunit 13 OS=Homo sapiens	1	0.6	0.79	0.63	0.83	0.79	1.09	0.95	1.17	1.14	-0.17	-0.26	-0.12	-0.17	0.84	0.72	0.94	0.88	0.00
Q8IWZ	SURP and G-patch domain-containing protein 1 OS=Homo sapiens GN=SUGP1 PE=1 SV=2 -	1	0.6	-0.89	0.79	-0.22	-0.33	0.40	2.10	1.12	1.01	0.83	2.58	1.51	1.40	-0.83	0.88	-0.10	-0.23	0.02

Q1501	Homocysteine-responsive endoplasmic reticulum-resident ubiquitin-like domain member 1 protein OS=Homo sapiens	1	0.6	0.60	-0.79	0.74	0.45	0.57	-0.80	0.75	0.46	0.99	-0.34	1.13	0.83	1.67	0.31	1.86	1.56	0.01
P0177	Ig heavy chain V-III region TUR OS=Homo sapiens PE=1 SV=1 -	1	0.6	1.21	0.43	0.74	1.22	0.82	0.04	0.47	0.86	0.19	-0.45	-0.20	0.31	1.48	0.54	0.87	1.38	0.00
P0170	Ig lambda chain V-II region BOH OS=Homo sapiens PE=1 SV=1 -	1	0.6	0.02	-0.06	0.57	-0.11	0.84	0.34	0.73	0.25	1.79	1.08	1.65	0.96	0.68	0.21	0.65	0.14	0.00
O9539	Bone morphogenetic protein 10 OS=Homo sapiens GN=BMP10	1	0.6	0.40	0.42	0.14	0.20	0.54	0.58	0.32	0.39	0.56	0.65	0.31	0.37	1.27	1.32	1.06	1.11	0.00
Q8IZ2	Zinc finger protein 34 OS=Homo sapiens GN=ZNF34 PE=1 SV=3 -	1	0.6	1.10	0.30	0.46	0.71	1.11	0.32	0.50	0.75	1.02	0.29	0.38	0.63	0.94	0.17	0.35	0.58	0.00
Q4G0U	Cilia- and flagella-associated protein 221 OS=Homo sapiens GN=CFAP221 PE=1	2	0.6	1.54	0.07	1.28	1.09	1.02	-0.42	0.80	0.62	0.78	-0.62	0.52	0.33	1.13	-0.30	0.92	0.72	0.00
P25311	Zinc-alpha-2-glycoprotein OS=Homo sapiens GN=AZGP1 PE=1 SV=2 -	23	0.6	0.61	0.59	0.90	1.31	0.35	0.40	0.66	1.06	0.06	0.17	0.37	0.83	0.20	0.28	0.59	1.09	0.00
Q6ZP0	RNA-binding protein 44 OS=Homo sapiens GN=RBM44 PE=2 SV=2 -	1	0.6	-0.03	0.81	0.21	0.69	0.06	0.92	0.34	0.83	-0.63	0.28	-0.38	0.10	1.08	1.95	1.38	1.85	0.01
P2070	Lamin-B1 OS=Homo sapiens GN=LMNB1 PE=1 SV=2 -	1	0.6	1.80	1.74	1.89	2.07	-0.18	-0.22	-0.05	0.13	0.03	0.04	0.13	0.30	0.33	0.30	0.48	0.64	0.01
P2158	Kit ligand OS=Homo sapiens GN=KITLG PE=1 SV=1 -	1	0.6	-0.22	0.18	-0.27	-0.47	0.99	1.41	0.98	0.79	1.23	1.70	1.19	0.99	0.15	0.59	0.16	-0.05	0.00
P80362	Ig kappa chain V-I region WAT OS=Homo sapiens PE=1 SV=1 -	1	0.6	1.00	2.30	-0.80	1.06	1.21	1.31	-0.84	1.15	0.55	0.71	-0.81	0.46	0.45	1.79	-0.80	0.55	0.03
P2791	Properdin OS=Homo sapiens GN=CFP PE=1 SV=2 - [PROP_HUMAN]	4	0.6	-0.01	0.09	0.60	1.37	0.10	0.17	0.65	1.35	-0.48	-0.23	0.04	0.87	0.67	0.84	1.36	1.87	0.00
Q9HCK	DBF4-type zinc finger-containing protein 2 OS=Homo sapiens GN=ZDBF2 PE=1 SV=3 -	1	0.6	0.07	0.87	-0.06	0.22	0.51	1.33	0.42	0.70	0.88	1.75	0.75	1.03	-0.08	0.75	-0.16	0.10	0.00
Q9P2F	Storkhead-box protein 2 OS=Homo sapiens GN=STOX2 PE=2 SV=2 -	1	0.6	0.51	1.69	-0.11	0.63	0.73	2.48	0.01	1.01	0.25	1.59	0.01	0.49	-0.11	0.53	-0.81	0.17	0.01
Q9UFH	Dynein heavy chain 17, axonemal OS=Homo sapiens GN=DNAH17	1	0.6	0.53	0.20	0.94	0.53	0.75	1.26	1.01	0.54	0.28	0.69	0.98	0.49	0.31	-0.18	0.39	0.30	0.00
P1806	Insulin-like growth factor-binding protein 2 OS=Homo sapiens GN=IGFBP2 PE=1 SV=2 -	7	0.6	0.12	0.47	-0.34	-0.31	1.51	2.24	1.21	1.34	1.00	1.46	0.50	0.44	-0.40	0.42	-0.43	-0.32	0.02
Q9H3U	Protein unc-45 homolog A OS=Homo sapiens GN=UNC45A PE=1	1	0.6	1.07	1.13	0.60	0.76	0.77	0.85	0.34	0.50	1.68	1.80	1.21	1.37	-0.66	-0.57	-1.08	-0.93	0.03
O0076	Acetyl-CoA carboxylase 2 OS=Homo sapiens GN=ACACB PE=1 SV=3	1	0.6	0.39	0.71	-0.28	-0.35	1.52	1.86	0.89	0.81	0.88	1.27	0.22	0.14	0.44	0.79	-0.18	-0.27	0.00
Q9UNN	Endothelial protein C receptor OS=Homo sapiens GN=PROCR	3	0.6	0.40	0.10	0.79	0.89	0.29	-0.06	0.60	0.69	-0.29	-0.58	0.37	0.19	1.16	0.94	1.69	1.60	0.00
P35527	Keratin, type I cytoskeletal 9 OS=Homo sapiens GN=KRT9 PE=1	12	0.5	1.99	1.86	1.97	1.37	0.29	0.13	0.17	-0.41	1.00	1.17	1.01	0.38	-0.41	-0.16	-0.54	-1.04	0.04
P15927	Replication protein A 32 kDa subunit OS=Homo sapiens GN=RPA2 PE=1 SV=1 - [RFA2_HUMAN]	1	0.5	0.56	1.29	-0.18	1.50	-0.05	0.69	-0.76	0.93	0.49	1.29	-0.24	1.44	0.18	0.94	-0.51	1.16	0.01
P01607	Ig kappa chain V-I region Rei OS=Homo sapiens PE=1 SV=1 -	2	0.5	0.22	0.60	0.40	-0.17	1.18	1.56	1.42	1.05	0.69	1.02	0.92	0.54	-0.39	0.07	0.06	-0.41	0.00

O7559	Peptidoglycan recognition protein 1 OS=Homo sapiens GN=PGLYRP1	1	0.5	0.44	1.39	-0.01	1.02	0.32	1.02	-0.09	0.62	0.02	0.94	-0.47	0.62	0.46	1.49	0.07	0.88	0.00
P0277	Vitamin D-binding protein OS=Homo sapiens GN=GC PE=1 SV=1 -	37	0.5	0.40	0.79	0.98	1.10	0.13	0.51	0.64	0.82	-0.26	0.30	0.30	0.48	0.15	0.52	0.74	0.88	0.00
Q0148	Ankyrin-2 OS=Homo sapiens GN=ANK2 PE=1 SV=4 - [ANK2_HUMAN]	1	0.5	-0.61	-0.47	0.50	0.31	-0.58	-0.42	0.57	0.38	0.51	0.72	1.63	1.43	0.54	0.72	1.71	1.50	0.02
Q9999	A-kinase anchor protein 9 OS=Homo sapiens GN=AKAP9 PE=1 SV=3 -	3	0.5	0.50	0.22	0.08	-0.36	2.49	1.77	1.66	1.34	0.58	0.36	0.21	-0.20	0.18	-0.07	-0.23	-0.11	0.02
P5160	Methyl-CpG-binding protein 2 OS=Homo sapiens GN=MECP2	1	0.5	0.10	1.43	1.02	-0.03	-0.22	1.13	0.74	-0.31	-0.02	1.38	0.91	-0.15	0.04	1.40	1.01	-0.05	0.01
Q1304	Protein flightless-1 homolog OS=Homo sapiens GN=FLII PE=1	1	0.5	0.04	0.64	0.47	1.20	-0.61	0.01	-0.14	0.59	0.96	1.64	1.40	2.12	-0.57	0.07	-0.08	0.63	0.02
P0161	Ig kappa chain V-II region Cum OS=Homo sapiens PE=1 SV=1 -	2	0.5	0.26	0.50	0.35	0.71	1.18	1.43	1.14	1.87	0.20	0.50	0.24	0.72	-0.50	-0.22	-0.40	0.20	0.01
Q9Y5E	Protocadherin beta-2 OS=Homo sapiens GN=PCDHB2 PE=1	1	0.5	-0.80	0.21	-0.86	1.36	0.22	1.24	0.69	2.41	-0.78	0.30	0.01	1.37	-0.22	0.82	0.22	1.97	0.05
Q8IX0	Protein KIBRA OS=Homo sapiens GN=WWC1 PE=1 SV=1 -	1	0.5	0.14	0.59	0.31	-0.55	0.77	1.24	0.98	0.13	0.96	1.48	1.14	0.28	0.14	0.63	0.37	-0.50	0.00
Q1381	Spectrin alpha chain, non-erythrocytic 1 OS=Homo sapiens GN=SPTAN1 PE=1 SV=3 -	3	0.5	0.48	0.65	1.88	1.92	-0.28	-0.48	0.42	0.59	0.08	0.32	0.65	0.68	0.29	0.43	0.19	0.27	0.01
Q6Q75	Sperm-associated antigen 17 OS=Homo sapiens GN=SPAG17	1	0.5	0.41	0.66	0.36	0.35	0.56	0.95	0.51	0.42	0.33	0.99	0.26	0.61	0.25	0.89	0.25	0.29	0.00
P0966	Pro-cathepsin H OS=Homo sapiens GN=CTSH PE=1 SV=4 -	1	0.5	0.77	0.42	0.59	0.63	0.84	0.51	0.70	0.75	0.98	0.70	0.81	0.84	0.01	-0.30	-0.11	-0.09	0.00
P0177	Ig heavy chain V-III region KOL OS=Homo sapiens PE=1 SV=1 -	1	0.5	0.44	0.49	0.58	0.92	0.42	0.47	0.45	1.20	0.34	0.21	0.49	0.82	0.04	0.11	0.26	0.78	0.00
Q2NKX	DNA excision repair protein ERCC-6-like OS=Homo sapiens GN=ERCC6L PE=1 SV=1	1	0.5	0.08	0.04	-1.14	-0.51	0.93	0.91	-0.25	0.39	0.79	0.82	-0.43	0.20	1.96	1.95	0.79	1.41	0.04
.8MV2	Serpin E3 OS=Homo sapiens GN=SERPINE3 PE=2 SV=2 -	1	0.5	0.31	0.33	0.86	1.20	0.07	0.11	0.65	1.00	-0.87	-0.78	-0.31	0.02	0.94	1.00	1.54	1.87	0.02
O6065	UDP-glucuronosyltransferase 1-9 OS=Homo sapiens	1	0.5	1.39	0.80	0.79	0.96	0.58	0.02	0.02	0.20	0.53	0.02	-0.06	0.10	1.01	0.46	0.46	0.62	0.00
P3698	Complement factor H-related protein 2 OS=Homo sapiens GN=CFHR2 PE=1 SV=1 -	7	0.5	0.60	1.25	-0.26	0.34	0.84	1.47	0.09	0.66	0.93	1.51	0.13	0.61	0.02	0.71	-0.77	-0.23	0.01
Q1651	Serine/threonine-protein kinase N2 OS=Homo sapiens GN=PKN2 PE=1	1	0.5	0.86	-0.33	0.85	0.12	0.72	-0.46	0.74	0.02	0.89	-0.23	0.88	0.15	1.36	0.21	1.41	0.67	0.00
O7501	Low affinity immunoglobulin gamma Fc region receptor III-B OS=Homo sapiens	5	0.5	-0.41	0.41	0.45	0.62	-0.41	0.63	0.72	0.54	-0.04	0.93	0.94	0.95	-0.25	0.90	0.92	0.77	0.00
Q1500	PCNA-associated factor OS=Homo sapiens GN=KIAA0101 PE=1	1	0.5	0.12	-0.78	-0.75	0.70	1.20	0.32	0.37	1.83	1.21	0.38	0.35	1.79	0.50	-0.37	-0.32	1.12	0.03
O9521	Sorting nexin-4 OS=Homo sapiens GN=SNX4 PE=1 SV=1 -	1	0.5	0.67	0.81	1.16	-0.69	0.31	0.46	0.84	-1.00	0.64	0.84	1.13	-0.72	0.95	1.12	1.49	-0.37	0.02
Q9P2D	Dynein heavy chain 1, axonemal OS=Homo sapiens GN=DNAH1	1	0.5	0.09	-0.80	-0.26	-0.46	0.74	-0.12	0.43	0.24	0.58	-0.23	0.24	0.04	2.19	1.34	1.90	1.68	0.05
Q9UBQ	Exostosin-like 2 OS=Homo sapiens GN=EXTL2 PE=1 SV=1 -	1	0.5	0.70	-0.24	1.26	0.45	0.92	0.00	1.51	0.71	0.08	-0.79	0.64	-0.17	0.75	-0.16	1.35	0.53	0.01

P04264	Keratin, type II cytoskeletal 1 OS=Homo sapiens GN=KRT1 PE=1	20	0.5	1.48	1.68	1.25	0.73	0.51	0.72	0.43	-0.11	0.70	0.86	0.48	0.04	-0.14	-0.01	-0.29	-0.86	0.01
Q9H7D	Dedicator of cytokinesis protein 5 OS=Homo sapiens GN=DOCK5	1	0.5	0.19	0.43	0.41	-0.40	0.92	1.18	1.18	0.37	0.77	1.08	0.99	0.18	0.05	0.32	0.32	-0.50	0.00
P01606	Ig kappa chain V-I region OU OS=Homo sapiens PE=1 SV=1 -	2	0.5	0.34	0.65	0.44	0.54	0.57	0.89	0.70	0.81	-0.06	0.89	0.61	0.70	-0.09	0.25	0.07	0.15	0.00
P04180	Phosphatidylcholine-sterol acyltransferase OS=Homo sapiens GN=LCAT PE=1 SV=1 -	2	0.5	0.33	-0.60	0.24	-0.19	-0.09	-0.52	-0.14	-0.13	0.87	-0.01	0.65	0.36	2.00	1.08	1.96	1.52	0.04
Q13698	Voltage-dependent L-type calcium channel subunit alpha-1S OS=Homo sapiens	1	0.5	-0.24	0.19	-0.04	-0.17	0.29	0.74	0.53	0.41	0.51	1.01	0.72	0.58	0.50	0.96	0.75	0.60	0.00
P02790	Hemopexin OS=Homo sapiens GN=HPX PE=1 SV=2 - [HEMO_HUMAN]	33	0.5	0.43	0.25	0.83	0.86	0.13	-0.07	0.54	0.59	-0.09	-0.17	0.33	0.36	0.63	0.50	1.08	1.09	0.00
P01719	Ig lambda chain V-V region DEL OS=Homo sapiens PE=1 SV=1 -	2	0.5	0.25	1.34	0.80	0.94	0.03	1.07	0.23	0.46	-0.44	1.18	0.30	0.42	-0.33	0.82	0.10	0.03	0.00
Q4ZG55	Protein GREB1 OS=Homo sapiens GN=GREB1 PE=2 SV=1 -	2	0.4	0.35	0.91	1.19	1.33	-0.37	0.21	0.43	0.40	0.01	0.22	0.62	0.16	-0.16	0.43	0.80	0.65	0.00
Q9NYK	Toll-like receptor 7 OS=Homo sapiens GN=TLR7 PE=1 SV=1 -	1	0.4	0.66	0.69	-0.02	-0.34	1.03	1.08	0.39	0.07	0.78	0.88	0.10	-0.23	0.89	0.95	0.26	-0.08	0.00
Q96LM	Putative uncharacterized protein encoded by LINC00615 OS=Homo sapiens GN=LINC00615	1	0.4	0.75	1.12	0.41	0.52	0.33	0.72	0.03	0.15	0.35	0.79	0.01	0.12	0.47	0.87	0.18	0.28	0.00
P01034	Cystatin-C OS=Homo sapiens GN=CST3 PE=1 SV=1 - [CYTC_HUMAN]	4	0.4	0.64	0.59	0.41	0.09	0.84	0.77	0.61	0.28	0.82	0.85	0.52	0.33	0.24	0.27	0.17	-0.32	0.00
P01903	HLA class II histocompatibility antigen, DR alpha chain OS=Homo sapiens	1	0.4	0.37	1.10	1.35	0.58	0.30	1.05	1.32	0.55	0.05	0.85	1.04	0.26	-0.96	-0.19	0.08	-0.71	0.02
P04075	Fructose-bisphosphate aldolase A OS=Homo sapiens GN=ALDOA	1	0.4	0.32	1.07	1.00	-0.11	0.09	0.87	0.82	-0.29	0.19	1.01	0.88	-0.24	0.03	0.82	0.77	-0.36	0.00
Q8NDI	EH domain-binding protein 1 OS=Homo sapiens GN=EHBP1	1	0.4	0.61	1.17	0.04	0.58	1.09	1.67	0.56	1.10	-0.03	0.60	-0.59	-0.06	0.00	0.59	-0.52	0.00	0.02
P05067	Amyloid beta A4 protein OS=Homo sapiens GN=APP PE=1 SV=3 -	3	0.4	-0.17	-0.57	-0.12	-0.27	1.26	1.29	1.15	0.62	0.22	-0.08	0.03	0.10	0.99	0.77	1.11	0.48	0.01
Q68DK	Zinc finger FYVE domain-containing protein 26 OS=Homo sapiens GN=ZFYVE26 PE=1	1	0.4	1.21	0.71	1.78	1.89	0.03	-0.45	0.64	0.76	-0.03	-0.45	0.55	0.66	-0.35	-0.81	0.27	0.37	0.04
Q9H4L5	Oxysterol-binding protein-related protein 3 OS=Homo sapiens GN=OSBPL3 PE=1 SV=1	1	0.4	0.49	1.28	0.76	0.02	-0.01	0.80	0.30	-0.44	0.44	1.29	0.71	-0.04	0.11	0.93	0.43	-0.32	0.01
P08048	Zinc finger Y-chromosomal protein OS=Homo sapiens	1	0.4	0.83	0.36	0.09	0.17	1.64	1.18	0.93	1.02	0.67	0.27	-0.07	0.01	0.35	-0.09	-0.34	-0.28	0.01
P05121	Plasminogen activator inhibitor 1 OS=Homo sapiens GN=SERPINE1	4	0.4	-0.72	-0.23	0.13	-0.06	0.09	0.21	0.12	0.34	0.94	1.12	0.95	1.15	-0.02	0.36	1.08	1.24	0.01
O75882	Attractin OS=Homo sapiens GN=ATRN PE=1 SV=2 - [ATRN_HUMAN]	1	0.4	-0.19	0.21	-0.87	-0.31	1.39	1.81	0.76	1.32	0.64	1.11	-0.03	0.52	0.14	0.58	-0.48	0.07	0.04
P08246	Neutrophil elastase OS=Homo sapiens GN=ELANE PE=1 SV=1 -	2	0.4	0.70	0.17	-0.05	1.22	0.11	-0.17	-0.45	0.67	0.19	-0.02	-0.38	0.66	1.07	0.76	0.54	1.63	0.01
P00738	Haptoglobin OS=Homo sapiens GN=HP PE=1 SV=1 - [HPT_HUMAN]	19	0.4	0.42	0.75	0.99	0.83	-0.85	-0.50	-0.14	-0.26	-0.34	0.04	0.30	0.22	0.94	1.25	1.50	1.44	0.04

P08637	Low affinity immunoglobulin gamma Fc region receptor III-A OS=Homo sapiens	3	0.4	0.68	1.03	0.40	1.08	0.12	0.51	-0.07	0.62	0.47	0.92	0.26	0.93	-0.20	0.23	-0.49	0.11	0.00
P35232	Prohibitin OS=Homo sapiens GN=PHB PE=1 SV=1 - [PHB_HUMAN]	1	0.4	0.51	0.83	0.13	0.67	0.62	0.95	0.28	0.82	0.39	0.78	0.02	0.55	-0.06	0.29	-0.38	0.14	0.00
P01137	Transforming growth factor beta-1 OS=Homo sapiens GN=TGFB1	2	0.4	-0.13	0.45	-0.34	-0.33	0.47	1.07	0.30	0.15	0.53	1.17	0.32	0.59	0.50	1.11	0.34	0.31	0.00
Q6ULP	Aftiphilin OS=Homo sapiens GN=AFTPH PE=1 SV=2 -	1	0.4	-0.49	0.67	0.81	0.39	-1.15	0.03	0.19	-0.23	-0.30	0.93	1.01	0.58	0.15	1.34	1.50	1.06	0.04
P05164	Myeloperoxidase OS=Homo sapiens GN=MPO PE=1 SV=1 -	3	0.4	0.56	-0.03	0.75	-0.45	0.21	0.41	0.79	0.12	0.43	0.26	0.56	-0.13	1.05	0.18	1.87	-0.13	0.01
O0029	Tubby-related protein 1 OS=Homo sapiens GN=TULP1 PE=1 SV=3 -	1	0.4	1.41	0.70	-0.02	0.18	0.91	0.22	-0.48	-0.28	1.14	0.49	-0.29	-0.10	1.41	0.73	0.03	0.22	0.02
P02749	Beta-2-glycoprotein 1 OS=Homo sapiens GN=APOH PE=1 SV=3 -	23	0.4	0.27	0.82	0.59	0.03	0.71	1.22	1.04	0.48	0.73	1.27	1.03	0.51	-0.75	-0.28	-0.44	-0.99	0.04
Q86VS	IQ domain-containing protein H OS=Homo sapiens GN=IQCH PE=2	1	0.4	0.68	0.07	1.51	0.75	0.09	-0.50	0.96	0.21	0.00	-0.53	0.84	0.08	0.41	-0.17	1.29	0.52	0.02
O7590	Ras association domain-containing protein 9 OS=Homo sapiens GN=RASSF9 PE=2 SV=2	1	0.4	0.00	0.77	0.18	-0.04	0.88	1.67	1.10	0.88	-0.31	0.53	-0.13	-0.35	-0.01	0.79	0.22	-0.02	0.02
Q2TAC	Kinesin-like protein KIF19 OS=Homo sapiens GN=KIF19 PE=2 SV=2 -	2	0.4	0.28	-0.34	0.23	0.82	0.58	-0.02	0.57	1.17	0.49	-0.06	0.45	1.04	0.24	-0.35	0.24	0.82	0.00
P01712	Ig lambda chain V-II region WIN OS=Homo sapiens PE=1 SV=1 -	2	0.4	0.62	0.68	-0.08	0.39	0.78	1.12	0.42	0.54	0.63	0.78	0.05	0.39	0.07	0.35	-0.55	-0.05	0.00
P01623	Ig kappa chain V-III region WOL OS=Homo sapiens PE=1 SV=1 -	3	0.4	0.48	0.82	0.42	0.49	0.33	0.86	0.48	0.58	0.06	0.83	0.18	0.29	0.00	0.42	-0.23	0.05	0.00
P01781	Ig heavy chain V-III region GAL OS=Homo sapiens PE=1 SV=1 -	5	0.4	0.77	0.20	0.32	0.33	1.04	0.56	0.67	0.64	0.58	0.07	0.24	0.18	0.32	-0.08	0.09	0.08	0.00
Q5VVJ	Histone H2A deubiquitinase MYSM1 OS=Homo sapiens GN=MYSM1 PE=1 SV=1 -	1	0.4	0.17	0.40	0.52	-0.07	1.19	1.44	1.57	0.99	-0.35	-0.02	0.12	-0.77	0.08	0.34	0.48	-0.12	0.03
Q1476	Latent-transforming growth factor beta-binding protein 1 OS=Homo sapiens	5	0.4	0.34	0.54	0.24	-0.03	0.42	0.82	0.19	-0.15	0.23	0.61	0.15	-0.03	0.70	0.89	0.64	0.41	0.00
Q96JE	Microtubule-associated protein 6 OS=Homo sapiens GN=MAP6 PE=1	1	0.4	-0.48	0.14	0.62	-0.04	-0.25	0.39	0.89	0.23	-0.02	0.67	1.09	0.42	0.01	0.66	1.16	0.49	0.01
Q96L7	Histone-lysine N-methyltransferase, H3 lysine-36 and H4 lysine-20 specific OS=Homo sapiens GN=NSD1 PE=1	4	0.4	1.05	0.72	-0.70	-0.18	0.94	1.01	-0.37	0.24	0.78	1.40	-0.05	0.38	1.24	0.48	-0.72	-0.28	0.05
P19957	Elafin OS=Homo sapiens GN=PI3 PE=1 SV=3 -	1	0.4	0.11	-0.07	0.41	-0.02	0.84	0.68	1.17	0.75	0.31	0.20	0.61	0.18	0.16	0.01	0.51	0.06	0.00
P0277	Platelet factor 4 OS=Homo sapiens GN=PF4 PE=1 SV=2 -	3	0.4	0.39	1.47	-0.16	0.16	0.55	1.17	-0.20	-0.09	0.99	1.70	0.25	0.29	-0.18	0.62	-0.63	-0.45	0.05
P02549	Spectrin alpha chain, erythrocytic 1 OS=Homo sapiens GN=SPTA1	1	0.4	0.71	-0.14	-0.41	0.33	0.77	0.36	0.11	0.85	0.86	0.46	0.13	0.86	0.94	-0.46	0.04	0.42	0.01
Q1594	Zyxin OS=Homo sapiens GN=ZYX PE=1 SV=1 -	1	0.4	0.16	0.47	-0.15	-0.25	1.09	1.42	0.83	0.73	0.62	1.00	0.32	0.22	-0.11	0.23	-0.36	-0.47	0.02
Q4L23	Acyl-CoA synthetase family member 4 OS=Homo sapiens GN=AASDH PE=1 SV=3 -	1	0.4	0.72	0.27	1.08	-0.06	0.33	-0.09	0.73	-0.41	0.50	0.12	0.86	-0.29	0.68	0.26	1.08	-0.07	0.01

P01609	Ig kappa chain V-I region Scw OS=Homo sapiens PE=1 SV=1 -	3	0.4	0.56	1.17	0.14	0.63	0.46	0.42	0.11	0.67	0.07	0.40	-0.40	0.35	0.21	0.35	0.01	0.58	0.00
Q8TD8	Down syndrome cell adhesion molecule-like protein 1 OS=Homo sapiens GN=DSCAML1	1	0.4	-0.36	0.23	-0.17	-0.41	0.42	1.02	0.64	0.41	0.44	1.10	0.63	0.38	0.12	0.74	0.36	0.11	0.01
P01597	Ig kappa chain V-I region DEE OS=Homo sapiens PE=1 SV=1 -	3	0.4	0.48	0.41	0.63	0.90	0.08	0.26	0.28	0.54	0.14	-0.27	0.05	0.15	0.37	0.48	0.34	0.77	0.00
Q7Z7A	Centriolin OS=Homo sapiens GN=CNTRL PE=1 SV=2 -	2	0.3	0.13	0.71	0.39	0.73	0.08	0.61	0.38	0.45	0.03	0.62	0.36	0.30	-0.13	0.36	0.21	0.36	0.00
P07339	Cathepsin D OS=Homo sapiens GN=CTSD PE=1 SV=1 - [CATD_HUMAN]	2	0.3	0.22	0.40	1.13	0.67	-0.41	0.38	0.98	0.91	-1.08	-0.18	0.00	0.22	-0.37	0.48	1.27	0.94	0.05
P0CG0	Ig lambda-3 chain C regions OS=Homo sapiens GN=IGLC3 PE=1	2	0.3	-0.11	-0.07	1.06	-0.12	-0.07	0.42	1.16	0.30	-0.03	0.25	1.25	0.01	-0.05	0.16	0.88	0.47	0.01
9NWW	Uncharacterized protein C2orf42 OS=Homo sapiens GN=C2orf42	1	0.3	0.59	0.77	0.20	0.44	0.91	1.11	0.56	0.81	0.48	0.74	0.11	0.34	-0.33	-0.12	-0.66	-0.43	0.02
P02775	Platelet basic protein OS=Homo sapiens GN=PPBP PE=1 SV=3 -	8	0.3	0.28	1.01	0.07	0.02	0.19	0.86	0.06	0.03	0.47	1.20	0.23	0.27	0.10	0.88	-0.12	-0.08	0.01
P27816	Microtubule-associated protein 4 OS=Homo sapiens GN=MAP4 PE=1	1	0.3	0.93	0.41	1.00	1.09	-0.19	-0.69	-0.08	0.01	0.48	0.03	0.56	0.64	0.36	-0.13	0.48	0.56	0.01
P01620	Ig kappa chain V-III region SIE OS=Homo sapiens PE=1 SV=1 -	1	0.3	0.31	0.41	0.06	0.77	0.27	0.59	0.01	0.77	0.21	0.43	0.04	0.64	0.03	0.47	-0.21	0.65	0.00
P54108	Cysteine-rich secretory protein 3 OS=Homo sapiens GN=CRISP3	6	0.3	0.14	0.79	-0.28	-0.33	0.67	1.28	0.54	0.43	0.55	1.22	0.45	0.28	-0.16	0.48	-0.22	-0.42	0.02
O0015	PDZ and LIM domain protein 1 OS=Homo sapiens GN=PDLIM1	3	0.3	0.92	1.04	-0.17	0.78	0.30	0.69	-0.58	0.42	0.86	1.18	-0.02	0.72	-0.16	0.45	-0.98	-0.17	0.05
O6036	Putative nucleoside diphosphate kinase OS=Homo sapiens GN=NME2P1 PE=5 SV=1	1	0.3	0.40	0.30	-0.05	-0.21	0.88	0.79	0.46	0.31	0.73	0.70	0.29	0.12	0.40	0.33	0.00	-0.18	0.00
Q1310	Secreted phosphoprotein 24 OS=Homo sapiens GN=SPP2 PE=1 SV=1 -	3	0.3	0.55	0.00	-0.19	-0.04	0.92	0.46	0.09	0.29	0.72	0.27	0.19	0.15	0.72	0.33	0.47	0.30	0.00
P02787	Serotransferrin OS=Homo sapiens GN=TF PE=1 SV=3 -	55	0.3	0.33	0.42	0.24	0.04	0.59	0.67	0.49	0.33	0.43	0.64	0.35	0.18	0.17	0.26	0.12	-0.07	0.00
Q9NW3	E3 ubiquitin-protein ligase FANCL OS=Homo sapiens GN=FANCL	1	0.3	0.25	0.42	0.15	0.51	0.18	0.37	0.24	0.61	0.23	0.77	0.31	0.67	0.05	0.25	-0.11	0.24	0.00
P24158	Myeloblastin OS=Homo sapiens GN=PRTN3 PE=1 SV=3 -	2	0.3	0.50	0.64	-0.31	0.21	0.57	0.44	0.30	0.34	-0.04	0.03	0.23	0.01	0.64	0.87	0.18	0.44	0.00
Q9NSC	Alpha-N-acetylgalactosaminide alpha-2,6-sialyltransferase 1 OS=Homo sapiens	1	0.3	0.38	-0.06	0.23	-0.17	0.69	0.27	0.57	0.18	0.03	-0.34	-0.12	-0.52	1.22	0.81	1.12	0.71	0.03
P01624	Ig kappa chain V-III region POM OS=Homo sapiens PE=1 SV=1 -	2	0.3	0.29	0.84	0.72	0.73	-0.14	0.44	0.11	0.21	0.10	0.80	0.35	0.28	-0.42	0.29	0.02	0.34	0.00
P52294	Importin subunit alpha-5 OS=Homo sapiens GN=KPNA1 PE=1 SV=3 -	1	0.3	0.57	0.74	0.35	-0.16	0.54	0.72	0.35	-0.16	0.48	0.71	0.27	-0.25	0.36	0.55	0.19	-0.34	0.00
P01598	Ig kappa chain V-I region EU OS=Homo sapiens PE=1 SV=1 -	3	0.3	0.05	0.25	0.17	0.49	-0.10	0.03	0.14	0.37	0.75	1.21	0.80	1.26	-0.32	-0.20	-0.15	0.12	0.02
Q01459	Di-N-acetylchitobiase OS=Homo sapiens GN=CTBS PE=1 SV=1 -	2	0.3	-0.07	0.02	0.86	0.72	-0.43	-0.33	0.53	0.40	0.22	0.37	1.15	1.01	-0.38	-0.27	0.60	0.45	0.03

P0735	Platelet glycoprotein Ib alpha chain OS=Homo sapiens GN=GP1BA PE=1 SV=2 -	2	0.3	-0.41	0.49	0.01	0.27	0.20	1.12	0.66	0.93	-0.83	0.15	-0.40	-0.14	0.17	1.10	0.63	0.89	0.05
O7514	ICOS ligand OS=Homo sapiens GN=ICOSLG PE=1 SV=2 -	3	0.3	0.35	0.13	0.53	0.79	0.45	0.36	0.46	0.93	0.17	0.02	0.47	0.72	-0.39	-0.38	-0.20	0.34	0.01
P0395	Angiogenin OS=Homo sapiens GN=ANG PE=1 SV=1 - [ANGI_HUMAN]	3	0.3	0.44	0.27	0.31	1.62	-0.11	0.09	-0.58	0.18	0.23	0.57	-0.27	0.42	0.31	0.66	-0.05	0.59	0.03
P0185	Ig gamma-1 chain C region OS=Homo sapiens GN=IGHG1 PE=1 SV=1 -	8	0.3	0.19	0.79	0.20	0.15	0.23	1.01	0.26	0.29	0.21	0.90	0.18	0.25	-0.15	0.44	-0.19	-0.14	0.00
P2133	Filamin-A OS=Homo sapiens GN=FLNA PE=1 SV=4 - [FLNA_HUMAN]	1	0.3	0.43	0.50	0.27	0.57	0.03	0.12	-0.09	0.22	-0.33	-0.19	-0.48	-0.18	0.88	0.98	0.77	1.06	0.03
Q0855	Desmocollin-1 OS=Homo sapiens GN=DSC1 PE=1 SV=2 - [DSC1_HUMAN]	3	0.3	0.07	0.23	0.23	0.01	0.37	0.49	0.48	0.27	0.51	0.72	0.67	0.44	0.08	-0.03	0.07	-0.06	0.00
P0276	Serum albumin OS=Homo sapiens GN=ALB PE=1 SV=2 -	74	0.3	0.27	0.40	0.49	0.31	0.14	0.30	0.39	0.22	0.05	0.25	0.25	0.13	0.18	0.37	0.45	0.29	0.00
Q17RP	Tigger transposable element-derived protein 6 OS=Homo sapiens GN=TIGD6 PE=2 SV=2 -	1	0.3	0.99	0.01	0.53	0.74	0.67	-0.29	0.26	0.47	0.46	-0.45	0.01	0.21	0.61	-0.34	0.21	0.40	0.02
9BWT	Poly(A) polymerase gamma OS=Homo sapiens GN=PAPOLG	1	0.3	0.79	0.84	0.60	0.50	-0.28	-0.20	-0.43	-0.52	0.19	0.32	0.01	-0.09	0.76	0.85	0.62	0.51	0.03
P0170	Ig lambda chain V-I region NEWM OS=Homo sapiens PE=1 SV=1 -	3	0.3	0.79	0.88	0.46	-0.35	0.67	0.35	0.06	-0.44	0.39	0.58	0.02	-0.31	0.76	0.55	0.19	-0.14	0.02
9H0W	Protein SMG9 OS=Homo sapiens GN=SMG9 PE=1 SV=1 - [SMG9_HUMAN]	1	0.3	0.53	0.59	0.52	0.28	0.79	0.56	0.82	0.59	-0.27	-0.14	-0.27	-0.52	0.36	0.01	0.40	0.15	0.02
6NM6	Leucine-rich repeat-containing protein 53 OS=Homo sapiens GN=LRRC53 PE=4 SV=2	1	0.3	0.13	-0.08	0.01	0.38	0.68	0.49	0.60	0.97	0.20	0.07	0.10	0.46	0.07	-0.11	0.01	0.36	0.00
O1521	WD repeat-containing protein 46 OS=Homo sapiens GN=WDR46	1	0.3	0.46	0.15	0.68	0.49	0.24	-0.06	0.50	0.31	-0.41	-0.65	-0.18	-0.38	0.78	0.49	1.05	0.85	0.04
P5966	Neutrophil defensin 1 OS=Homo sapiens GN=DEFA1 PE=1 SV=1 -	5	0.3	0.57	0.78	-0.32	-0.01	0.62	0.92	-0.24	0.15	0.50	0.75	-0.44	-0.02	0.44	0.78	-0.31	0.07	0.03
P0948	SPARC OS=Homo sapiens GN=SPARC PE=1 SV=1 -	11	0.3	-0.04	0.58	-0.11	0.06	0.35	0.96	0.35	0.54	0.05	0.66	-0.03	0.10	0.02	0.69	-0.04	0.08	0.01
P68871	Hemoglobin subunit beta OS=Homo sapiens GN=HBB PE=1 SV=2 -	9	0.3	0.70	-0.29	0.59	0.71	0.61	-0.50	0.37	0.54	0.46	-0.51	0.30	0.51	0.42	-0.53	0.33	0.47	0.03
O7544	Alpha-tectorin OS=Homo sapiens GN=TECTA PE=1 SV=3 -	1	0.3	-0.16	0.70	0.00	0.27	0.42	1.29	0.61	0.89	-0.39	0.54	-0.22	0.04	-0.35	0.54	-0.14	0.12	0.05
8NCM	Cytoplasmic dynein 2 heavy chain 1 OS=Homo sapiens GN=DYNC2H1	2	0.3	0.26	0.52	0.58	0.63	0.05	0.33	0.41	0.46	0.11	-0.13	0.35	-0.27	0.12	0.13	0.45	0.15	0.00
P0443	Ig kappa chain V-III region VG (Fragment) OS=Homo sapiens PE=1	2	0.3	0.21	0.40	0.21	0.35	0.31	0.60	0.35	0.50	0.20	0.43	0.13	0.30	-0.03	0.13	-0.08	0.12	0.00
Q9Y4F	Protein FAM179B OS=Homo sapiens GN=FAM179B PE=1	2	0.3	0.09	0.65	0.97	-0.01	-0.18	0.39	0.73	-0.24	0.07	0.69	0.95	-0.03	-0.35	0.24	0.58	-0.42	0.04
Q86XR	Centrosomal protein of 57 kDa OS=Homo sapiens GN=CEP57 PE=1	1	0.3	0.03	-0.42	0.03	-0.03	0.89	0.46	0.93	0.87	0.47	0.10	0.49	0.41	0.06	-0.35	0.12	0.04	0.02
P01601	Ig kappa chain V-I region HK101 (Fragment) OS=Homo sapiens PE=4	2	0.3	0.50	0.86	0.66	0.33	0.11	0.82	0.33	0.15	-0.19	0.52	0.08	-0.20	-0.10	0.41	-0.10	-0.14	0.01
9H1M	Nucleoporin-62 C-terminal-like protein OS=Homo sapiens GN=NUP62CL PE=2	1	0.3	-0.04	-0.11	0.31	-0.06	0.65	0.60	1.03	0.67	-0.11	-0.11	0.25	-0.13	0.18	0.14	0.58	0.19	0.01

Q9282(	Gamma-glutamyl hydrolase OS=Homo sapiens GN=GGH PE=1	3	0.3	0.54	0.38	0.75	0.74	-0.27	-0.37	0.01	0.11	-0.36	-0.30	0.03	-0.03	0.68	0.30	0.88	0.91	0.05
)8WU6	Thrombospondin-type laminin G domain and EAR repeat-containing protein OS=Homo sapiens GN=TSPEAR	1	-0.3	-0.27	-0.25	-0.61	-0.81	-0.16	-0.11	-0.46	-0.65	0.53	0.62	0.19	-0.01	-0.34	-0.28	-0.63	-0.84	0.03
Q969G:	LIM/homeobox protein Lhx4 OS=Homo sapiens GN=LHX4 PE=1 SV=2 -	1	-0.3	-0.42	-0.16	-0.38	-0.19	-0.20	0.08	-0.12	0.07	-0.52	-0.19	-0.47	-0.29	-0.51	-0.22	-0.41	-0.24	0.00
Q9H21:	DNA replication factor Cdt1 OS=Homo sapiens GN=CDT1 PE=1 SV=3 -	1	-0.3	-0.16	0.27	0.14	-0.53	-0.35	0.09	-0.02	-0.68	-0.35	0.15	-0.05	-0.72	-0.64	-0.18	-0.29	-0.97	0.01
P50851	Lipopolysaccharide-responsive and beige-like anchor protein OS=Homo sapiens GN=LRBA PE=1	1	-0.3	-0.46	-0.09	-0.53	-0.71	-0.69	-0.30	-0.73	-0.89	0.19	0.63	0.12	-0.06	-0.25	0.15	-0.27	-0.46	0.02
Q9NT6	Teneurin-2 OS=Homo sapiens GN=TENM2 PE=1 SV=3 -	1	-0.3	0.34	0.01	-0.16	0.11	0.13	-0.18	-0.33	-0.06	-0.36	-0.62	-0.85	-0.59	-0.23	-0.53	-0.68	-0.43	0.00
P35503	UDP-glucuronosyltransferase 1-3 OS=Homo sapiens	1	-0.3	-0.34	0.63	0.17	-0.07	-0.98	0.01	-0.43	-0.67	-1.06	-0.02	-0.54	-0.79	-0.56	0.44	0.01	-0.25	0.04
Q9Y3X(	Coiled-coil domain-containing protein 9 OS=Homo sapiens GN=CCDC9 PE=1 SV=1 -	1	-0.3	-0.62	-0.31	-0.04	-0.24	-0.67	-0.35	-0.06	-0.25	-0.66	-0.28	-0.08	-0.28	-0.52	-0.19	0.10	-0.11	0.00
A2RU4	Single-pass membrane and coiled-coil domain-containing protein 3 OS=Homo sapiens	1	-0.3	0.21	0.47	-0.36	0.24	-0.26	0.02	-0.79	-0.18	-0.48	0.43	-0.45	-0.44	-0.72	-0.42	-1.23	-0.65	0.03
Q9BXX	Ankyrin repeat domain-containing protein 30A OS=Homo sapiens GN=ANKRD30A PE=1	1	-0.3	-1.24	-0.99	-0.86	-0.85	-0.37	-0.10	0.05	0.06	0.08	0.40	0.47	0.47	-0.73	-0.45	-0.30	-0.31	0.05
Q1401!	Coactosin-like protein OS=Homo sapiens GN=COTL1 PE=1 SV=3 -	1	-0.3	-0.36	0.22	-0.87	-0.51	-0.05	0.20	-0.73	-0.16	0.11	0.42	-0.59	-0.04	-0.56	-0.05	-1.21	-0.53	0.02
P01742	Ig heavy chain V-I region EU OS=Homo sapiens PE=1 SV=1 -	1	-0.3	-0.27	-0.57	-0.86	-0.84	0.52	-0.17	-0.28	-0.33	0.46	0.49	-0.31	-0.20	-0.33	-0.50	-0.96	-0.73	0.02
P07093	Glia-derived nexin OS=Homo sapiens GN=SERPINE2 PE=1	2	-0.3	-0.17	0.19	-0.62	-0.60	0.08	0.46	-0.34	-0.32	-0.40	0.03	-0.85	-0.84	-0.32	0.08	-0.72	-0.71	0.01
P78527	DNA-dependent protein kinase catalytic subunit OS=Homo sapiens GN=PRKDC PE=1 SV=3 -	1	-0.3	0.35	-0.99	-0.60	0.23	0.36	-0.69	-0.29	0.55	-0.05	-1.11	-0.79	0.04	0.03	-1.24	-0.83	-0.01	0.04
P01771	Ig heavy chain V-III region HIL OS=Homo sapiens PE=1 SV=1 -	1	-0.3	0.09	-1.05	-0.42	-0.11	-0.20	-1.31	-0.66	-0.35	0.56	-0.51	0.06	0.36	0.05	-1.06	-0.40	-0.11	0.03
P24387	Corticotropin-releasing factor-binding protein OS=Homo sapiens GN=CRHBP PE=1 SV=2 -	3	-0.3	0.21	0.09	-0.09	0.18	-0.73	-0.89	-0.99	-0.42	-0.35	-0.67	-0.94	-0.25	0.01	-0.14	-0.23	0.14	0.01
P01604	Ig kappa chain V-I region Kue OS=Homo sapiens PE=1 SV=1 -	2	-0.3	-0.51	-0.02	0.64	0.54	-1.14	-0.64	0.04	-0.08	-1.21	-0.77	-0.09	-0.26	-1.05	-0.59	0.09	-0.05	0.04
O1479:	Apolipoprotein L1 OS=Homo sapiens GN=APOL1 PE=1 SV=5 -	16	-0.3	-0.31	0.17	-0.62	-0.84	-0.43	-0.04	-0.74	-0.99	-0.02	0.43	-0.34	-0.34	0.04	0.29	-0.60	-0.78	0.01
P2081(	Calpastatin OS=Homo sapiens GN=CAST PE=1 SV=4 - [ICAL_HUMAN]	1	-0.3	0.25	0.15	-0.49	-0.02	0.04	0.23	-0.39	0.08	-0.59	-0.34	-1.04	-0.58	-0.60	-0.39	-1.01	-0.56	0.01
P49137	MAP kinase-activated protein kinase 2 OS=Homo sapiens GN=MAPKAPK2 PE=1	2	-0.3	-0.38	-0.62	-0.67	-0.36	-0.33	-0.54	-0.58	-0.26	-0.45	-0.61	-0.74	-0.43	0.26	0.05	0.02	0.32	0.00
P12111	Collagen alpha-3(VI) chain OS=Homo sapiens GN=COL6A3 PE=1 SV=5	5	-0.3	0.12	-0.50	-0.21	-0.53	0.08	-0.63	-0.37	-0.59	0.37	-0.33	0.04	-0.28	-0.34	-0.86	-0.51	-0.79	0.00

P00740	Coagulation factor IX OS=Homo sapiens GN=F9 PE=1 SV=2 -	9	-0.3	-0.66	-0.93	-0.61	-0.60	-0.29	-0.52	-0.26	-0.13	-0.31	-0.52	-0.42	-0.34	0.27	-0.23	-0.03	0.21	0.00
Q1541	Ryanodine receptor 3 OS=Homo sapiens GN=RYR3 PE=1 SV=3 -	1	-0.3	-0.79	-0.99	-0.07	-0.46	-0.83	-1.02	-0.08	-0.46	-0.44	-0.58	0.28	-0.11	-0.20	-0.37	0.57	0.16	0.01
P52630	Signal transducer and activator of transcription 2 OS=Homo sapiens GN=STAT2 PE=1 SV=1 -	1	-0.3	-0.34	-1.39	-0.79	-0.78	-0.06	-1.09	-0.47	-0.46	0.48	-0.50	0.04	0.04	0.43	-0.58	0.04	0.04	0.02
P04406	Glyceraldehyde-3-phosphate dehydrogenase OS=Homo sapiens	3	-0.3	-0.23	-0.80	-0.22	0.19	-0.27	-1.03	-0.68	0.05	-0.43	-1.42	-0.79	-0.35	0.12	-0.40	0.15	0.72	0.02
O94830	Phospholipase DDHD2 OS=Homo sapiens GN=DDHD2 PE=1 SV=2 -	1	-0.3	-0.35	-0.12	-0.38	-0.67	0.12	0.37	0.12	-0.16	-0.78	-0.47	-0.80	-1.10	-0.31	-0.05	-0.29	-0.59	0.00
O0018	Mannan-binding lectin serine protease 2 OS=Homo sapiens GN=MASP2 PE=1 SV=4 -	8	-0.3	0.19	-0.06	-0.28	-0.76	0.21	-0.36	-0.35	-0.70	0.15	-0.21	-0.30	-0.81	-0.13	-0.53	-0.54	-1.01	0.00
P0C09	FRAS1-related extracellular matrix protein 3 OS=Homo sapiens GN=FREM3	1	-0.4	0.50	0.01	-0.40	-0.10	0.26	-0.21	-0.60	-0.30	-0.32	-0.73	-1.21	-0.91	0.06	-0.39	-0.78	-0.50	0.01
P07911	Uromodulin OS=Homo sapiens GN=UMOD PE=1 SV=1 -	1	-0.4	-0.22	0.31	-0.14	-0.38	-0.69	-0.11	-0.60	-0.83	-0.89	-0.26	-0.86	-1.16	-0.04	0.46	0.04	-0.25	0.01
Q8N6H	ADP-ribosylation factor GTPase-activating protein 2 OS=Homo sapiens GN=ARFGAP2	1	-0.4	0.04	-0.33	0.07	0.07	-0.31	-0.65	-0.24	-0.22	-0.54	-0.84	-0.51	-0.51	-0.38	-0.71	-0.29	-0.30	0.00
P12259	Coagulation factor V OS=Homo sapiens GN=F5 PE=1 SV=4 -	31	-0.4	0.15	0.09	-0.35	-0.09	-0.22	-0.31	-0.86	-0.49	-0.19	-0.39	-0.95	-0.56	-0.22	-0.19	-0.67	-0.44	0.00
Q9HCR	Dual 3',5'-cyclic-AMP and -GMP phosphodiesterase 11A OS=Homo sapiens GN=PDE11A PE=1 SV=2	2	-0.4	-0.09	-0.02	-0.15	-0.34	-0.46	-0.37	-0.48	-0.67	-0.43	-0.29	-0.48	-0.68	-0.28	-0.18	-0.29	-0.49	0.00
P55895	V(D)J recombination-activating protein 2 OS=Homo sapiens GN=RAG2 PE=1 SV=1 -	1	-0.4	-0.70	-0.08	-0.60	-0.65	-0.38	0.26	-0.20	-0.18	-0.22	0.29	-0.31	-0.34	-0.68	-0.30	-0.89	-0.75	0.00
Q9NXF	Testis-expressed sequence 10 protein OS=Homo sapiens GN=TEX10 PE=1 SV=2 -	1	-0.4	-0.52	-0.43	-0.58	-0.79	0.03	0.13	-0.16	-0.41	-0.33	-0.17	-0.38	-0.59	-0.34	-0.23	-0.38	-0.58	0.00
Q9GZV	Ankyrin repeat domain-containing protein 2 OS=Homo sapiens GN=ANKRD2 PE=1	1	-0.4	0.00	-0.06	-0.54	-0.65	-0.26	-0.30	-0.76	-0.87	0.06	0.07	-0.47	-0.59	-0.07	-0.10	-0.56	-0.69	0.00
Q08828	Adenylate cyclase type 1 OS=Homo sapiens GN=ADCY1 PE=1 SV=2 -	1	-0.4	0.05	0.28	-0.16	-0.25	-0.03	0.22	-0.20	-0.29	-0.74	-0.44	-0.94	-1.04	-0.53	-0.27	-0.68	-0.79	0.00
Q9BX9	Group XIIB secretory phospholipase A2-like protein OS=Homo sapiens GN=PLA2G12B	1	-0.4	-0.21	-0.58	-0.51	-1.36	-0.08	-0.42	-0.34	-1.18	-0.18	-0.48	-0.48	-1.33	0.75	0.41	0.50	-0.36	0.03
O1483	Phytanoyl-CoA dioxygenase, peroxisomal OS=Homo sapiens GN=PHYH PE=1	1	-0.4	-1.05	0.21	0.34	0.09	-1.07	0.21	0.35	0.11	-1.51	-0.18	-0.12	-0.38	-1.70	-0.41	-0.27	-0.53	0.04
Q9BWP	Collectin-11 OS=Homo sapiens GN=COLEC11 PE=1 SV=1 -	1	-0.4	-0.58	-0.74	-0.12	0.03	-1.21	-1.35	-0.71	-0.56	-0.68	-0.77	-0.21	-0.07	0.00	-0.13	0.52	0.65	0.02
Q6E0U	Dermokine OS=Homo sapiens GN=DMKN PE=1 SV=3 -	2	-0.4	-0.72	0.05	-0.95	-0.04	-0.48	0.31	-0.66	0.25	-1.05	-0.20	-1.26	-0.36	-0.57	0.24	-0.74	0.16	0.01
Q6ZRI	Rho GTPase-activating protein 36 OS=Homo sapiens GN=ARHGAP36	2	-0.4	0.62	-1.01	-1.03	-0.58	1.05	-0.56	-0.56	-0.11	0.52	-1.04	-1.12	-0.68	0.69	-0.91	-0.91	-0.47	0.05

Q6ZV7	FYVE, RhoGEF and PH domain-containing protein 6 OS=Homo sapiens GN=FGD6 PE=1 SV=2 -	1	-0.4	0.10	0.29	-0.30	-0.26	-0.57	-0.36	-0.94	-0.89	-0.85	-0.59	-1.24	-1.21	0.29	0.52	-0.06	-0.03	0.01
Q9NUA	Zinc finger and BTB domain-containing protein 40 OS=Homo sapiens GN=ZBTB40 PE=1 SV=4	2	-0.4	0.07	0.43	0.71	-0.06	-0.71	-0.33	-0.04	-0.80	-1.02	-0.58	-0.37	-1.15	-0.82	-0.42	-0.13	-0.92	0.01
P0400	Vitronectin OS=Homo sapiens GN=VTN PE=1 SV=1 - [VTNC_HUMAN]	17	-0.4	-0.48	-0.58	-0.01	-0.01	-1.10	-0.99	-0.56	-0.51	-1.02	-1.06	-0.33	-0.29	0.27	-0.05	0.23	0.32	0.01
Q9UNF	Melanoma-associated antigen D2 OS=Homo sapiens GN=MAGED2	1	-0.4	-0.98	0.23	0.23	-0.22	-1.21	0.02	0.05	-0.41	-1.57	-0.29	-0.34	-0.81	-1.08	0.16	0.18	-0.29	0.01
Q96FZ	Charged multivesicular body protein 6 OS=Homo sapiens GN=CHMP6 PE=1 SV=3 -	1	-0.4	-0.68	-0.53	-0.36	0.19	-0.23	-0.06	0.12	0.68	-1.09	-0.87	-0.77	-0.22	-1.03	-0.85	-0.66	-0.13	0.00
O6060	Toll-like receptor 5 OS=Homo sapiens GN=TLR5 PE=1 SV=4 -	1	-0.4	-0.40	-0.35	-0.99	-0.97	0.33	0.39	-0.22	-0.20	-0.36	-0.25	-0.94	-0.93	-0.16	-0.08	-0.69	-0.69	0.00
Q8IVI	Nostrin OS=Homo sapiens GN=NOSTRIN PE=1 SV=2 -	1	-0.4	-0.22	-0.31	-0.16	0.49	-0.86	-0.94	-0.76	-0.11	-0.58	-0.61	-0.52	0.13	-0.74	-0.80	-0.63	0.00	0.00
P48547	Potassium voltage-gated channel subfamily C member 1 OS=Homo sapiens GN=KCNC1	1	-0.4	0.00	-0.91	-0.16	0.08	-0.32	-1.22	-0.44	-0.19	-0.91	-1.76	-1.07	-0.83	0.50	-0.39	0.39	0.61	0.03
Q96KV	WD repeat-containing protein 90 OS=Homo sapiens GN=WDR90	1	-0.4	-0.28	0.42	1.34	0.01	-1.61	-0.89	0.05	-1.15	-1.23	-0.45	0.40	-0.81	-1.32	-0.59	0.35	-0.87	0.05
Q7RTP	Protein-methionine sulfoxide oxidase MICAL3 OS=Homo sapiens GN=MICAL3	1	-0.4	-0.73	-0.54	0.72	-0.28	-1.78	-1.58	-0.29	-1.28	-1.12	-0.87	0.34	-0.67	-0.25	-0.03	1.25	0.24	0.05
P06331	Ig heavy chain V-II region ARH-77 OS=Homo sapiens PE=4	2	-0.4	-0.40	-0.45	-0.41	-0.66	-0.28	0.03	0.03	-0.13	-0.89	-0.78	-0.80	-1.06	-0.29	-0.29	-0.10	-0.59	0.00
Q96P4	Arf-GAP with Rho-GAP domain, ANK repeat and PH domain-containing protein 1 OS=Homo sapiens GN=ARAP1	1	-0.4	0.02	0.69	-0.25	-0.78	-1.27	-0.61	-1.35	-1.90	-0.10	0.47	-0.36	-0.90	-0.06	0.41	-0.34	-0.82	0.02
Q9NZJ	Sacsin OS=Homo sapiens GN=SACS PE=1	3	-0.4	-0.62	0.09	-0.99	-0.51	-0.09	0.65	-0.41	0.07	-1.04	-0.26	-1.40	-0.93	-0.58	0.16	-0.90	-0.43	0.00
P22891	Vitamin K-dependent protein Z OS=Homo sapiens GN=PROZ PE=1	3	-0.4	-0.55	-0.10	-0.27	-0.26	-0.21	0.43	-0.16	-0.23	-0.73	-0.07	-1.13	-1.70	-0.86	-0.32	-0.43	-0.60	0.00
Q9Y6D	Brefeldin A-inhibited guanine nucleotide-exchange protein 1 OS=Homo sapiens	2	-0.5	-0.65	-2.01	-0.49	-0.54	-0.27	-1.61	-0.07	-0.12	-0.43	-1.71	-0.25	-0.31	0.54	-0.78	0.76	0.70	0.04
P0C0L	Complement C4-B OS=Homo sapiens GN=C4B PE=1 SV=2 -	4	-0.5	-0.78	-1.23	0.31	-0.60	-1.18	-1.55	0.24	-0.78	-1.13	-1.63	0.33	-0.87	0.19	-0.45	1.42	0.36	0.05
Q96GM	Target of EGR1 protein 1 OS=Homo sapiens GN=TOE1 PE=1 SV=1 -	1	-0.5	-0.73	-0.11	-0.22	-0.70	-0.97	-0.33	-0.42	-0.90	-0.37	0.33	0.15	-0.33	-1.04	-0.38	-0.48	-0.97	0.00
A2VDJ	Transmembrane protein 131-like OS=Homo sapiens GN=KIAA0922	1	-0.5	0.05	-0.22	0.11	-0.13	-0.79	-1.03	-0.69	-0.92	-0.59	-0.78	-0.52	-0.76	-0.27	-0.50	-0.16	-0.41	0.00
Q96ER	Coiled-coil domain-containing protein 51 OS=Homo sapiens GN=CCDC51 PE=1	1	-0.5	-1.32	-0.93	-1.19	-1.04	-0.52	-0.11	-0.35	-0.20	-0.51	-0.05	-0.37	-0.23	-0.45	-0.02	-0.26	-0.13	0.00
Q96S9	Phosphatidylethanolamine-binding protein 4 OS=Homo sapiens GN=PEBP4 PE=1 SV=3 -	6	-0.5	-0.64	-1.08	-0.92	0.01	-0.42	-1.20	-0.53	0.19	-0.76	-1.30	-0.68	0.00	-0.03	-0.37	-0.30	0.34	0.00
P0159	Ig kappa chain V-I region Gal OS=Homo sapiens PE=1 SV=1 -	1	-0.5	0.83	-0.34	0.08	0.08	0.44	-0.71	-0.27	-0.26	-0.98	-2.08	-1.73	-1.73	0.37	-0.77	-0.33	-0.34	0.03

P01596	Ig kappa chain V-I region CAR OS=Homo sapiens PE=1 SV=1 -	3	-0.5	-0.45	-0.29	-0.30	0.45	-1.01	-0.83	-0.82	-0.06	-0.79	-0.56	-0.62	0.12	-1.02	-0.82	-0.81	-0.07	0.00
Q5MNZ	WD repeat domain phosphoinositide-interacting protein 1 OS=Homo sapiens GN=WIPI1 PE=1 SV=3 -	2	-0.5	0.47	-0.29	-0.67	-1.31	0.54	-0.71	-0.60	-1.24	-0.02	-1.22	-1.20	-1.85	1.02	0.30	-0.24	-0.90	0.03
Q8WX9	Negative elongation factor B OS=Homo sapiens GN=NELFB	1	-0.5	0.01	-0.01	-1.06	-0.80	-0.26	-0.35	-1.37	-0.68	0.54	0.13	-0.97	-0.09	-0.35	-0.34	-1.36	-1.11	0.00
O1492	Histone acetyltransferase type B catalytic subunit OS=Homo sapiens	1	-0.5	0.16	0.04	0.32	0.11	-0.82	-0.92	-0.63	-0.82	-0.51	-0.56	-0.35	-0.56	-0.91	-1.00	-0.70	-0.92	0.00
Q9275	Nuclear receptor ROR-beta OS=Homo sapiens GN=RORB PE=1 SV=3 -	1	-0.5	-0.03	-0.59	-0.77	-0.23	0.24	-0.30	-0.46	0.09	0.00	-0.49	-0.73	-0.20	-0.83	-1.35	-1.51	-0.98	0.00
Q5W0B	Transmembrane protein 236 OS=Homo sapiens GN=TMEM236 PE=2	1	-0.5	-0.31	-0.14	-0.70	-0.58	0.36	0.55	0.00	0.13	-0.76	-0.52	-1.15	-1.04	-0.90	-0.70	-1.24	-1.14	0.00
Q8NA4	Coiled-coil domain-containing protein 63 OS=Homo sapiens GN=CCDC63 PE=2	1	-0.5	-0.92	-1.30	-1.29	-0.13	-0.01	-0.64	-0.61	0.55	0.12	-1.35	-1.41	-0.26	0.14	-0.76	-0.73	0.41	0.01
O6050	Nephrin OS=Homo sapiens GN=NPHS1 PE=1 SV=1 -	1	-0.5	-0.58	-1.22	-1.59	-0.76	-0.12	-0.74	-1.09	-0.25	0.41	-0.15	-0.59	0.24	-0.02	-0.63	-0.98	-0.16	0.00
Q8IXS	Coiled-coil domain-containing protein 65 OS=Homo sapiens GN=CCDC65 PE=1	1	-0.5	-0.45	-0.32	-0.42	-1.03	-0.48	-0.32	-0.41	-1.01	-0.38	-0.17	-0.35	-0.96	-0.41	-0.24	-0.33	-0.95	0.00
Q9NQ8	Uncharacterized protein C12orf4 OS=Homo sapiens GN=C12orf4	1	-0.5	-0.56	-0.49	-0.40	-0.29	-0.63	-0.53	-0.42	-0.32	-0.75	-0.61	-0.58	-0.48	-0.71	-0.61	-0.49	-0.41	0.00
Q6Y7W	PERQ amino acid-rich with GYF domain-containing protein 2 OS=Homo sapiens	1	-0.5	-0.54	-1.10	0.79	0.01	-1.35	-1.95	-0.18	-0.78	-1.24	-1.74	-0.26	-0.64	-0.21	-0.77	0.96	0.67	0.03
P05165	Propionyl-CoA carboxylase alpha chain, mitochondrial OS=Homo sapiens GN=PCCA PE=1	1	-0.5	-0.04	0.24	-0.02	0.38	-0.01	0.45	0.21	0.45	-2.10	-1.83	-2.15	-2.31	-0.65	-0.27	-0.51	-0.19	0.05
Q6IA8	Elongator complex protein 2 OS=Homo sapiens GN=ELP2 PE=1	1	-0.5	-0.57	-2.07	-1.09	-0.47	0.05	-1.43	-0.44	0.19	0.50	-0.93	-0.02	0.59	-0.21	-1.68	-0.68	-0.08	0.02
Q9BV7	Centrosome-associated protein CEP250 OS=Homo sapiens GN=CEP250 PE=1 SV=2 -	1	-0.5	0.35	-0.56	0.58	1.47	-1.21	-2.10	-0.95	-0.05	-0.92	-1.76	-0.69	0.19	-0.82	-1.69	-0.54	0.34	0.05
P02741	C-reactive protein OS=Homo sapiens GN=CRP PE=1 SV=1 -	8	-0.5	-0.38	0.24	-0.38	-1.11	-0.78	-0.03	-0.78	-1.52	0.27	1.07	0.19	-0.48	-1.26	-0.50	-1.15	-1.89	0.01
P33176	Kinesin-1 heavy chain OS=Homo sapiens GN=KIF5B PE=1 SV=1 -	1	-0.5	0.26	-0.14	-0.12	-0.63	0.33	-1.44	-0.03	-0.51	-0.19	-2.03	-0.70	-1.07	-0.64	-0.76	0.65	-1.49	0.01
P47895	Aldehyde dehydrogenase family 1 member A3 OS=Homo sapiens GN=ALDH1A3 PE=1	1	-0.5	0.28	-1.66	-0.89	-0.92	0.79	-1.13	-0.34	-0.37	0.76	-1.11	-0.41	-0.45	0.25	-1.66	-0.87	-0.92	0.01
Q1499	Proteasome activator complex subunit 4 OS=Homo sapiens GN=PSME4 PE=1 SV=2 -	1	-0.5	-0.73	-1.29	-1.52	-1.60	0.52	-0.03	-0.24	-0.31	0.61	0.12	-0.17	-0.26	-0.41	-0.94	-1.15	-1.24	0.01
Q9GZP	Platelet-derived growth factor D OS=Homo sapiens GN=PDGFD	1	-0.5	-0.50	-0.46	-0.68	-0.33	-0.97	-0.92	-1.12	-0.76	-0.48	-0.38	-0.66	-0.31	-0.33	-0.26	-0.46	-0.12	0.00
Q86YJ	Ankyrin repeat domain-containing protein 13B OS=Homo sapiens GN=ANKRD13B PE=1	1	-0.5	-0.19	-0.31	-1.57	-0.69	-0.22	-0.32	-1.56	-0.67	-0.73	-0.78	-2.10	-1.23	0.86	0.78	-0.46	0.40	0.02

.5D8W	Cilia- and flagella-associated protein 69 OS=Homo sapiens GN=CFAP69 PE=2 SV=3	1	-0.5	-0.53	-0.33	-1.45	-0.47	-0.17	0.05	-1.05	-0.06	-0.49	-0.22	-1.40	-0.42	-0.43	-0.20	-1.30	-0.33	0.00
Q96RT	Gamma-tubulin complex component 6 OS=Homo sapiens GN=TUBGCP6 PE=1 SV=3 -	1	-0.6	0.09	-1.14	-0.78	-1.32	0.14	-1.07	-0.70	-1.23	0.72	-0.43	-0.15	-0.69	0.27	-0.93	-0.55	-1.10	0.00
Q5SYC	Clavesin-2 OS=Homo sapiens GN=CLVS2 PE=2 SV=1 -	1	-0.6	-0.55	-0.65	-1.42	-2.26	0.70	0.62	-0.13	-0.97	0.37	0.35	-0.49	-1.34	-0.17	-0.23	-0.98	-1.84	0.02
Q1467	KN motif and ankyrin repeat domain-containing protein 1 OS=Homo sapiens GN=KANK1	1	-0.6	-0.90	-0.12	-0.25	-0.29	-1.01	-0.40	-0.51	-0.59	-0.79	-0.26	-0.28	-0.51	-1.10	-0.54	-0.61	-0.86	0.00
P49221	Protein-glutamine gamma-glutamyltransferase 4 OS=Homo sapiens GN=TGM4 PE=1 SV=2 -	2	-0.6	0.12	0.23	-0.07	-0.88	-0.24	-0.11	-0.39	-1.20	-0.66	-0.48	-0.85	-1.67	-0.47	-0.33	-0.61	-1.44	0.00
Q9Y5Q	General transcription factor 3C polypeptide 3 OS=Homo sapiens GN=GTF3C3 PE=1 SV=1	1	-0.6	-0.63	0.49	0.57	-1.64	-1.13	0.01	0.11	-2.09	-0.46	0.73	0.75	-1.46	-1.44	-0.29	-0.18	-2.40	0.04
P1122	Mannose-binding protein C OS=Homo sapiens GN=MBL2 PE=1 SV=2 -	4	-0.6	-0.09	-0.70	-0.01	0.59	-0.65	-1.45	-0.84	-0.12	-0.60	-1.16	-0.65	0.04	-0.93	-1.44	-0.87	-0.20	0.00
P0188	Ig delta chain C region OS=Homo sapiens GN=IGHD PE=1 SV=2 -	5	-0.6	0.52	-1.52	-0.24	0.02	-0.64	-2.59	-1.30	-1.07	-0.34	-2.12	-0.81	-0.84	1.07	-0.58	0.51	0.84	0.04
Q8N20	Integrator complex subunit 1 OS=Homo sapiens GN=INTS1 PE=1	1	-0.6	-0.28	0.22	0.01	0.10	-1.23	-0.71	-0.90	-0.81	-0.78	-0.21	-0.49	-0.40	-1.23	-0.70	-0.89	-0.81	0.00
P53634	Dipeptidyl peptidase 1 OS=Homo sapiens GN=CTSC PE=1 SV=2 -	1	-0.6	-0.38	0.05	-0.33	-0.85	-0.33	0.12	-0.24	-0.75	-1.04	-0.54	-0.98	-1.50	-0.63	-0.16	-0.52	-1.05	0.00
Q9UL1	Sarcosine dehydrogenase, mitochondrial OS=Homo sapiens GN=SARDH	1	-0.6	-0.88	0.26	-1.01	-1.88	-1.20	-0.04	-1.29	-2.16	0.26	1.47	0.14	-0.74	-0.58	0.60	-0.65	-1.54	0.03
Q9BUN	Protein MENT OS=Homo sapiens GN=MENT PE=2 SV=1 - [MENT_HUMAN]	3	-0.6	-0.89	-0.60	-1.30	-0.94	-0.63	-0.33	-1.00	-0.63	-0.22	0.13	-0.63	-0.27	-0.48	-0.17	-0.84	-0.50	0.00
Q2TB1	Zinc finger protein 800 OS=Homo sapiens GN=ZNF800 PE=1 SV=1 -	1	-0.6	0.32	1.03	-0.48	-0.18	-0.42	0.31	-1.18	-0.87	-0.81	-0.04	-1.61	-1.31	-0.92	-0.18	-1.67	-1.38	0.01
Q7Z4V	Zinc finger protein 438 OS=Homo sapiens GN=ZNF438 PE=2 SV=1 -	1	-0.6	-0.03	-0.03	-0.09	-0.02	-0.32	-0.30	-0.34	-0.27	-1.50	-1.43	-1.55	-1.49	-0.54	-0.51	-0.54	-0.49	0.00
Q96P4	Collagen alpha-1(XXI) chain OS=Homo sapiens GN=COL21A1 PE=2	1	-0.6	0.38	0.20	-0.22	-0.79	0.09	-0.07	-0.47	-1.04	-0.07	-0.17	-0.66	-1.23	-0.90	-1.04	-1.44	-2.03	0.00
O1531	DNA-directed RNA polymerase III subunit RPC7 OS=Homo sapiens GN=POLR3G PE=1 SV=2	1	-0.6	-0.23	-0.63	-0.94	-0.36	-0.15	-0.53	-0.82	-0.24	-0.61	-0.94	-1.31	-0.74	-0.25	-0.62	-0.91	-0.35	0.00
Q4G0X	Coiled-coil domain-containing protein 40 OS=Homo sapiens GN=CCDC40 PE=2	1	-0.6	-0.14	-0.92	-0.25	0.39	-0.77	-1.41	-0.63	0.05	-1.72	-2.46	-1.69	-1.02	0.33	-0.46	0.12	0.96	0.02
Q96MR	Cilia- and flagella-associated protein 57 OS=Homo sapiens GN=CFAP57 PE=1 SV=3	1	-0.6	-1.07	-0.22	-0.91	-0.95	-0.95	-0.07	-0.74	-0.78	-0.62	0.31	-0.45	-0.49	-1.00	-0.12	-0.78	-0.84	0.00
P4874	Mannan-binding lectin serine protease 1 OS=Homo sapiens GN=MASP1 PE=1 SV=3 -	2	-0.6	-1.06	-0.85	0.19	0.61	-1.98	-1.75	-0.70	-0.27	-1.83	-1.55	-0.57	-0.16	-0.75	-0.51	0.55	0.95	0.02
O0026	Transcription initiation factor TFIID subunit 4 OS=Homo sapiens GN=TAF4 PE=1 SV=2 -	1	-0.6	-0.61	-0.16	-1.69	0.30	-0.36	0.11	-1.39	0.60	-0.75	-0.23	-1.82	0.17	-1.11	-0.63	-2.14	-0.16	0.01

Q8N2I	Serine/threonine-protein kinase 40 OS=Homo sapiens GN=STK40	1	-0.6	0.15	-0.25	-0.67	-0.87	0.24	-0.14	-0.54	-0.73	-0.51	-0.84	-1.32	-1.52	-0.20	-0.56	-0.96	-1.17	0.00
Q9Y5W	Sorting nexin-13 OS=Homo sapiens GN=SNX13 PE=1 SV=4 -	1	-0.6	-0.68	-0.05	-1.03	-0.93	-0.04	0.61	-0.35	-0.24	-0.20	0.51	-0.54	-0.45	-1.67	-1.00	-1.96	-1.88	0.01
P52848	Bifunctional heparan sulfate N-deacetylase/N-sulfotransferase 1 OS=Homo sapiens	1	-0.6	-0.97	-0.53	-0.16	0.44	-1.59	-1.14	-0.74	-0.06	-1.48	-1.06	-0.74	-0.07	-1.07	-0.73	-0.31	0.31	0.00
Q9H79	Uncharacterized protein C5orf42 OS=Homo sapiens GN=C5orf42	1	-0.6	-0.24	-0.25	-1.10	-1.41	-0.39	-0.38	-1.21	-1.52	-0.05	0.01	-0.91	-1.22	0.11	0.14	-0.69	-1.02	0.00
Q96C1	Probable ATP-dependent RNA helicase DHX58 OS=Homo sapiens GN=DHX58 PE=1 SV=1 -	1	-0.6	-0.60	-1.41	0.37	-0.63	-1.41	-2.19	-0.40	-1.39	-1.01	-1.74	-0.03	-1.03	0.26	-0.51	1.29	0.27	0.01
P21281	V-type proton ATPase subunit B, brain isoform OS=Homo sapiens GN=ATP6V1B2 PE=1	1	-0.6	-0.35	-0.54	-1.66	-0.09	-0.69	-0.87	-1.96	-0.39	-0.37	-0.50	-1.68	-0.11	0.04	-0.12	-1.22	0.34	0.00
P0171	Ig lambda chain V-II region BO OS=Homo sapiens PE=1 SV=1 -	1	-0.6	-0.69	-0.25	-0.91	-1.59	-0.46	0.15	-0.42	-0.76	-0.37	0.22	-0.59	-0.49	-0.79	-0.39	-1.30	-1.54	0.00
Q8WX	Ankyrin repeat and SOCS box protein 17 OS=Homo sapiens GN=ASB17 PE=2 SV=2 -	1	-0.6	-0.36	-1.19	0.00	-2.27	-0.12	-0.93	0.27	-1.99	-0.14	-1.00	0.14	-2.07	0.43	-0.37	0.83	-1.44	0.02
P4977	Translation initiation factor eIF-2B subunit beta OS=Homo sapiens GN=EIF2B2 PE=1 SV=3 -	1	-0.6	-1.89	-1.11	-1.27	0.54	-1.59	-0.79	-0.93	0.89	-0.97	-0.12	-0.35	1.46	-2.01	-1.20	-1.35	0.45	0.02
Q1275	Kinesin-like protein KIF1A OS=Homo sapiens GN=KIF1A PE=1	1	-0.6	-0.25	0.38	-0.38	-0.13	-0.86	-0.21	-0.95	-0.69	-1.47	-0.77	-1.59	-1.34	-0.69	-0.03	-0.77	-0.53	0.00
P12724	Eosinophil cationic protein OS=Homo sapiens GN=RNASE3 PE=1 SV=2	1	-0.6	0.05	0.18	-2.02	0.48	-0.15	0.00	-2.19	0.33	-0.53	-0.33	-2.60	-0.09	-0.51	-0.35	-2.53	-0.04	0.03
P54132	Bloom syndrome protein OS=Homo sapiens GN=BLM PE=1 SV=1 -	1	-0.6	0.16	-0.28	0.44	-0.98	-0.90	-1.32	-0.58	-2.00	-0.86	-1.23	-0.57	-2.00	0.24	-0.16	0.58	-0.86	0.00
P22792	Carboxypeptidase N subunit 2 OS=Homo sapiens GN=CPN2 PE=1	7	-0.6	-1.08	-1.54	-0.44	-0.34	-1.30	-1.73	-0.51	-0.31	-0.38	-1.00	-0.36	-0.49	-0.05	-0.68	-0.02	-0.14	0.00
8TBC	Zinc finger and SCAN domain-containing protein 18 OS=Homo sapiens GN=ZSCAN18 PE=2	1	-0.7	-1.71	-1.40	-1.36	-1.46	-0.62	-0.28	-0.22	-0.32	-0.44	-0.06	-0.08	-0.19	-0.83	-0.48	-0.42	-0.54	0.00
P01833	Polymeric immunoglobulin receptor OS=Homo sapiens GN=PIGR PE=1 SV=4 - [PIGR_HUMAN]	1	-0.7	-0.74	-0.57	-1.03	-1.72	-0.10	0.09	-0.36	-1.04	-0.14	-0.26	-0.68	-1.48	-0.36	-0.15	-0.60	-1.30	0.00
Q86UN	Reticulon-4 receptor-like 1 OS=Homo sapiens GN=RTN4RL1 PE=2	1	-0.7	-1.23	-0.43	-1.06	-1.96	-1.68	-0.87	-1.48	-2.37	0.09	0.95	0.26	-0.64	-0.10	0.73	0.12	-0.79	0.02
Q8IWY	Codanin-1 OS=Homo sapiens GN=CDAN1 PE=1 SV=4 -	2	-0.7	-0.99	-1.72	-0.29	0.68	-1.14	-1.85	-0.40	0.58	-1.04	-1.70	-0.34	0.63	-1.02	-1.42	-0.27	-0.40	0.01
Q1455	Phosphoribosyl pyrophosphate synthase-associated protein 1 OS=Homo sapiens	1	-0.7	-0.48	-1.08	-0.82	-0.13	-0.49	-1.07	-0.79	-0.09	-0.06	-0.58	-0.39	0.29	-1.17	-1.72	-1.45	-0.77	0.00
Q9UQE	Structural maintenance of chromosomes protein 3 OS=Homo sapiens GN=SMC3 PE=1 SV=2 -	1	-0.7	-0.28	0.40	-0.60	-0.49	-1.15	-0.74	-1.16	-1.45	-0.74	-0.16	-0.75	-0.98	-0.52	-0.28	-0.83	-1.08	0.00
Q0181	ATP-dependent 6-phosphofructokinase, platelet type OS=Homo sapiens GN=PFKP PE=1	1	-0.7	-1.11	-1.57	-2.02	-0.25	-0.54	-0.98	-1.40	0.37	-0.74	-1.13	-1.64	0.13	0.06	-0.36	-0.79	0.97	0.00
P60508	Syncytin-2 OS=Homo sapiens GN=ERVFRD-1 PE=1 SV=1 -	1	-0.7	-1.84	0.07	-1.23	-1.11	-0.77	1.16	-0.12	0.00	-1.13	0.86	-0.51	-0.39	-2.38	-0.43	-1.71	-1.61	0.01

Q9UJ4	Rab5 GDP/GTP exchange factor OS=Homo sapiens	1	-0.7	-0.25	-0.33	-2.66	-0.71	0.03	-0.03	-2.34	-0.38	0.13	0.12	-2.27	-0.32	0.14	0.09	-2.22	-0.28	0.01
P10523	S-arrestin OS=Homo sapiens GN=SAG PE=1 SV=3 - [ARRS_HUMAN]	1	-0.7	-0.64	-0.10	0.20	0.64	-1.87	-1.32	-1.00	-0.55	-1.36	-0.75	-0.52	-0.08	-1.68	-1.11	-0.79	-0.36	0.00
Q8WXI	Mucin-16 OS=Homo sapiens GN=MUC16 PE=1 SV=2 -	2	-0.7	-1.95	-0.49	1.15	0.00	-2.98	-1.50	0.16	-0.98	-2.89	-1.36	0.22	-0.94	-1.61	-0.11	1.55	0.39	0.05
Q9Y4K	Absent in melanoma 1 protein OS=Homo sapiens GN=AIM1 PE=1	1	-0.7	-1.23	-0.79	0.16	-0.10	-1.02	-0.56	0.41	0.15	-1.96	-1.45	-0.57	-0.84	-1.69	-1.21	-0.25	-0.52	0.00
P0DJI	Serum amyloid A-2 protein OS=Homo sapiens GN=SAA2 PE=1	2	-0.7	0.42	0.41	-0.21	-0.75	0.50	0.73	-0.10	-0.67	-0.88	-0.89	-1.59	-2.04	-1.20	-0.93	-1.97	-2.35	0.01
Q8NBJ	Golgi membrane protein 1 OS=Homo sapiens GN=GOLM1 PE=1 SV=1 ·	2	-0.7	-0.36	-1.24	-1.10	-0.59	-0.21	-1.07	-0.90	-0.39	-0.10	-0.92	-0.83	-0.33	-0.46	-1.31	-1.14	-0.65	0.00
Q9BZW	Testis-specific gene 10 protein OS=Homo sapiens GN=TSGA10	1	-0.7	-1.73	-2.04	-0.62	-0.25	-1.78	-2.07	-0.63	-0.26	-1.44	-1.68	-0.32	0.05	-0.33	-0.61	0.83	1.19	0.01
Q9Y2W	Tudor and KH domain-containing protein OS=Homo sapiens GN=TDRKH PE=1 SV=2	1	-0.7	0.69	-0.33	0.93	1.00	-1.13	-2.13	-0.85	-0.77	-1.22	-2.17	-0.97	-0.91	-0.88	-1.87	-0.59	-0.53	0.01
Q1302	A-kinase anchor protein 6 OS=Homo sapiens GN=AKAP6 PE=1 SV=3 -	1	-0.7	-0.81	-1.17	0.35	-0.47	-1.60	-1.94	-0.40	-1.22	-1.35	-1.64	-0.18	-1.01	-0.42	-0.74	0.80	-0.04	0.00
P5694	Breast cancer anti-estrogen resistance protein 1 OS=Homo sapiens GN=BCAR1	1	-0.7	-1.24	-0.91	-0.44	-0.71	-0.56	-0.21	0.28	0.01	-2.12	-1.71	-1.31	-1.58	-0.80	-0.43	0.06	-0.23	0.00
Q9BWU	Kanadaptin OS=Homo sapiens GN=SLC4A1AP PE=1 SV=1 -	1	-0.8	-0.61	-0.22	-0.29	-0.33	-0.82	-0.41	-0.46	-0.49	-0.67	-0.21	-0.34	-0.39	-2.00	-1.59	-1.63	-1.69	0.00
P0181	Ig heavy chain V-II region COR OS=Homo sapiens PE=1 SV=1 -	1	-0.8	-0.56	-0.31	-0.76	-3.07	0.40	0.79	0.34	-1.95	-0.67	-0.48	-0.90	-3.31	0.17	0.42	-0.01	-2.33	0.03
P6280	Histone H4 OS=Homo sapiens GN=HIST1H4A PE=1 SV=2 -	1	-0.8	-1.67	-0.27	-0.81	0.67	-2.22	-0.80	-1.32	0.16	-2.80	-1.33	-1.93	-0.46	-1.05	0.38	-0.13	1.33	0.01
Q96SW	Protein cereblon OS=Homo sapiens GN=CRBN PE=1 SV=1 -	1	-0.8	-0.13	0.50	-0.89	0.87	-1.17	-0.51	-1.89	-0.12	-1.53	-0.83	-2.28	-0.53	-1.20	-0.53	-1.90	-0.15	0.00
Q9NR4	Histone-lysine N-methyltransferase ASH1L OS=Homo sapiens GN=ASH1L	2	-0.8	-0.05	-3.45	-0.20	-0.51	0.39	-2.98	0.28	-0.02	0.05	-3.28	-0.10	-0.41	0.45	-2.91	0.35	0.03	0.05
Q1570	Disks large homolog 2 OS=Homo sapiens GN=DLG2 PE=1 SV=3 -	1	-0.8	0.08	-0.09	0.82	-2.38	-0.74	-0.90	0.03	-3.17	-0.36	-0.46	0.38	-2.83	-0.28	-0.42	0.51	-2.71	0.03
Q1636	Laminin subunit alpha-4 OS=Homo sapiens GN=LAMA4 PE=1 SV=4 ·	1	-0.8	-2.18	-1.92	-1.53	-1.55	-1.04	-0.76	-0.35	-0.37	-0.47	-0.13	0.19	0.17	-1.07	-0.77	-0.36	-0.40	0.00
Q9Y5X	Endothelial lipase OS=Homo sapiens GN=LIPG PE=1 SV=1 -	1	-0.8	-1.12	-0.49	-0.57	-0.05	-1.57	-0.92	-0.98	-0.46	-0.97	-0.26	-0.41	0.10	-1.81	-1.14	-1.20	-0.70	0.00
Q9C0A	SET domain-containing protein 5 OS=Homo sapiens GN=SETD5	1	-0.8	-0.30	-0.88	-0.45	-1.28	-0.05	-0.61	-0.16	-0.98	-0.32	-0.83	-0.46	-1.29	-0.87	-1.42	-0.96	-1.81	0.00
Q0281	Nucleobindin-1 OS=Homo sapiens GN=NUCB1 PE=1 SV=4 -	1	-0.8	-1.75	-1.26	0.22	-0.02	-2.74	-2.45	-0.95	-0.97	-1.97	-1.58	-0.35	-0.43	-0.59	-0.19	1.20	0.83	0.01
Q8N7W	BEN domain-containing protein 7 OS=Homo sapiens GN=BEND7	1	-0.8	-1.05	-1.09	-1.60	-0.16	-0.73	-0.74	-1.24	0.21	-0.70	-0.67	-1.25	0.19	-1.15	-1.16	-1.65	-0.22	0.00
Q8N47	Follistatin-related protein 5 OS=Homo sapiens GN=FSTL5 PE=2 SV=2 -	1	-0.8	1.31	0.02	-0.92	-0.04	-0.07	-1.33	-2.25	-1.37	0.66	-0.56	-1.56	-0.69	-0.39	-1.64	-2.56	-1.70	0.01
Q9295	Proteoglycan 4 OS=Homo sapiens GN=PRG4 PE=1 SV=2 -	16	-0.8	-1.21	-1.54	-0.21	0.09	-1.90	-2.36	-1.22	-0.62	-1.78	-1.85	-0.58	-0.42	-0.29	-0.79	0.60	0.97	0.00

Q9P2S	Ankyrin repeat and MYND domain-containing protein 1 OS=Homo sapiens GN=ANKMY1	1	-0.8	-1.60	-0.04	1.32	-0.33	-3.54	-1.96	-0.58	-2.23	-2.26	-0.62	0.67	-0.99	-1.72	-0.12	1.26	-0.41	0.03
P62502	Epididymal-specific lipocalin-6 OS=Homo sapiens GN=LCN6 PE=1	1	-0.8	-0.68	-1.82	-2.00	-1.00	0.18	-0.94	-1.11	-0.09	0.41	-0.66	-0.90	0.10	-0.51	-1.62	-1.78	-0.79	0.00
Q9BZD	Kinetochore protein Nuf2 OS=Homo sapiens GN=NUF2 PE=1 SV=2 -	1	-0.8	-1.05	-0.28	-1.07	-0.25	-0.99	-0.20	-0.97	-0.15	-1.79	-0.95	-1.80	-0.99	-1.10	-0.30	-1.07	-0.26	0.00
Q1501	Septin-2 OS=Homo sapiens GN=SEPT2 PE=1 SV=1 - [SEPT2_HUMAN]	1	-0.8	0.16	-0.46	-1.65	-1.99	0.31	-0.28	-1.45	-1.78	0.52	-0.02	-1.27	-1.62	0.15	-0.44	-1.60	-1.96	0.00
Q8ND2	RING finger protein 214 OS=Homo sapiens GN=RNF214 PE=1 SV=2	1	-0.8	-0.20	-0.64	-1.63	-0.67	-0.59	-1.01	-1.98	-1.02	-0.09	-0.46	-1.52	-0.57	-0.20	-0.61	-1.58	-0.64	0.00
O6047	Mannosyl-oligosaccharide 1,2-alpha-mannosidase IB OS=Homo sapiens GN=MAN1A2 PE=1	1	-0.8	-1.36	-1.56	-0.62	-0.18	-1.38	-1.56	-0.59	-0.15	-1.25	-1.38	-0.49	-0.06	-1.18	-1.34	-0.37	0.05	0.00
Q1357	Ras GTPase-activating-like protein IQGAP2 OS=Homo sapiens GN=IQGAP2 PE=1 SV=4	2	-0.8	-0.11	-2.77	-1.09	-1.35	0.65	-1.99	-0.29	-0.55	0.24	-2.34	-0.73	-1.00	0.66	-1.97	-0.27	-0.55	0.01
P4332	Beta-crystallin B2 OS=Homo sapiens GN=CRYBB2 PE=1	1	-0.8	-0.02	-2.07	0.04	-1.81	-0.69	-1.78	0.32	-1.50	-0.97	-2.01	0.01	-1.82	0.60	-1.42	0.68	-1.16	0.00
Q9273	USP6 N-terminal-like protein OS=Homo sapiens GN=USP6NL	1	-0.9	0.18	-2.57	-0.02	-0.84	-0.25	-2.97	-0.41	-1.22	-0.12	-2.80	-0.32	-1.14	0.67	-2.04	0.53	-0.30	0.01
Q1312	RE1-silencing transcription factor OS=Homo sapiens	2	-0.9	-0.03	-0.12	0.63	0.22	-1.49	-2.41	-0.79	-1.20	-1.62	-1.39	-0.95	-1.37	-1.06	-0.95	-0.35	-0.78	0.00
Q9P2G	Microtubule-associated protein 10 OS=Homo sapiens GN=MAP10	1	-0.9	-1.12	-0.40	-1.63	-1.14	-0.53	0.21	-0.99	-0.50	0.58	1.37	0.08	0.57	-2.65	-1.90	-3.10	-2.62	0.02
Q9Y2D	Exocyst complex component 6B OS=Homo sapiens	1	-0.9	-2.98	-0.70	-0.78	-0.49	-2.11	0.19	-0.07	0.54	-2.40	-0.48	-0.70	-0.25	-2.71	-0.22	-0.63	-0.10	0.01
Q8N9B	Ankyrin repeat domain-containing protein 42 OS=Homo sapiens GN=ANKRD42 PE=2	1	-0.9	0.40	1.58	2.12	0.43	-2.74	-1.55	-0.98	-2.30	-1.60	-0.36	0.12	-1.20	-2.86	-1.66	-1.09	-2.43	0.03
P0104	Cystatin-A OS=Homo sapiens GN=CSTA PE=1 SV=1 - [CYTA_HUMAN]	1	-0.9	-0.90	-1.07	-0.39	-0.75	-0.89	-1.04	-0.34	-0.69	-0.30	-0.39	0.22	-0.14	-2.02	-2.16	-1.46	-1.83	0.00
Q96LW	DNA-directed primase/polymerase protein OS=Homo sapiens GN=PRIMPOL	1	-0.9	0.21	-1.08	-1.75	-0.40	0.49	-0.79	-1.43	-0.08	0.21	-1.01	-1.74	-0.40	-0.82	-2.08	-2.72	-1.39	0.00
P2996	CD40 ligand OS=Homo sapiens GN=CD40LG PE=1 SV=1 -	1	-0.9	-0.82	-0.64	0.09	-0.35	-1.45	-1.25	-0.50	-0.94	-1.29	-1.04	-0.37	-0.83	-1.83	-1.61	-0.86	-1.32	0.00
Q1291	Receptor-type tyrosine-protein phosphatase eta OS=Homo sapiens GN=PTPRJ PE=1 SV=3 -	1	-0.9	-2.35	-2.54	0.28	0.14	-2.84	-3.00	-0.17	-0.30	-3.41	-3.14	-0.56	-1.13	-0.29	-0.44	2.40	2.24	0.05
P0988	DNA polymerase alpha catalytic subunit OS=Homo sapiens GN=POLA1 PE=1 SV=2 -	1	-0.9	-1.09	-2.46	-0.37	0.23	-1.92	-3.27	-1.16	-0.55	-1.35	-2.65	-0.63	-0.03	-0.18	-1.52	0.59	1.18	0.01
Q9NP7	Putative bifunctional UDP-N-acetylglucosamine transferase and deubiquitinase ALG13 OS=Homo sapiens	1	-1.0	-0.30	-1.17	-1.29	-0.23	-0.25	-1.10	-1.20	-0.14	-0.16	-0.95	-1.14	-0.09	-1.50	-2.33	-2.44	-1.39	0.00
Q3SYG	Protein PTHB1 OS=Homo sapiens GN=BBS9 PE=1 SV=1 -	2	-1.0	-0.44	-0.90	-1.34	0.33	-0.67	-1.21	-1.56	-0.08	-0.86	-1.48	-1.99	-0.32	-1.19	-1.65	-1.99	-0.41	0.00
Q9NQ3	Serine protease inhibitor Kazal-type 5 OS=Homo sapiens GN=SPINK5 PE=1 SV=2 -	1	-1.0	-0.34	-0.44	-2.18	-1.47	-0.31	-0.39	-2.11	-1.39	-0.09	-0.12	-1.92	-1.21	-0.22	-0.29	-2.00	-1.31	0.00

Q9H2F	3 beta-hydroxysteroid dehydrogenase type 7 OS=Homo sapiens GN=HSD3B7 PE=1 SV=2	1	-1.0	0.59	-1.62	-2.86	-1.50	0.46	-1.74	-2.96	-1.59	1.80	-0.34	-1.64	-0.28	0.79	-1.38	-2.60	-1.25	0.01
O6031	Germinal-center associated nuclear protein OS=Homo sapiens GN=MCM3AP	1	-1.0	-3.62	-0.81	-0.45	-0.33	-2.52	-0.98	-0.42	-0.44	-3.39	-0.87	-0.46	0.14	-3.02	0.03	0.44	0.54	0.01
O1507	Centrosomal protein of 290 kDa OS=Homo sapiens GN=CEP290	1	-1.0	-0.26	-0.37	-0.75	-0.26	-2.00	-2.09	-2.45	-1.96	-0.21	-0.26	-0.70	-0.22	-1.09	-1.17	-1.52	-1.05	0.00
P11532	Dystrophin OS=Homo sapiens GN=DMD PE=1 SV=3 - [DMD_HUMAN]	2	-1.0	0.00	-1.38	-2.97	-0.84	0.12	-1.25	-2.82	-0.69	0.96	-0.36	-2.01	0.11	-0.04	-1.39	-2.96	-0.85	0.00
P07197	Neurofilament medium polypeptide OS=Homo sapiens GN=NEFM PE=1 SV=3 - [NFM_HUMAN]	1	-1.1	-1.04	-1.69	-2.61	-0.22	0.29	-0.34	-1.24	1.16	-1.49	-2.07	-3.05	-0.66	-0.65	-1.27	-2.16	0.21	0.00
P09017	Homeobox protein Hox-C4 OS=Homo sapiens GN=HOXC4 PE=1 SV=2 -	1	-1.1	1.18	-2.34	-0.89	-0.18	0.31	-3.19	-1.73	-1.00	1.28	-2.17	-0.78	-0.07	-0.11	-3.60	-2.13	-1.43	0.01
Q2M38	Macrophage-expressed gene 1 protein OS=Homo sapiens GN=MPEG1 PE=2 SV=1 -	1	-1.1	-1.63	-4.31	-0.97	-0.93	-1.30	-3.96	-0.60	-0.56	-0.54	-3.15	0.14	0.16	0.47	-2.18	1.19	1.21	0.02
Q9970	Methionine synthase OS=Homo sapiens GN=MTR PE=1 SV=2 -	2	-1.1	-1.36	-0.74	-1.26	-0.91	-1.40	-0.76	-1.26	-1.37	-0.68	0.01	-0.57	-0.47	-2.03	-1.38	-1.87	-0.94	0.00
Q7Z40	Myosin-14 OS=Homo sapiens GN=MYH14 PE=1 SV=2 -	1	-1.1	0.30	-1.02	-2.49	-2.05	0.84	-0.47	-1.92	-1.47	0.71	-0.55	-2.08	-1.65	0.29	-1.00	-2.45	-2.02	0.00
Q5TB8	Centrosomal protein of 162 kDa OS=Homo sapiens GN=CEP162	3	-1.1	-0.21	-4.55	-3.10	1.06	0.16	-2.18	-3.19	0.97	0.35	-0.61	-2.96	1.19	0.19	-2.13	-3.40	0.74	0.04
Q8NEV	Myosin-IIIa OS=Homo sapiens GN=MYO3A PE=2 SV=2 -	1	-1.1	-0.58	-4.35	-1.10	0.60	0.46	-3.29	-0.02	1.68	0.23	-3.47	-0.28	1.41	-1.51	-5.25	-1.98	-0.30	0.05
A6NJ1	Putative V-set and immunoglobulin domain-containing-like protein IGHV4OR15-8 OS=Homo sapiens GN=IGHV4OR15-	2	-1.1	-1.18	-1.72	-2.73	-1.19	-0.74	-1.21	-2.16	-0.94	0.02	-0.49	-1.45	0.03	-0.48	-1.17	-2.06	-0.57	0.00
O7505	Immunoglobulin superfamily member 3 OS=Homo sapiens GN=IGSF3 PE=2 SV=3 -	1	-1.2	1.85	1.98	0.16	2.09	-1.94	-1.79	-3.59	-1.65	-1.26	-1.06	-2.95	-1.01	-2.01	-1.85	-3.65	-1.73	0.02
Q1323	cGMP-dependent protein kinase 2 OS=Homo sapiens GN=PRKG2	1	-1.2	-0.56	-0.96	-0.59	0.18	-2.39	-2.77	-2.38	-1.60	-2.06	-2.39	-2.08	-1.31	-0.01	-0.37	0.02	0.78	0.00
P20742	Pregnancy zone protein OS=Homo sapiens GN=PZP PE=1 SV=4 -	1	-1.2	0.06	-1.65	-1.50	-1.87	0.94	-0.75	-0.58	-0.94	0.52	-1.12	-1.03	-1.41	-1.11	-2.78	-2.61	-3.00	0.00
Q6IQ2	DENN domain-containing protein 5A OS=Homo sapiens GN=DENND5A PE=1 SV=2 -	2	-1.2	-0.97	-2.35	-0.61	-0.45	-2.41	-1.48	-0.11	-0.76	-1.18	-2.48	-0.80	-0.66	-1.05	-2.40	-0.63	-0.49	0.00
Q1457	Inositol 1,4,5-trisphosphate receptor type 3 OS=Homo sapiens GN=ITPR3 PE=1	1	-1.2	-0.59	-2.94	-0.39	-0.48	-0.85	-3.43	-1.31	-0.78	-0.56	-2.88	-0.83	-0.65	-0.47	-2.93	-0.43	-0.16	0.00
Q1418	Double C2-like domain-containing protein alpha OS=Homo sapiens GN=DOC2A PE=1 SV=5 -	1	-1.2	-0.34	-1.61	-0.44	-2.23	-0.28	-1.53	-0.34	-2.12	-0.37	-1.57	-0.46	-2.26	-0.78	-2.01	-0.82	-2.63	0.00
Q9UPU	TBC1 domain family member 2B OS=Homo sapiens GN=TBC1D2B	1	-1.2	-3.63	-0.07	-0.16	-0.49	-4.53	-0.95	-1.02	-1.34	-3.45	0.18	0.03	-0.30	-3.64	-0.04	-0.11	-0.45	0.01
Q8IZT	Protein FAM9C OS=Homo sapiens GN=FAM9C PE=2 SV=1 -	1	-1.3	-0.74	-2.08	-1.30	-0.86	-1.15	-2.48	-1.67	-1.23	-0.85	-2.12	-1.40	-0.97	-0.36	-1.67	-0.87	-0.44	0.00

P5707	Ubiquitin-associated and SH3 domain-containing protein A OS=Homo sapiens GN=UBASH3A	1	-1.3	-4.33	-0.66	-0.20	-1.46	-3.97	-0.28	0.20	-1.06	-2.93	0.81	1.21	-0.06	-4.59	-0.89	-0.40	-1.68	0.01
Q9297	Transportin-1 OS=Homo sapiens GN=TNPO1 PE=1 SV=2 -	1	-1.3	-3.14	-0.80	-1.50	-0.72	-2.60	-0.25	-0.93	-0.14	-2.79	-0.38	-1.14	-0.36	-3.20	-0.83	-1.51	-0.74	0.00
Q96AN	Transmembrane protein 143 OS=Homo sapiens GN=TMEM143 PE=2	1	-1.3	-5.74	-1.47	-0.69	-0.22	-5.09	-0.80	-0.01	0.47	-4.60	-0.26	0.45	0.92	-4.77	-0.46	0.33	0.79	0.04
Q5JW9	Protein FAM26D OS=Homo sapiens GN=FAM26D PE=1	1	-1.3	0.09	0.87	-4.47	-0.27	-0.41	0.40	-4.92	-0.72	-0.47	0.39	-5.02	-0.83	-0.52	0.30	-5.02	-0.84	0.03
Q2M38	WASH complex subunit 7 OS=Homo sapiens GN=KIAA1033 PE=1	1	-1.3	0.02	-3.62	-1.53	-0.76	0.05	-3.58	-1.47	-0.70	0.54	-3.03	-1.00	-0.24	-0.08	-3.69	-1.58	-0.82	0.00
O1477	Tripeptidyl-peptidase 1 OS=Homo sapiens GN=TPP1 PE=1 SV=2 -	1	-1.4	-2.50	-1.09	-2.04	-1.98	-1.55	-0.05	-1.03	-0.87	-1.82	-0.43	-1.36	-1.30	-2.11	-0.50	-1.49	-1.55	0.00
Q8NBU	ATPase family AAA domain-containing protein 1 OS=Homo sapiens GN=ATAD1 PE=1 SV=1 -	1	-1.4	-0.87	-2.32	-1.08	-0.52	-1.38	-2.81	-1.55	-0.99	-0.52	-1.91	-0.73	-0.18	-1.52	-2.95	-1.68	-1.14	0.00
Q8IYJ1	Copine-9 OS=Homo sapiens GN=CPNE9 PE=1 SV=3 -	1	-1.4	-0.36	-1.94	-1.59	0.38	-1.07	-2.63	-2.26	-0.29	-1.66	-3.17	-2.87	-0.92	-0.45	-2.00	-1.62	0.32	0.00
Q9275	Transcription factor AP-2 gamma OS=Homo sapiens GN=TFAP2C	1	-1.4	-1.21	-2.31	-1.31	-1.57	-1.25	-2.33	-1.31	-1.57	-0.47	-1.50	-0.56	-0.83	-1.24	-2.31	-1.29	-1.57	0.00
P0181	Ig heavy chain V-II region OU OS=Homo sapiens PE=1 SV=1 -	1	-1.5	-2.42	2.57	-0.61	-1.62	-4.74	0.26	-2.89	-3.90	-1.33	3.72	0.48	-0.54	-5.08	-0.06	-3.22	-4.25	0.03
O4344	Peptidyl-prolyl cis-trans isomerase H OS=Homo sapiens GN=PPIH PE=1	1	-1.5	-0.11	-2.52	-1.97	-0.96	-0.12	-2.51	-1.94	-0.93	-0.29	-2.63	-2.14	-1.14	-0.39	-2.77	-2.19	-1.20	0.00
P3840	Guanine nucleotide-binding protein G(olf) subunit alpha OS=Homo sapiens GN=GNAL PE=1	1	-1.5	0.34	-4.04	-3.14	0.03	-0.40	-4.76	-3.84	-0.66	0.61	-3.70	-2.86	0.31	1.43	-2.92	-2.00	1.16	0.01
P5576	NHP2-like protein 1 OS=Homo sapiens GN=NHP2L1 PE=1 SV=3	1	-1.5	-0.97	-5.52	-2.33	-1.11	0.37	-4.16	-0.96	0.27	-0.23	-4.71	-1.58	-0.37	0.64	-3.87	-0.67	0.54	0.01
Q9UJA	tRNA (adenine(58)-N(1))-methyltransferase non-catalytic subunit TRM6 OS=Homo sapiens GN=TRMT6 PE=1 SV=1	1	-1.6	-1.47	-3.17	-1.08	-0.55	-1.44	-3.11	-1.01	-0.47	-1.41	-3.04	-1.02	-0.49	-1.79	-3.45	-1.35	-0.83	0.00
Q86VI	Ras GTPase-activating-like protein IQGAP3 OS=Homo sapiens GN=IQGAP3 PE=1 SV=2	1	-1.6	1.98	-2.08	-0.50	1.39	-0.98	-5.01	-3.41	-1.52	0.14	-3.84	-2.32	-0.44	-0.58	-4.60	-3.00	-1.13	0.01
O6093	Nibrin OS=Homo sapiens GN=NBN PE=1 SV=1 -	1	-1.6	-1.61	-0.67	-0.83	-5.98	-0.93	0.03	-0.11	-5.26	-1.14	-0.13	-0.35	-5.51	-0.30	0.67	0.53	-4.63	0.01
Q5JR5	Microtubule-associated tumor suppressor candidate 2 OS=Homo sapiens GN=MTUS2	2	-1.7	-0.90	-4.33	-2.38	-1.21	-0.58	-3.99	-2.03	-1.06	0.39	-2.97	-1.09	0.09	-0.34	-3.74	-1.77	-0.82	0.00
Q9H84	Acyl-CoA dehydrogenase family member 9, mitochondrial OS=Homo sapiens GN=ACAD9	1	-1.7	-1.72	-0.86	-1.60	-1.85	-2.64	-1.76	-2.48	-2.73	-1.27	-0.34	-1.14	-1.40	-2.01	-1.12	-1.83	-2.10	0.00
Q9NZV	Potassium voltage-gated channel subfamily D member 2 OS=Homo sapiens GN=KCND2	1	-1.8	-0.91	-2.23	-2.95	-0.92	-0.87	-2.17	-2.87	-0.84	-0.96	-2.21	-2.99	-0.97	-1.11	-2.40	-3.09	-1.08	0.00
Q9H3D	Tumor protein 63 OS=Homo sapiens GN=TP63 PE=1 SV=1 -	1	-1.8	-5.20	-1.63	-0.23	-0.67	-5.45	-1.86	-0.44	-0.87	-4.53	-0.89	0.44	0.01	-5.27	-1.67	-0.24	-0.69	0.00
Q1373	Sodium/potassium-transporting ATPase subunit alpha-4 OS=Homo sapiens	1	-2.1	-4.49	-2.95	-2.81	-2.68	-2.94	-1.38	-1.23	-1.08	-2.71	-1.10	-1.02	-0.89	-3.25	-1.68	-1.52	-1.40	0.00

Q8NI7	Kinesin-like protein KIF18A OS=Homo sapiens GN=KIF18A	1	-2.1	0.60	-2.34	-3.31	-1.57	-0.21	-3.02	-4.38	-2.00	0.47	-2.40	-3.43	-1.70	-0.53	-3.33	-4.68	-2.33	0.00

0.82741193
