# Supplementary material for: Sex-specific correlation of IGFBP-2 and IGFBP-3 with vitamin D status in adults with obesity: a cross-sectional serum proteomics study
Source: Nutr Diabetes. 2018 Oct 4;8:54. doi: 10.1038/s41387-018-0063-8 (PMC6172285; doi:10.1038/s41387-018-0063-8)
Supplement: Supplementary file 2 — Supplementary Table 2. Differentially expressed proteins in high vs. low vitamin D status (females) [file 41387_2018_63_MOESM2_ESM.pdf]

xpressed proteins in high vs. low vitD status (females)

Accession	Description	Σ# Unique Peptides	Mean iTRAQ log2ratio high vs. lkow vitD status	117/ 113	117/ 114	117/ 115	117/ 116	118/ 113	118/ 114	118/ 115	118/ 116	119/ 113	119/ 114	119/ 115	119/ 116	121/ 113	121/ 114	121/ 115	121/ 116	ttest
Q9NX95	Syntabulin OS=Homo sapiens GN=SYBU PE=1 SV=2 - [SYBU_HUMAN]	1	2.8	2.17	1.83	2.30	1.01	3.99	3.67	4.08	2.83	3.20	2.89	3.28	2.09	3.22	2.89	3.40	2.05	0.00
O95886	Disks large-associated protein 3 OS=Homo sapiens GN=DLGAP3 PE=1 SV=3 - [DLGP3_HUMAN]	1	2.6	4.48	4.80	4.53	1.98	3.99	4.32	4.00	1.49	3.29	3.64	3.28	0.84	0.86	1.18	0.95	####	0.00
Q5VTR2	E3 ubiquitin-protein ligase BRE1A OS=Homo sapiens GN=RNF20 PE=1 SV=2 - [BRE1A_HUMAN]	1	2.3	2.72	3.06	3.43	3.08	1.85	2.21	2.51	2.22	0.85	1.21	1.50	1.26	2.45	2.80	3.20	2.81	0.00
P19367	Hexokinase-1 OS=Homo sapiens GN=HK1 PE=1 SV=3 - [HXK1_HUMAN]	1	2.3	####	1.36	####	####	3.41	4.92	3.43	3.53	2.20	3.72	2.20	2.37	2.08	3.58	2.18	2.19	0.00
P62328	Thymosin beta-4 OS=Homo sapiens GN=TMSB4X PE=1 SV=2 - [TYB4_HUMAN]	2	2.3	4.27	4.53	4.92	2.54	0.97	1.37	1.42	####	3.03	3.27	3.78	1.05	1.88	2.34	2.50	0.00	0.00
Q5J8X5	Membrane-spanning 4-domains subfamily A member 13 OS=Homo sapiens GN=MS4A13 PE=2 SV=2 - [M4A13_HUMAN]	1	2.3	4.00	2.26	1.06	0.04	3.76	2.03	0.78	####	4.93	3.21	1.93	1.02	5.12	3.39	2.23	1.16	0.00
Q9NZJ5	Eukaryotic translation initiation factor 2-alpha kinase 3 OS=Homo sapiens GN=EIF2AK3 PE=1 SV=3 - [E2AK3_HUMAN]	1	2.2	####	####	####	####	2.68	2.93	2.43	2.72	3.52	3.78	3.25	3.61	2.89	3.14	2.73	2.92	0.00
Q9UHX1	Poly(U)-binding-splicing factor PUF60 OS=Homo sapiens GN=PUF60 PE=1 SV=1 - [PUF60_HUMAN]	1	1.9	2.23	2.68	1.17	0.00	2.15	2.62	1.05	####	2.61	3.09	1.50	0.43	3.51	3.97	2.49	1.27	0.00
P04844	Dolichyl-diphosphooligosaccharide--protein glycosyltransferase subunit 2 OS=Homo sapiens GN=RPN2 PE=1 SV=3 - [RPN2_HUMAN]	1	1.8	####	####	####	0.30	3.34	3.02	3.10	3.78	1.13	0.82	0.87	1.61	3.11	2.78	2.96	3.54	0.00
O75828	Carbonyl reductase [NADPH] 3 OS=Homo sapiens GN=CBR3 PE=1 SV=3 - [CBR3_HUMAN]	1	1.8	4.35	4.79	4.63	2.66	0.53	1.00	0.77	####	2.46	2.93	2.68	0.82	0.63	1.09	0.95	####	0.00
Q96EK7	Constitutive coactivator of peroxisome proliferator-activated receptor gamma OS=Homo sapiens GN=FAM120B PE=1 SV=1 - [F120B_HUMAN]	1	1.7	4.36	3.03	3.37	0.41	3.72	2.41	2.68	####	2.91	1.61	1.86	####	2.15	0.83	1.20	####	0.00
P48058	Glutamate receptor 4 OS=Homo sapiens GN=GRIA4 PE=2 SV=2 - [GRIA4_HUMAN]	1	1.7	2.49	2.99	1.10	0.54	1.82	2.34	0.39	####	2.87	3.40	1.42	0.96	2.44	2.95	1.10	0.48	0.00
P26440	Isovaleryl-CoA dehydrogenase, mitochondrial OS=Homo sapiens GN=IVD PE=1 SV=1 - [IVD_HUMAN]	1	1.6	1.04	1.51	0.75	####	1.19	1.69	0.87	####	1.86	2.37	1.52	0.66	3.30	3.79	3.06	2.04	0.00
Q13492	Phosphatidylinositol-binding clathrin assembly protein OS=Homo sapiens GN=PICALM PE=1 SV=2 - [PICAL_HUMAN]	1	1.5	2.19	2.86	2.66	0.30	2.09	2.77	2.52	0.20	2.11	2.81	2.52	0.27	0.48	1.16	0.99	####	0.00
Q8TE73	Dynein heavy chain 5, axonemal OS=Homo sapiens GN=DNAH5 PE=1 SV=3 - [DYH5_HUMAN]	1	1.5	0.14	0.88	####	####	1.00	1.75	0.80	0.18	3.12	3.88	2.90	2.35	1.82	2.56	1.70	0.99	0.00
Q9BVW5	TIMELESS-interacting protein OS=Homo sapiens GN=TIPIN PE=1 SV=2 - [TIPIN_HUMAN]	1	1.4	1.89	2.08	1.10	1.35	2.17	2.37	1.33	1.62	1.57	1.79	0.73	1.08	1.29	1.49	0.55	0.74	0.00
Q9Y6N5	Sulfide:quinone oxidoreductase, mitochondrial OS=Homo sapiens GN=SQRDL PE=1 SV=1 - [SQRD_HUMAN]	1	1.4	1.82	1.39	2.11	0.77	2.12	1.72	2.38	1.08	1.06	0.66	1.30	0.06	1.76	1.35	2.11	0.71	0.00

A6NCD4	Uncharacterized protein C10orf131 OS=Homo sapiens GN=C10orf131 PE=2 SV=3 - [CJ131_HUMAN]	1	1.4	3.42	2.76	0.96	0.07	2.64	2.18	0.26	####	2.27	1.64	####	####	3.54	3.01	1.03	0.05	0.00
P53370	Nucleoside diphosphate-linked moiety X motif 6 OS=Homo sapiens GN=NUDT6 PE=1 SV=2 - [NUDT6_HUMAN]	1	1.4	####	0.50	1.01	0.16	####	0.69	1.15	0.34	2.20	4.28	4.71	3.96	####	1.90	2.45	1.55	0.01
P60985	Keratinocyte differentiation-associated protein OS=Homo sapiens GN=KRTDAP PE=1 SV=1 - [KTDAP_HUMAN]	2	1.3	2.47	2.48	1.41	0.61	2.44	2.56	1.19	0.66	2.08	2.17	0.81	0.32	0.87	0.97	0.13	####	0.00
Q9H254	Spectrin beta chain, non-erythrocytic 4 OS=Homo sapiens GN=SPTBN4 PE=1 SV=2 - [SPTN4_HUMAN]	2	1.3	1.88	1.04	1.38	1.52	1.39	0.92	0.54	0.72	1.80	1.74	1.15	0.97	1.78	1.41	1.07	1.16	0.00
Q92833	Protein Jumonji OS=Homo sapiens GN=JARID2 PE=1 SV=2 - [JARD2_HUMAN]	2	1.2	0.83	0.33	1.64	0.53	1.11	0.63	1.88	0.68	0.94	0.47	1.70	0.70	2.12	1.63	2.97	1.82	0.00
Q8IWG1	WD repeat-containing protein 63 OS=Homo sapiens GN=WDR63 PE=2 SV=1 - [WDR63_HUMAN]	1	1.2	1.66	1.63	0.82	0.57	0.95	1.10	0.41	0.16	0.96	1.57	0.24	0.26	3.71	3.66	0.52	1.01	0.00
Q15942	Zyxin OS=Homo sapiens GN=ZYX PE=1 SV=1 - [ZYX_HUMAN]	5	1.2	2.44	2.54	3.38	2.87	####	####	0.32	0.28	1.68	1.81	2.55	2.35	####	####	0.07	0.45	0.00
Q6KB66	Keratin, type II cytoskeletal 80 OS=Homo sapiens GN=KRT80 PE=1 SV=2 - [K2C80_HUMAN]	1	1.2	####	####	####	####	2.67	2.80	2.92	2.64	2.05	2.19	2.29	2.06	0.16	0.28	0.50	0.11	0.00
Q5VUY2	Arylacetamide deacetylase-like 4 OS=Homo sapiens GN=AADACL4 PE=2 SV=1 - [ADCL4_HUMAN]	1	1.2	1.59	1.72	1.50	1.08	0.98	1.67	1.39	0.66	0.75	1.29	0.98	0.42	1.11	1.53	1.34	0.63	0.00
Q08378	Golgin subfamily A member 3 OS=Homo sapiens GN=GOLGA3 PE=1 SV=2 - [GOGA3_HUMAN]	2	1.2	1.75	1.76	1.99	1.20	1.08	1.12	1.28	0.53	1.13	1.17	1.32	0.63	0.94	0.96	1.22	0.38	0.00
P05019	Insulin-like growth factor I OS=Homo sapiens GN=IGF1 PE=1 SV=1 - [IGF1_HUMAN]	3	1.1	0.68	0.52	2.38	0.43	1.00	1.27	2.78	0.76	1.28	1.70	1.97	1.00	0.46	0.35	1.62	0.17	0.00
Q9ULV0	Unconventional myosin-Vb OS=Homo sapiens GN=MYO5B PE=1 SV=3 - [MYO5B_HUMAN]	2	1.1	0.28	1.10	0.34	####	0.98	1.70	1.03	####	2.00	3.12	2.03	1.41	1.01	2.31	1.14	0.55	0.00
Q96NZ9	Proline-rich acidic protein 1 OS=Homo sapiens GN=PRAP1 PE=1 SV=2 - [PRAP1_HUMAN]	2	1.1	2.58	1.28	2.27	0.87	1.17	####	0.94	####	1.78	0.55	1.51	0.24	2.15	0.82	1.81	0.54	0.00
Q6UWL6	Kin of IRRE-like protein 2 OS=Homo sapiens GN=KIRREL2 PE=1 SV=2 - [KIRR2_HUMAN]	1	1.1	####	####	0.10	0.70	1.16	1.12	1.58	2.22	####	####	0.15	0.85	2.48	2.44	2.99	3.53	0.00
Q76KX8	Zinc finger protein 534 OS=Homo sapiens GN=ZNF534 PE=2 SV=1 - [ZN534_HUMAN]	1	1.1	0.68	1.53	1.17	0.95	0.76	1.08	1.14	0.70	1.56	1.89	1.93	1.55	0.28	1.14	0.82	0.62	0.00
Q5S007	Leucine-rich repeat serine/threonine-protein kinase 2 OS=Homo sapiens GN=LRRK2 PE=1 SV=2 - [LRRK2_HUMAN]	1	1.1	####	1.53	0.85	0.03	####	1.49	0.75	####	1.42	3.30	2.54	1.82	0.31	2.18	1.53	0.66	0.00
O00194	Ras-related protein Rab-27B OS=Homo sapiens GN=RAB27B PE=1 SV=4 - [RB27B_HUMAN]	2	1.1	3.36	2.37	4.49	3.66	####	####	0.37	####	1.07	0.11	2.15	1.42	0.24	####	1.42	0.54	0.02
P06317	Ig lambda chain V-VI region SUT OS=Homo sapiens PE=1 SV=1 - [LV603_HUMAN]	1	1.1	1.55	####	0.65	####	1.87	####	0.93	####	4.41	1.80	3.46	2.28	2.20	####	1.35	0.02	0.02
O94964	Protein SOGA1 OS=Homo sapiens GN=SOGA1 PE=1 SV=2 - [SOGA1_HUMAN]	1	1.1	0.38	0.94	1.31	####	2.17	2.75	3.06	1.77	0.79	1.38	1.66	0.44	####	0.46	0.86	####	0.00
Q9UM19	Hippocalcin-like protein 4 OS=Homo sapiens GN=HPCAL4 PE=2 SV=3 - [HPCL4_HUMAN]	1	1.1	2.87	1.95	0.88	0.25	2.15	1.25	0.12	####	1.88	0.99	####	####	2.93	2.02	0.99	0.30	0.00
Q92791	Synaptonemal complex protein SC65 OS=Homo sapiens GN=LEPREL4 PE=1 SV=1 - [SC65_HUMAN]	1	1.1	2.31	1.59	0.23	0.71	1.73	1.03	####	0.13	2.58	1.89	0.44	1.03	2.03	1.32	0.00	0.42	0.00
O75335	Liprin-alpha-4 OS=Homo sapiens GN=PPFIA4 PE=2 SV=3 - [LIPA4_HUMAN]	1	1.0	####	####	####	####	2.44	2.64	2.27	2.63	1.46	1.68	1.28	1.70	0.54	0.74	0.46	0.72	0.00

Q9NYK1	Toll-like receptor 7 OS=Homo sapiens GN=TLR7 PE=1 SV=1 - [TLR7_HUMAN]	1	1.0	1.71	1.05	1.25	0.73	1.44	0.76	0.72	0.43	0.68	0.23	0.16	####	2.40	1.82	1.87	1.38	0.00
Q7LFX5	Carbohydrate sulfotransferase 15 OS=Homo sapiens GN=CHST15 PE=1 SV=1 - [CHSTF_HUMAN]	1	1.0	3.59	4.42	4.35	4.21	####	####	####	####	####	0.21	0.62	####	####	0.70	0.68	0.43	0.05
Q9Y6Q9	Nuclear receptor coactivator 3 OS=Homo sapiens GN=NCOA3 PE=1 SV=1 - [NCOA3_HUMAN]	1	1.0	1.27	0.59	####	####	1.73	1.32	0.64	0.43	2.76	2.48	1.48	1.38	1.26	0.85	0.25	####	0.00
Q96HP0	Dedicator of cytokinesis protein 6 OS=Homo sapiens GN=DOCK6 PE=1 SV=3 - [DOCK6_HUMAN]	2	1.0	2.73	2.09	0.46	1.29	2.96	2.34	0.65	1.52	1.45	0.84	####	0.06	1.17	0.54	####	####	0.00
P02656	Apolipoprotein C-III OS=Homo sapiens GN=APOC3 PE=1 SV=1 - [APOC3_HUMAN]	9	1.0	1.54	1.77	1.64	0.60	1.22	1.44	1.25	0.35	0.57	0.76	0.52	####	1.17	1.41	1.32	0.37	0.00
P11684	Uteroglobin OS=Homo sapiens GN=SCGB1A1 PE=1 SV=1 - [UTER_HUMAN]	3	1.0	0.98	1.39	1.47	1.80	0.32	0.93	0.83	1.20	####	0.40	0.36	0.66	1.01	1.34	1.44	1.78	0.00
Q9BRA0	N-alpha-acetyltransferase 38, NatC auxiliary subunit OS=Homo sapiens GN=NAA38 PE=1 SV=1 - [LSMD1_HUMAN]	1	1.0	0.35	0.85	####	0.33	0.61	1.13	0.15	0.59	1.61	2.15	1.13	1.64	1.24	1.75	0.86	1.21	0.00
Q86UX7	Fermitin family homolog 3 OS=Homo sapiens GN=FERMT3 PE=1 SV=1 - [URP2_HUMAN]	1	0.9	2.47	1.68	3.25	2.59	1.25	####	1.15	0.53	1.22	####	1.39	0.84	0.52	####	0.38	####	0.01
Q9HAY6	Beta,beta-carotene 15,15'-monooxygenase OS=Homo sapiens GN=BCO1 PE=1 SV=1 - [BCDO1_HUMAN]	1	0.9	1.45	1.48	0.68	####	0.98	1.03	0.17	####	1.52	1.58	0.70	####	2.34	2.39	1.62	0.54	0.00
Q9H4M9	EH domain-containing protein 1 OS=Homo sapiens GN=EHD1 PE=1 SV=2 - [EHD1_HUMAN]	1	0.9	2.56	1.56	3.11	2.51	0.20	####	0.71	0.15	1.12	0.15	1.61	1.12	0.36	####	0.95	0.30	0.00
Q96QR1	Secretoglobin family 3A member 1 OS=Homo sapiens GN=SCGB3A1 PE=1 SV=2 - [SG3A1_HUMAN]	1	0.9	0.73	0.29	1.82	####	0.50	0.08	1.55	####	2.04	1.63	3.07	1.16	0.73	0.30	1.85	####	0.00
P46439	Glutathione S-transferase Mu 5 OS=Homo sapiens GN=GSTM5 PE=1 SV=3 - [GSTM5_HUMAN]	1	0.9	####	####	####	####	3.80	3.40	3.43	3.91	0.84	0.46	0.46	1.00	0.21	####	####	0.32	0.05
A8MTI9	Putative serine protease 47 OS=Homo sapiens GN=PRSS47 PE=5 SV=2 - [PRS47_HUMAN]	1	0.9	0.50	0.83	0.24	####	1.54	1.89	1.24	0.72	0.67	1.03	0.35	####	1.72	2.06	1.50	0.89	0.00
Q5CZ79	Ankyrin repeat domain-containing protein 20B OS=Homo sapiens GN=ANKRD20A8P PE=2 SV=2 - [AN20B_HUMAN]	1	0.9	3.19	2.38	1.17	####	2.86	2.08	0.81	####	1.32	0.54	####	####	3.07	2.28	1.10	####	0.05
Q9Y2I9	TBC1 domain family member 30 OS=Homo sapiens GN=TBC1D30 PE=1 SV=2 - [TBC30_HUMAN]	2	0.9	####	0.50	0.55	####	1.26	2.52	2.64	1.91	0.63	1.90	1.99	1.32	####	0.66	0.93	####	0.01
Q4VX67	Spermatogenesis-associated protein 31A4 OS=Homo sapiens GN=SPATA31A4 PE=3 SV=2 - [S31A4_HUMAN]	1	0.9	1.04	1.23	0.85	0.53	1.55	1.75	1.31	1.04	0.62	0.84	0.37	0.16	0.91	1.11	0.76	0.39	0.00
O75015	Low affinity immunoglobulin gamma Fc region receptor III-B OS=Homo sapiens GN=FCGR3B PE=1 SV=2 - [FCG3B_HUMAN]	4	0.9	1.29	1.41	1.57	1.16	1.30	1.36	1.23	1.33	0.14	0.15	0.31	0.30	0.58	0.79	0.97	0.56	0.00
P61769	Beta-2-microglobulin OS=Homo sapiens GN=B2M PE=1 SV=1 - [B2MG_HUMAN]	6	0.9	1.21	0.53	1.09	0.89	0.64	####	0.74	0.33	2.10	1.56	2.60	2.21	0.64	####	0.45	####	0.00
P11532	Dystrophin OS=Homo sapiens GN=DMD PE=1 SV=3 - [DMD_HUMAN]	1	0.9	1.02	1.31	1.24	0.84	0.59	0.88	0.76	0.40	0.86	1.17	1.02	0.72	0.79	1.08	1.05	0.59	0.00
Q96QE3	ATPase family AAA domain-containing protein 5 OS=Homo sapiens GN=ATAD5 PE=1 SV=4 - [ATAD5_HUMAN]	1	0.9	####	1.36	2.25	####	0.11	1.53	2.37	####	0.62	2.06	2.86	0.54	####	0.72	1.65	####	0.01
P14735	Insulin-degrading enzyme OS=Homo sapiens GN=IDE PE=1 SV=4 - [IDE_HUMAN]	1	0.9	0.03	1.81	1.71	0.12	####	1.07	0.53	####	1.19	2.99	3.20	1.33	####	1.37	1.04	####	0.01
P10124	Serglycin OS=Homo sapiens GN=SRGN PE=1 SV=3 - [SRGN_HUMAN]	2	0.9	1.21	0.86	1.75	1.52	0.88	0.52	1.23	1.27	0.45	0.21	0.90	1.20	0.21	0.00	1.06	0.95	0.00

P78352	Disks large homolog 4 OS=Homo sapiens GN=DLG4 PE=1 SV=3 - [DLG4_HUMAN]	1	0.9	2.42	1.21	0.97	####	2.33	1.37	0.85	####	2.01	0.58	####	####	2.59	1.66	1.20	####	0.01
Q01469	Fatty acid-binding protein, epidermal OS=Homo sapiens GN=FABP5 PE=1 SV=3 - [FABP5_HUMAN]	1	0.9	0.79	0.82	1.70	0.75	1.04	1.08	1.91	1.00	0.68	0.73	1.53	0.69	0.13	0.17	1.08	0.08	0.00
P01160	Natriuretic peptides A OS=Homo sapiens GN=NPPA PE=1 SV=1 - [ANF_HUMAN]	1	0.9	1.05	1.58	1.23	0.16	0.98	1.53	1.12	0.10	1.52	2.08	1.65	0.68	0.11	0.66	0.34	####	0.00
Q96EE4	Coiled-coil domain-containing protein 126 OS=Homo sapiens GN=CCDC126 PE=2 SV=2 - [CC126_HUMAN]	2	0.9	1.76	1.09	1.36	0.99	1.04	0.60	1.00	0.40	0.93	0.47	0.63	0.20	1.17	0.66	1.04	0.49	0.00
Q969H8	UPF0556 protein C19orf10 OS=Homo sapiens GN=C19orf10 PE=1 SV=1 - [CS010_HUMAN]	1	0.9	1.84	1.31	1.50	1.80	0.26	####	####	0.22	1.70	1.20	1.30	1.71	0.54	0.02	0.24	0.49	0.00
O43164	E3 ubiquitin-protein ligase Praja-2 OS=Homo sapiens GN=PJA2 PE=1 SV=4 - [PJA2_HUMAN]	1	0.9	1.08	1.32	1.32	####	1.19	1.46	1.40	####	0.00	0.28	0.19	####	1.93	2.19	2.22	0.65	0.00
Q8IXF0	Neuronal PAS domain-containing protein 3 OS=Homo sapiens GN=NPAS3 PE=2 SV=1 - [NPAS3_HUMAN]	1	0.9	1.82	1.54	1.63	0.70	0.36	0.11	0.14	####	1.13	0.89	0.89	0.06	1.69	1.43	1.55	0.56	0.00
P01824	Ig heavy chain V-II region WAH OS=Homo sapiens PE=1 SV=1 - [HV206_HUMAN]	1	0.9	0.23	0.40	0.01	####	0.94	1.12	0.67	####	2.03	2.22	1.75	1.08	1.29	1.46	1.10	0.27	0.00
P02776	Platelet factor 4 OS=Homo sapiens GN=PF4 PE=1 SV=2 - [PLF4_HUMAN]	4	0.8	0.40	0.27	0.23	0.94	0.24	####	####	0.69	2.31	2.37	1.29	2.70	0.66	0.57	0.10	1.19	0.00
P22466	Galanin peptides OS=Homo sapiens GN=GAL PE=1 SV=3 - [GALA_HUMAN]	1	0.8	1.52	2.26	2.14	####	1.57	2.33	2.15	0.03	0.68	1.44	1.23	####	####	0.53	0.44	####	0.01
Q8TAG9	Exocyst complex component 6 OS=Homo sapiens GN=EXOC6 PE=1 SV=3 - [EXOC6_HUMAN]	2	0.8	1.30	1.02	0.64	1.14	1.15	0.89	0.46	0.99	0.88	0.62	0.16	0.76	1.13	0.86	0.52	0.96	0.00
Q7Z408	CUB and sushi domain-containing protein 2 OS=Homo sapiens GN=CSMD2 PE=1 SV=2 - [CSMD2_HUMAN]	1	0.8	1.07	2.09	1.03	1.53	1.09	2.13	1.01	1.56	####	0.46	####	####	0.32	1.35	0.32	0.77	0.00
Q86YW5	Trem-like transcript 1 protein OS=Homo sapiens GN=TREML1 PE=1 SV=2 - [TRML1_HUMAN]	3	0.8	1.92	0.76	1.54	1.91	0.37	####	####	0.39	1.37	0.06	0.74	1.74	1.31	0.17	0.85	1.16	0.00
A6NM62	Leucine-rich repeat-containing protein 53 OS=Homo sapiens GN=LRRC53 PE=4 SV=2 - [LRC53_HUMAN]	1	0.8	1.69	1.28	1.51	####	0.26	####	0.04	####	1.54	1.16	1.30	####	2.28	1.88	2.14	0.41	0.01
Q8IVL1	Neuron navigator 2 OS=Homo sapiens GN=NAV2 PE=1 SV=3 - [NAV2_HUMAN]	1	0.8	####	####	0.67	####	####	####	0.67	####	0.21	0.08	0.97	0.29	2.66	2.51	3.52	2.68	0.02
A6NLC5	UPF0524 protein C3orf70 OS=Homo sapiens GN=C3orf70 PE=2 SV=1 - [CC070_HUMAN]	1	0.8	0.57	0.99	0.59	1.37	0.43	0.87	0.41	1.23	1.24	1.69	1.20	2.09	####	0.20	####	0.56	0.00
Q9Y287	Integral membrane protein 2B OS=Homo sapiens GN=ITM2B PE=1 SV=1 - [ITM2B_HUMAN]	2	0.8	1.64	2.01	0.96	0.39	1.30	1.71	0.65	0.06	0.55	1.79	0.11	####	0.92	1.30	0.30	####	0.00
Q4VNC1	Probable cation-transporting ATPase 13A4 OS=Homo sapiens GN=ATP13A4 PE=2 SV=3 - [AT134_HUMAN]	1	0.8	1.44	2.08	2.01	1.54	0.71	1.38	1.24	0.82	####	0.23	0.07	####	0.18	0.84	0.80	0.28	0.00
P01706	Ig lambda chain V-II region BOH OS=Homo sapiens PE=1 SV=1 - [LV203_HUMAN]	1	0.8	0.43	1.48	1.29	####	0.54	1.49	1.64	0.31	0.52	1.45	1.54	0.30	0.09	1.03	1.11	####	0.00
P41222	Prostaglandin-H2 D-isomerase OS=Homo sapiens GN=PTGDS PE=1 SV=1 - [PTGDS_HUMAN]	8	0.8	1.33	1.26	1.67	0.74	1.00	0.82	1.03	0.42	1.00	0.89	1.39	0.56	0.15	0.03	0.70	####	0.00
P02774	Vitamin D-binding protein OS=Homo sapiens GN=GC PE=1 SV=1 - [VTDB_HUMAN]	37	0.8	0.95	0.87	0.17	0.57	0.79	0.68	0.01	0.39	1.68	1.48	0.63	1.16	1.11	1.00	0.37	0.75	0.00
P06314	Ig kappa chain V-IV region B17 OS=Homo sapiens PE=2 SV=1 - [KV404_HUMAN]	1	0.8	1.11	0.73	0.50	####	1.37	0.95	0.49	####	2.01	1.85	1.48	0.63	1.21	0.79	0.57	####	0.00

P30990	Neurotensin/neuromedin N OS=Homo sapiens GN=NTS PE=1 SV=2 - [NEUT_HUMAN]	1	0.8	1.42	1.92	1.84	1.19	0.67	1.18	1.04	0.44	0.34	0.86	0.70	0.16	0.01	0.52	0.46	####	0.00
P83593	Ig kappa chain V-IV region STH (Fragment) OS=Homo sapiens PE=1 SV=1 - [KV405_HUMAN]	2	0.8	1.00	0.79	0.24	####	1.21	0.97	0.55	0.22	2.22	2.08	1.20	0.93	0.79	0.61	0.12	####	0.00
Q12789	General transcription factor 3C polypeptide 1 OS=Homo sapiens GN=GTF3C1 PE=1 SV=4 - [TF3C1_HUMAN]	1	0.8	0.53	0.64	0.56	1.19	0.97	1.09	0.96	1.63	0.30	0.43	0.27	1.01	0.51	0.62	0.58	1.16	0.00
Q15485	Ficolin-2 OS=Homo sapiens GN=FCN2 PE=1 SV=2 - [FCN2_HUMAN]	7	0.8	0.38	0.42	0.05	0.01	1.06	1.11	0.65	0.70	1.01	1.24	0.69	0.77	1.23	1.26	0.85	0.86	0.00
Q05BQ5	MBT domain-containing protein 1 OS=Homo sapiens GN=MBTD1 PE=1 SV=2 - [MBTD1_HUMAN]	1	0.8	2.47	1.55	1.49	0.40	2.23	1.33	1.21	0.16	0.39	####	####	####	1.93	1.03	1.00	####	0.02
P55056	Apolipoprotein C-IV OS=Homo sapiens GN=APOC4 PE=1 SV=1 - [APOC4_HUMAN]	7	0.8	1.31	1.23	1.47	0.66	0.92	0.72	1.22	0.33	0.50	0.27	0.62	####	1.06	0.59	1.24	0.17	0.00
P05181	Cytochrome P450 2E1 OS=Homo sapiens GN=CYP2E1 PE=1 SV=1 - [CP2E1_HUMAN]	1	0.8	1.66	0.69	1.45	####	1.69	0.54	1.47	####	1.98	1.12	1.81	####	1.12	####	0.73	####	0.01
Q9Y4D7	Plexin-D1 OS=Homo sapiens GN=PLXND1 PE=1 SV=3 - [PLXD1_HUMAN]	1	0.8	2.88	2.65	1.77	0.77	0.74	0.54	####	####	1.15	0.96	####	####	1.69	1.48	0.63	####	0.02
Q96L73	Histone-lysine N-methyltransferase, H3 lysine-36 and H4 lysine-20 specific OS=Homo sapiens GN=NSD1 PE=1 SV=1 - [NSD1_HUMAN]	2	0.8	1.35	1.02	0.03	0.20	1.06	0.90	####	0.07	0.90	0.55	####	####	2.37	2.04	1.08	1.23	0.00
P01137	Transforming growth factor beta-1 OS=Homo sapiens GN=TGFB1 PE=1 SV=2 - [TGFB1_HUMAN]	2	0.7	1.06	0.31	0.86	1.33	0.82	0.15	0.79	1.16	0.77	0.01	0.58	1.24	0.83	0.17	0.75	1.16	0.00
O43167	Zinc finger and BTB domain-containing protein 24 OS=Homo sapiens GN=ZBTB24 PE=1 SV=2 - [ZBT24_HUMAN]	1	0.7	0.83	####	####	0.26	0.63	####	####	0.06	2.60	1.59	1.24	2.08	1.84	0.80	0.58	1.26	0.01
O95810	Serum deprivation-response protein OS=Homo sapiens GN=SDPR PE=1 SV=3 - [SDPR_HUMAN]	1	0.7	2.15	1.66	2.64	2.14	####	####	0.23	####	1.30	0.84	1.73	1.34	####	####	0.28	####	0.02
P02753	Retinol-binding protein 4 OS=Homo sapiens GN=RBP4 PE=1 SV=3 - [RET4_HUMAN]	17	0.7	0.70	1.06	0.11	0.55	0.53	0.89	####	0.39	1.28	1.64	0.77	1.26	0.74	1.13	0.25	0.65	0.00
Q9HBH5	Retinol dehydrogenase 14 OS=Homo sapiens GN=RDH14 PE=1 SV=1 - [RDH14_HUMAN]	1	0.7	####	####	1.14	0.26	####	0.02	1.41	0.57	####	####	0.90	0.13	1.73	1.79	3.27	2.33	0.02
Q8N3D4	EH domain-binding protein 1-like protein 1 OS=Homo sapiens GN=EHBP1L1 PE=1 SV=2 - [EH1L1_HUMAN]	2	0.7	####	####	0.78	1.30	0.06	####	0.83	1.38	0.82	0.73	1.57	2.19	0.07	####	0.92	1.39	0.00
P42224	Signal transducer and activator of transcription 1-alpha/beta OS=Homo sapiens GN=STAT1 PE=1 SV=2 - [STAT1_HUMAN]	1	0.7	0.45	0.93	####	0.71	1.11	1.61	0.43	1.37	0.76	1.26	0.06	1.07	0.52	1.01	####	0.77	0.00
P12273	Prolactin-inducible protein OS=Homo sapiens GN=PIP PE=1 SV=1 - [PIP_HUMAN]	1	0.7	0.85	1.21	0.48	0.28	1.46	1.84	1.05	0.90	0.58	0.97	0.15	0.06	0.62	0.99	0.29	0.04	0.00
P01730	T-cell surface glycoprotein CD4 OS=Homo sapiens GN=CD4 PE=1 SV=1 - [CD4_HUMAN]	1	0.7	0.38	0.81	0.83	####	0.28	0.73	0.69	####	0.68	1.14	1.07	0.23	1.19	1.64	1.69	0.68	0.00
Q6JBY9	CapZ-interacting protein OS=Homo sapiens GN=RCSD1 PE=1 SV=1 - [CPZIP_HUMAN]	1	0.7	0.25	####	####	####	1.27	0.40	0.65	0.93	1.06	0.20	0.42	0.76	2.13	1.25	1.59	1.77	0.00
O00151	PDZ and LIM domain protein 1 OS=Homo sapiens GN=PDLIM1 PE=1 SV=4 - [PDLI1_HUMAN]	4	0.7	2.66	0.66	2.92	2.78	####	####	0.30	0.20	1.09	####	1.82	1.66	0.27	####	0.71	0.39	0.05
Q9HB07	UPF0160 protein MYG1, mitochondrial OS=Homo sapiens GN=C12orf10 PE=1 SV=2 - [MYG1_HUMAN]	1	0.7	0.66	1.44	0.99	1.97	####	0.61	0.09	1.12	0.06	0.87	0.33	1.42	####	0.72	0.29	1.22	0.00

Q9H8L6	Multimerin-2 OS=Homo sapiens GN=MMRN2 PE=1 SV=2 - [MMRN2_HUMAN]	1	0.7	1.37	0.47	1.96	0.79	1.17	0.29	1.72	0.60	0.17	####	0.70	####	1.03	0.14	1.66	0.45	0.00
A6NES4	Maestro heat-like repeat-containing protein family member 2A OS=Homo sapiens GN=MROH2A PE=4 SV=3 - [MRO2A_HUMAN]	1	0.7	0.39	1.67	1.34	0.67	0.03	1.32	0.93	0.31	####	0.99	0.58	0.01	0.23	1.52	1.22	0.50	0.00
P01610	Ig kappa chain V-I region WEA OS=Homo sapiens PE=1 SV=1 - [KV118_HUMAN]	2	0.7	0.54	0.73	1.31	0.36	0.24	0.49	1.03	0.06	0.55	0.66	1.28	0.52	0.72	0.88	1.34	0.63	0.00
P02654	Apolipoprotein C-I OS=Homo sapiens GN=APOC1 PE=1 SV=1 - [APOC1_HUMAN]	8	0.7	1.61	1.31	0.65	0.25	1.33	0.94	0.19	####	0.73	0.31	####	####	1.82	1.55	0.93	0.56	0.00
Q9UNN8	Endothelial protein C receptor OS=Homo sapiens GN=PROCR PE=1 SV=1 - [EPCR_HUMAN]	3	0.7	0.45	0.15	0.26	0.70	1.77	1.20	1.43	1.72	0.98	0.60	0.71	1.18	0.16	####	####	0.41	0.00
A1IGU5	Rho guanine nucleotide exchange factor 37 OS=Homo sapiens GN=ARHGEF37 PE=2 SV=2 - [ARH37_HUMAN]	2	0.7	####	####	####	####	1.84	1.14	1.65	1.45	1.83	0.06	0.71	1.05	1.73	####	0.71	0.90	0.01
Q86YV5	Tyrosine-protein kinase SgK223 OS=Homo sapiens GN=SGK223 PE=1 SV=4 - [SG223_HUMAN]	2	0.7	0.92	0.52	####	0.01	1.87	1.48	0.66	0.96	1.48	1.10	0.25	0.61	1.03	0.63	####	0.11	0.00
O95359	Transforming acidic coiled-coil-containing protein 2 OS=Homo sapiens GN=TACC2 PE=1 SV=3 - [TACC2_HUMAN]	2	0.7	1.22	0.70	0.31	0.23	1.24	0.74	0.30	0.26	0.79	0.30	####	####	1.95	1.44	1.09	0.95	0.00
P81605	Dermcidin OS=Homo sapiens GN=DCD PE=1 SV=2 - [DCD_HUMAN]	4	0.7	0.21	0.42	0.63	0.44	1.10	1.51	1.77	1.30	0.00	0.31	0.40	0.31	0.40	0.57	1.09	0.68	0.00
Q13790	Apolipoprotein F OS=Homo sapiens GN=APOF PE=1 SV=2 - [APOF_HUMAN]	8	0.7	1.03	1.26	1.00	0.45	1.03	1.26	0.97	0.43	0.94	0.96	0.57	####	0.50	0.62	0.43	####	0.00
Q8N9I0	Synaptotagmin-2 OS=Homo sapiens GN=SYT2 PE=1 SV=2 - [SYT2_HUMAN]	1	0.7	1.99	2.01	1.20	0.36	1.48	1.52	0.64	####	####	####	####	####	1.78	1.81	1.03	0.13	0.02
Q8N7M2	Zinc finger protein 283 OS=Homo sapiens GN=ZNF283 PE=2 SV=4 - [ZN283_HUMAN]	1	0.7	0.89	0.05	0.50	1.11	0.96	0.14	0.52	1.17	1.51	0.70	1.06	1.77	0.41	####	0.06	0.62	0.00
P24468	COUP transcription factor 2 OS=Homo sapiens GN=NR2F2 PE=1 SV=1 - [COT2_HUMAN]	1	0.7	0.93	1.12	0.07	0.34	0.76	0.96	####	0.17	1.04	1.26	0.13	0.50	1.27	1.47	0.46	0.68	0.00
Q9UQB3	Catenin delta-2 OS=Homo sapiens GN=CTNND2 PE=1 SV=3 - [CTND2_HUMAN]	2	0.7	1.68	1.50	0.50	0.05	1.13	0.96	####	####	1.46	1.30	0.23	####	1.44	1.27	0.31	####	0.00
P50851	Lipopolysaccharide-responsive and beige-like anchor protein OS=Homo sapiens GN=LRBA PE=1 SV=4 - [LRBA_HUMAN]	4	0.7	0.40	0.44	1.45	0.36	0.51	####	1.52	0.47	0.76	0.83	1.76	0.77	0.18	0.23	1.28	0.13	0.00
P24158	Myeloblastin OS=Homo sapiens GN=PRTN3 PE=1 SV=3 - [PRTN3_HUMAN]	2	0.7	1.60	1.43	1.85	1.25	0.66	0.40	1.13	0.28	0.40	####	0.74	####	0.45	0.28	0.90	0.02	0.00
Q9NZV5	Selenoprotein N OS=Homo sapiens GN=SEPN1 PE=1 SV=5 - [SELN_HUMAN]	1	0.7	2.17	1.46	0.87	0.25	1.56	0.87	0.23	####	1.07	0.39	####	####	1.82	1.12	0.57	####	0.01
P52848	Bifunctional heparan sulfate N-deacetylase/N-sulfotransferase 1 OS=Homo sapiens GN=NDST1 PE=1 SV=1 - [NDST1_HUMAN]	1	0.7	1.63	1.48	1.00	1.00	1.39	1.47	1.08	0.89	0.07	####	####	####	1.03	0.72	0.45	0.31	0.00
A6NM15	Putative COBW domain-containing protein 7 OS=Homo sapiens GN=CBWD7 PE=5 SV=3 - [CBWD7_HUMAN]	1	0.7	1.37	0.60	1.01	0.11	1.23	0.47	0.82	####	1.49	0.75	1.08	0.28	0.99	0.23	0.68	####	0.00
Q5VWQ0	Round spermatid basic protein 1 OS=Homo sapiens GN=RSBN1 PE=1 SV=2 - [RSBN1_HUMAN]	1	0.7	0.72	1.26	0.72	0.60	0.82	1.37	0.77	0.69	0.64	1.20	0.58	0.56	0.11	0.65	0.15	####	0.00
Q14558	Phosphoribosyl pyrophosphate synthase-associated protein 1 OS=Homo sapiens GN=PRPSAP1 PE=1 SV=2 - [KPRA_HUMAN]	1	0.7	2.35	1.60	0.32	####	2.30	1.57	0.25	####	1.47	0.75	####	####	2.24	1.51	0.35	####	0.04

O00187	Mannan-binding lectin serine protease 2 OS=Homo sapiens GN=MASP2 PE=1 SV=4 - [MASP2_HUMAN]	7	0.7	0.94	0.86	0.16	0.29	0.85	0.78	####	0.09	1.04	1.06	0.26	0.41	1.44	1.37	0.65	0.64	0.00
Q9H0J4	Glutamine-rich protein 2 OS=Homo sapiens GN=QRICH2 PE=1 SV=1 - [QRIC2_HUMAN]	1	0.7	0.05	1.07	0.52	0.15	0.96	2.00	1.39	1.06	0.81	1.87	1.23	0.96	####	0.29	####	####	0.00
Q9NZN3	EH domain-containing protein 3 OS=Homo sapiens GN=EHD3 PE=1 SV=2 - [EHD3_HUMAN]	1	0.7	2.41	1.38	2.93	2.80	####	####	0.12	0.03	0.57	####	1.03	1.01	0.16	####	0.72	0.54	0.05
P02652	Apolipoprotein A-II OS=Homo sapiens GN=APOA2 PE=1 SV=1 - [APOA2_HUMAN]	14	0.7	1.22	1.39	0.97	0.53	0.55	0.73	0.25	####	0.52	0.65	0.11	####	1.15	1.34	0.95	0.47	0.00
P25311	Zinc-alpha-2-glycoprotein OS=Homo sapiens GN=AZGP1 PE=1 SV=2 - [ZA2G_HUMAN]	24	0.7	0.79	0.60	0.81	0.55	0.96	0.82	0.90	0.74	0.51	0.29	0.29	0.24	0.87	0.68	0.86	0.65	0.00
Q9H3F6	BTB/POZ domain-containing adapter for CUL3-mediated RhoA degradation protein 3 OS=Homo sapiens GN=KCTD10 PE=1 SV=1 - [BACD3_HUMAN]	1	0.7	0.85	0.77	0.53	0.45	0.82	0.66	0.16	0.45	1.07	0.91	0.60	0.63	0.86	0.86	0.58	0.34	0.00
Q9UGM5	Fetuin-B OS=Homo sapiens GN=FETUB PE=1 SV=2 - [FETUB_HUMAN]	7	0.7	0.78	0.89	####	0.24	0.57	0.72	####	0.16	1.74	1.79	0.15	1.19	1.70	1.48	####	0.84	0.01
Q7Z698	Sprouty-related, EVH1 domain-containing protein 2 OS=Homo sapiens GN=SPRED2 PE=1 SV=2 - [SPRE2_HUMAN]	1	0.7	0.92	0.72	0.03	####	2.05	1.87	1.11	0.39	1.63	1.46	0.68	0.02	0.76	0.58	####	####	0.01
Q58EX7	Puratrophin-1 OS=Homo sapiens GN=PLEKHG4 PE=1 SV=1 - [PKHG4_HUMAN]	2	0.7	0.57	0.07	0.87	0.59	0.93	0.59	1.27	1.19	0.96	0.49	1.21	1.04	0.43	####	0.22	0.21	0.00
P24592	Insulin-like growth factor-binding protein 6 OS=Homo sapiens GN=IGFBP6 PE=1 SV=1 - [IBP6_HUMAN]	3	0.7	1.47	0.94	0.91	0.16	1.41	0.99	0.80	0.13	0.44	0.29	0.04	####	1.35	0.98	0.87	0.06	0.00
P52566	Rho GDP-dissociation inhibitor 2 OS=Homo sapiens GN=ARHGDIB PE=1 SV=3 - [GDIR2_HUMAN]	1	0.7	2.51	1.90	1.47	0.91	0.39	####	####	####	1.43	0.84	0.33	####	1.52	0.92	0.52	####	0.02
Q8IY37	Probable ATP-dependent RNA helicase DHX37 OS=Homo sapiens GN=DHX37 PE=1 SV=1 - [DHX37_HUMAN]	2	0.6	####	0.03	0.36	####	0.81	0.60	1.22	0.33	0.17	2.92	3.32	0.61	0.04	####	0.57	####	0.03
P49796	Regulator of G-protein signaling 3 OS=Homo sapiens GN=RGS3 PE=1 SV=2 - [RGS3_HUMAN]	1	0.6	####	####	####	0.80	1.05	2.05	1.41	2.53	0.87	1.58	1.40	2.44	####	####	####	0.07	0.05
Q9Y5M8	Signal recognition particle receptor subunit beta OS=Homo sapiens GN=SRPRB PE=1 SV=3 - [SRPRB_HUMAN]	1	0.6	1.19	1.37	0.88	0.40	0.73	0.92	0.38	####	1.02	1.22	0.65	0.28	0.57	0.75	0.30	####	0.00
Q14678	KN motif and ankyrin repeat domain-containing protein 1 OS=Homo sapiens GN=KANK1 PE=1 SV=3 - [KANK1_HUMAN]	2	0.6	0.66	0.84	1.20	0.69	0.43	0.89	1.08	0.47	0.19	0.40	0.68	0.05	0.46	0.83	1.20	0.23	0.00
Q8WUR7	UPF0235 protein C15orf40 OS=Homo sapiens GN=C15orf40 PE=1 SV=2 - [CO040_HUMAN]	1	0.6	1.14	0.60	0.66	####	0.82	0.29	0.29	####	1.42	0.90	0.87	0.18	1.50	0.96	1.05	0.20	0.00
P10911	Proto-oncogene DBL OS=Homo sapiens GN=MCF2 PE=1 SV=3 - [MCF2_HUMAN]	1	0.6	1.68	1.94	1.83	1.98	0.33	0.61	0.43	0.63	0.02	0.31	0.10	0.36	####	0.06	####	0.08	0.00
Q5VSY0	G kinase-anchoring protein 1 OS=Homo sapiens GN=GKAP1 PE=1 SV=2 - [GKAP1_HUMAN]	1	0.6	1.72	0.83	2.20	1.36	0.92	0.05	1.36	0.56	####	####	0.35	####	0.72	####	1.24	0.35	0.01
Q6SZW1	Sterile alpha and TIR motif-containing protein 1 OS=Homo sapiens GN=SARM1 PE=1 SV=1 - [SARM1_HUMAN]	1	0.6	0.17	0.30	1.43	####	0.08	0.22	1.29	####	0.62	0.77	1.81	0.47	0.46	0.59	1.75	0.25	0.00
A6NIZ1	Ras-related protein Rap-1b-like protein OS=Homo sapiens PE=2 SV=1 - [RP1BL_HUMAN]	3	0.6	2.71	1.01	2.47	1.75	0.19	####	0.73	####	1.25	####	1.37	0.59	0.39	####	0.63	####	0.04
Q96K76	Ubiquitin carboxyl-terminal hydrolase 47 OS=Homo sapiens GN=USP47 PE=1 SV=3 - [UBP47_HUMAN]	1	0.6	0.07	####	0.34	####	1.45	0.58	1.68	1.16	0.20	####	0.42	####	1.67	0.80	1.98	1.37	0.01

P29622	Kallistatin OS=Homo sapiens GN=SERPINA4 PE=1 SV=3 - [KAIN_HUMAN]	2	0.6	1.26	1.01	0.12	0.47	0.81	0.60	####	0.04	0.81	0.22	####	####	2.03	1.67	0.94	0.98	0.00
P14151	L-selectin OS=Homo sapiens GN=SELL PE=1 SV=2 - [LYAM1_HUMAN]	1	0.6	1.00	0.99	1.14	0.37	0.83	0.83	0.95	0.20	0.35	0.39	0.40	####	0.85	0.81	0.95	0.17	0.00
Q15828	Cystatin-M OS=Homo sapiens GN=CST6 PE=1 SV=1 - [CYTM_HUMAN]	2	0.6	0.74	0.67	1.07	0.88	0.55	0.35	1.02	0.53	0.63	0.62	1.29	0.50	0.14	0.14	0.49	0.26	0.00
O60343	TBC1 domain family member 4 OS=Homo sapiens GN=TBC1D4 PE=1 SV=2 - [TBCD4_HUMAN]	1	0.6	####	####	####	####	0.23	0.19	####	0.40	2.20	2.17	1.87	2.41	0.74	0.69	0.51	0.89	0.04
Q5T1V6	Probable ATP-dependent RNA helicase DDX59 OS=Homo sapiens GN=DDX59 PE=1 SV=1 - [DDX59_HUMAN]	1	0.6	0.09	1.06	0.86	####	####	0.85	0.58	####	0.71	1.70	1.41	0.62	0.15	1.13	0.96	0.01	0.00
P01614	Ig kappa chain V-II region Cum OS=Homo sapiens PE=1 SV=1 - [KV201_HUMAN]	1	0.6	0.37	1.12	0.33	0.10	0.17	0.95	0.10	####	1.57	2.57	1.64	1.56	####	0.48	####	####	0.02
O43597	Protein sprouty homolog 2 OS=Homo sapiens GN=SPRY2 PE=1 SV=1 - [SPY2_HUMAN]	1	0.6	####	0.01	0.23	####	0.92	1.45	1.62	0.82	0.55	1.09	1.23	0.49	0.26	0.78	1.03	0.14	0.00
Q12756	Kinesin-like protein KIF1A OS=Homo sapiens GN=KIF1A PE=1 SV=2 - [KIF1A_HUMAN]	1	0.6	1.09	1.74	0.71	0.12	1.26	1.92	0.84	0.28	0.04	0.72	####	####	0.68	1.34	0.34	####	0.01
B5MCY1	Tudor domain-containing protein 15 OS=Homo sapiens GN=TDRD15 PE=2 SV=1 - [TDR15_HUMAN]	1	0.6	####	####	####	0.65	1.95	1.19	1.66	2.94	0.62	####	0.32	1.66	0.16	####	####	1.14	0.05
Q8TCU4	Alstrom syndrome protein 1 OS=Homo sapiens GN=ALMS1 PE=1 SV=3 - [ALMS1_HUMAN]	2	0.6	1.59	1.32	0.83	0.18	0.86	0.85	0.03	####	0.92	0.02	####	####	1.69	1.38	0.98	0.26	0.01
O75901	Ras association domain-containing protein 9 OS=Homo sapiens GN=RASSF9 PE=2 SV=2 - [RASF9_HUMAN]	1	0.6	####	0.40	####	0.27	0.37	1.93	0.92	1.79	####	0.40	####	0.29	0.70	2.26	1.33	2.10	0.05
Q05084	Islet cell autoantigen 1 OS=Homo sapiens GN=ICA1 PE=1 SV=2 - [ICA69_HUMAN]	1	0.6	0.01	0.80	####	0.70	0.24	1.05	####	0.93	1.01	1.83	0.69	1.75	####	0.60	####	0.47	0.00
P49959	Double-strand break repair protein MRE11A OS=Homo sapiens GN=MRE11A PE=1 SV=3 - [MRE11_HUMAN]	1	0.6	1.19	1.95	0.56	0.86	0.65	1.43	####	0.32	####	0.05	####	####	1.37	2.15	0.78	1.03	0.04
Q9HBJ7	Ubiquitin carboxyl-terminal hydrolase 29 OS=Homo sapiens GN=USP29 PE=2 SV=1 - [UBP29_HUMAN]	2	0.6	0.67	0.79	0.31	0.18	0.82	0.96	0.42	0.34	0.81	0.96	0.39	0.37	0.67	0.81	0.36	0.18	0.00
P23471	Receptor-type tyrosine-protein phosphatase zeta OS=Homo sapiens GN=PTPRZ1 PE=1 SV=4 - [PTPRZ_HUMAN]	1	0.6	1.21	0.92	0.76	0.10	1.01	0.73	0.51	####	1.29	0.95	0.56	0.22	0.67	0.39	0.26	####	0.00
O75144	ICOS ligand OS=Homo sapiens GN=ICOSLG PE=1 SV=2 - [ICOSL_HUMAN]	1	0.6	0.45	0.66	0.89	0.31	0.67	0.65	0.87	0.34	0.45	0.60	0.73	0.26	0.41	0.53	0.89	0.26	0.00
Q2TAC2	Coiled-coil domain-containing protein 57 OS=Homo sapiens GN=CCDC57 PE=2 SV=2 - [CCD57_HUMAN]	1	0.6	0.28	0.71	1.08	1.03	####	####	0.24	0.23	####	0.44	0.73	0.79	0.51	0.95	1.36	1.25	0.00
Q14204	Cytoplasmic dynein 1 heavy chain 1 OS=Homo sapiens GN=DYNC1H1 PE=1 SV=5 - [DYHC1_HUMAN]	2	0.6	0.44	####	0.70	####	1.29	0.80	1.52	0.67	1.19	0.70	1.40	0.61	0.18	####	0.49	####	0.00
Q15047	Histone-lysine N-methyltransferase SETDB1 OS=Homo sapiens GN=SETDB1 PE=1 SV=1 - [SETB1_HUMAN]	1	0.6	1.50	1.56	1.03	0.50	0.73	0.62	0.10	####	0.68	0.60	####	####	1.01	1.16	0.50	####	0.00
Q9H2D6	TRIO and F-actin-binding protein OS=Homo sapiens GN=TRIOBP PE=1 SV=3 - [TARA_HUMAN]	1	0.6	0.07	####	0.18	0.37	0.85	0.75	0.93	1.16	####	####	####	0.25	1.08	0.97	1.24	1.38	0.00
P01597	Ig kappa chain V-I region DEE OS=Homo sapiens PE=1 SV=1 - [KV105_HUMAN]	3	0.6	0.63	0.66	0.71	0.45	1.23	1.07	1.30	1.04	0.71	0.78	0.69	0.45	####	####	####	####	0.00
P01034	Cystatin-C OS=Homo sapiens GN=CST3 PE=1 SV=1 - [CYTC_HUMAN]	5	0.5	0.40	0.86	0.92	0.68	####	0.50	0.59	0.22	0.67	1.00	1.14	0.59	####	0.44	0.60	0.29	0.00

O94832	Unconventional myosin-Id OS=Homo sapiens GN=MYO1D PE=1 SV=2 - [MYO1D_HUMAN]	2	0.5	1.26	0.76	####	0.48	1.22	0.72	####	0.45	1.01	0.53	####	0.28	1.43	0.73	0.07	0.65	0.00
Q8IYS5	Osteoclast-associated immunoglobulin-like receptor OS=Homo sapiens GN=OSCAR PE=2 SV=3 - [OSCAR_HUMAN]	1	0.5	1.06	0.98	1.14	2.12	0.42	0.37	0.46	1.48	####	####	####	0.15	0.57	0.51	0.70	1.63	0.03
Q9H4A3	Serine/threonine-protein kinase WNK1 OS=Homo sapiens GN=WNK1 PE=1 SV=2 - [WNK1_HUMAN]	2	0.5	0.54	0.77	0.85	0.53	0.22	0.41	0.41	0.13	0.69	0.89	0.44	0.22	0.53	0.72	0.80	0.43	0.00
Q2VY69	Zinc finger protein 284 OS=Homo sapiens GN=ZNF284 PE=2 SV=1 - [ZN284_HUMAN]	1	0.5	1.56	1.21	1.28	0.18	1.28	0.94	0.95	####	####	####	####	####	1.67	1.33	1.43	0.28	0.05
O94989	Rho guanine nucleotide exchange factor 15 OS=Homo sapiens GN=ARHGEF15 PE=1 SV=4 - [ARHGF_HUMAN]	1	0.5	1.15	0.67	0.83	0.45	0.97	0.51	0.61	0.27	1.78	1.33	1.40	1.12	####	####	####	####	0.02
Q6E0U4	Dermokine OS=Homo sapiens GN=DMKN PE=1 SV=3 - [DMKN_HUMAN]	1	0.5	1.10	0.15	0.87	0.16	1.10	0.34	0.84	0.33	1.71	0.81	1.43	1.10	0.10	####	####	####	0.01
P32456	Interferon-induced guanylate-binding protein 2 OS=Homo sapiens GN=GBP2 PE=1 SV=3 - [GBP2_HUMAN]	1	0.5	1.31	1.70	1.06	0.82	1.22	1.63	0.93	0.73	####	0.27	####	####	0.08	0.48	####	####	0.01
Q9NQ48	Leucine zipper transcription factor-like protein 1 OS=Homo sapiens GN=LZTFL1 PE=1 SV=1 - [LZTL1_HUMAN]	1	0.5	1.32	1.22	1.35	0.68	0.94	0.86	0.94	0.31	####	####	####	####	1.01	0.92	1.09	0.37	0.01
Q8TCS8	Polyribonucleotide nucleotidyltransferase 1, mitochondrial OS=Homo sapiens GN=PNPT1 PE=1 SV=2 - [PNPT1_HUMAN]	2	0.5	0.67	0.67	0.17	####	1.54	1.56	1.00	####	1.47	1.50	0.92	####	0.86	0.88	0.41	####	0.03
Q8IWZ8	SURP and G-patch domain-containing protein 1 OS=Homo sapiens GN=SUGP1 PE=1 SV=2 - [SUGP1_HUMAN]	1	0.5	1.01	0.82	1.32	0.28	0.90	0.73	1.17	0.17	####	####	####	####	1.39	1.21	1.74	0.64	0.03
P02671	Fibrinogen alpha chain OS=Homo sapiens GN=FGA PE=1 SV=2 - [FIBA_HUMAN]	25	0.5	1.22	0.97	1.23	1.08	0.30	0.12	0.25	0.20	0.74	0.61	0.51	0.46	0.17	0.05	0.34	0.13	0.00
P17931	Galectin-3 OS=Homo sapiens GN=LGALS3 PE=1 SV=5 - [LEG3_HUMAN]	1	0.5	0.72	0.50	0.63	0.62	0.58	0.38	0.45	0.48	0.39	0.20	0.24	0.33	0.80	0.60	0.76	0.69	0.00
Q6NUJ1	Proactivator polypeptide-like 1 OS=Homo sapiens GN=PSAPL1 PE=2 SV=2 - [SAPL1_HUMAN]	1	0.5	0.74	1.02	1.32	0.87	####	0.21	0.45	0.04	####	####	0.13	####	1.03	1.31	1.64	1.14	0.01
P00451	Coagulation factor VIII OS=Homo sapiens GN=F8 PE=1 SV=1 - [FA8_HUMAN]	2	0.5	1.98	0.37	1.53	0.08	1.02	####	0.53	####	1.54	####	1.03	####	1.49	####	1.09	####	0.03
P55103	Inhibin beta C chain OS=Homo sapiens GN=INHBC PE=1 SV=1 - [INHBC_HUMAN]	3	0.5	0.87	0.81	1.01	0.64	0.59	0.49	0.81	0.41	0.38	0.35	0.82	0.25	0.17	0.12	0.63	####	0.00
P01620	Ig kappa chain V-III region SIE OS=Homo sapiens PE=1 SV=1 - [KV302_HUMAN]	1	0.5	####	0.89	1.02	0.14	0.28	0.84	1.24	0.33	####	1.02	0.98	0.52	####	0.65	0.65	####	0.00
B2RXH8	Heterogeneous nuclear ribonucleoprotein C-like 2 OS=Homo sapiens GN=HNRNPCL2 PE=2 SV=1 - [HNRC2_HUMAN]	1	0.5	1.29	0.74	####	0.50	1.39	0.85	####	0.60	0.84	0.31	####	0.10	1.64	1.10	####	0.84	0.02
O60476	Mannosyl-oligosaccharide 1,2-alpha-mannosidase IB OS=Homo sapiens GN=MAN1A2 PE=1 SV=1 - [MA1A2_HUMAN]	1	0.5	1.34	1.41	1.07	1.68	0.90	0.99	0.59	1.24	####	####	####	####	0.41	0.49	0.18	0.74	0.03
P17936	Insulin-like growth factor-binding protein 3 OS=Homo sapiens GN=IGFBP3 PE=1 SV=2 - [IBP3_HUMAN]	12	0.5	0.51	0.84	0.91	0.49	0.58	0.85	1.06	0.59	####	####	####	####	0.52	0.87	1.03	0.49	0.00
Q9GZM8	Nuclear distribution protein nudE-like 1 OS=Homo sapiens GN=NDEL1 PE=1 SV=1 - [NDEL1_HUMAN]	1	0.5	0.89	0.95	####	0.20	0.88	0.95	####	0.19	0.98	0.69	0.57	0.87	0.57	0.64	####	####	0.00

P48741	Putative heat shock 70 kDa protein 7 OS=Homo sapiens GN=HSPA7 PE=5 SV=2 - [HSP77_HUMAN]	1	0.5	0.63	0.69	0.47	0.22	0.68	0.76	0.47	0.28	0.18	0.28	####	####	0.93	1.00	0.81	0.51	0.00
Q9BYX2	TBC1 domain family member 2A OS=Homo sapiens GN=TBC1D2 PE=1 SV=3 - [TBD2A_HUMAN]	1	0.5	####	####	0.81	####	0.47	0.23	1.25	0.52	1.02	1.04	1.63	1.09	####	####	0.79	####	0.01
Q9H4L7	SWI/SNF-related matrix-associated actin-dependent regulator of chromatin subfamily A containing DEAD/H box 1 OS=Homo sapiens GN=SMARCAD1 PE=1 SV=2 - [SMRCD_HUMAN]	1	0.5	0.36	0.78	0.73	0.32	0.29	0.72	0.62	0.25	0.79	1.23	1.10	0.80	####	0.11	0.09	####	0.00
P07477	Trypsin-1 OS=Homo sapiens GN=PRSS1 PE=1 SV=1 - [TRY1_HUMAN]	1	0.5	0.80	0.97	0.09	0.84	0.30	0.49	####	0.34	0.75	0.95	####	0.84	0.50	0.68	####	0.54	0.00
O95445	Apolipoprotein M OS=Homo sapiens GN=APOM PE=1 SV=2 - [APOM_HUMAN]	9	0.5	0.55	0.31	####	####	1.63	1.29	0.82	0.94	0.21	####	####	####	1.16	0.81	0.49	0.56	0.01
P49913	Cathelicidin antimicrobial peptide OS=Homo sapiens GN=CAMP PE=1 SV=1 - [CAMP_HUMAN]	6	0.5	1.16	1.19	1.08	0.51	0.53	0.79	0.60	0.08	0.31	0.49	0.38	####	0.06	0.43	0.33	####	0.00
Q5VST9	Obscurin OS=Homo sapiens GN=OBSCN PE=1 SV=3 - [OBSCN_HUMAN]	3	0.5	1.07	1.04	####	0.86	0.93	0.92	####	0.73	1.21	1.35	####	0.88	0.38	0.36	####	0.17	0.02
P0CG48	Polyubiquitin-C OS=Homo sapiens GN=UBC PE=1 SV=3 - [UBC_HUMAN]	5	0.5	0.83	0.85	2.13	0.86	####	####	0.91	####	0.12	0.07	1.14	0.14	####	0.03	1.19	0.02	0.02
Q92945	Far upstream element-binding protein 2 OS=Homo sapiens GN=KHSRP PE=1 SV=4 - [FUBP2_HUMAN]	1	0.5	0.36	1.24	####	0.44	0.15	1.04	####	0.23	0.94	1.84	0.48	1.07	####	0.71	####	####	0.01
Q13563	Polycystin-2 OS=Homo sapiens GN=PKD2 PE=1 SV=3 - [PKD2_HUMAN]	1	0.5	0.66	0.91	0.83	0.97	0.20	0.47	0.33	0.51	0.37	0.65	0.48	0.73	####	0.12	0.07	0.16	0.00
P01040	Cystatin-A OS=Homo sapiens GN=CSTA PE=1 SV=1 - [CYTA_HUMAN]	2	0.5	0.49	0.17	0.84	0.19	0.61	1.12	1.61	0.64	####	0.20	1.32	####	####	0.06	0.90	####	0.01
Q7Z460	CLIP-associating protein 1 OS=Homo sapiens GN=CLASP1 PE=1 SV=1 - [CLAP1_HUMAN]	1	0.5	1.02	1.01	0.58	0.35	0.60	0.61	0.12	####	0.58	0.60	0.08	####	0.72	0.72	0.32	0.04	0.00
P19022	Cadherin-2 OS=Homo sapiens GN=CDH2 PE=1 SV=4 - [CADH2_HUMAN]	2	0.5	####	1.14	1.35	0.34	1.00	1.05	1.17	0.20	0.22	0.13	####	0.66	####	0.59	0.07	####	0.00
O14791	Apolipoprotein L1 OS=Homo sapiens GN=APOL1 PE=1 SV=5 - [APOL1_HUMAN]	16	0.4	0.67	0.07	0.01	0.12	0.65	0.01	####	0.14	0.86	0.22	0.09	0.26	1.43	0.83	0.84	0.98	0.00
O76027	Annexin A9 OS=Homo sapiens GN=ANXA9 PE=1 SV=3 - [ANXA9_HUMAN]	1	0.4	0.73	0.81	1.31	0.64	0.65	0.76	1.20	0.57	####	####	0.30	####	0.03	0.12	0.66	####	0.00
P63027	Vesicle-associated membrane protein 2 OS=Homo sapiens GN=VAMP2 PE=1 SV=3 - [VAMP2_HUMAN]	1	0.4	0.86	1.21	0.82	0.20	0.83	1.20	0.75	0.18	0.27	0.65	0.18	####	0.13	0.49	0.14	####	0.00
O75128	Protein cordon-bleu OS=Homo sapiens GN=COBL PE=1 SV=2 - [COBL_HUMAN]	1	0.4	0.71	1.17	1.13	0.55	0.70	1.18	1.08	0.54	####	0.19	0.06	####	####	0.39	0.38	####	0.01
P05452	Tetranectin OS=Homo sapiens GN=CLEC3B PE=1 SV=3 - [TETN_HUMAN]	19	0.4	0.76	0.85	0.79	0.25	0.60	0.64	0.42	####	1.02	0.72	0.32	####	0.48	0.34	0.38	####	0.00
P22692	Insulin-like growth factor-binding protein 4 OS=Homo sapiens GN=IGFBP4 PE=1 SV=2 - [IBP4_HUMAN]	3	0.4	0.55	0.45	0.43	1.06	####	####	####	0.04	1.16	1.50	0.82	1.78	0.07	####	####	0.30	0.02
A6NK44	Glyoxalase domain-containing protein 5 OS=Homo sapiens GN=GLOD5 PE=1 SV=3 - [GLOD5_HUMAN]	2	0.4	####	0.37	####	####	1.08	1.11	0.44	####	0.50	0.80	####	####	0.94	1.04	0.93	0.78	0.01
P01709	Ig lambda chain V-II region MGC OS=Homo sapiens PE=1 SV=1 - [LV206_HUMAN]	2	0.4	0.09	0.47	0.37	####	0.42	0.85	0.60	0.22	0.48	0.99	0.58	0.39	0.38	0.01	0.66	0.29	0.00

Q9Y6R7	IgGFc-binding protein OS=Homo sapiens GN=FCGBP PE=1 SV=3 - [FCGBP_HUMAN]	4	0.4	0.63	0.28	0.01	0.33	0.63	0.41	0.19	0.56	0.71	0.57	0.42	0.80	0.51	0.08	0.11	0.47	0.00
O43149	Zinc finger ZZ-type and EF-hand domain-containing protein 1 OS=Homo sapiens GN=ZZEF1 PE=1 SV=6 - [ZZEF1_HUMAN]	1	0.4	1.41	0.65	0.67	####	1.26	0.70	0.48	####	0.73	0.24	0.04	####	1.54	0.81	0.87	####	0.05
O75525	KH domain-containing, RNA-binding, signal transduction-associated protein 3 OS=Homo sapiens GN=KHDRBS3 PE=1 SV=1 - [KHDR3_HUMAN]	1	0.4	0.26	1.01	0.74	0.45	0.54	1.30	0.98	0.73	####	####	####	####	0.27	1.03	0.79	0.45	0.02
P24387	Corticotropin-releasing factor-binding protein OS=Homo sapiens GN=CRHBP PE=1 SV=2 - [CRHBP_HUMAN]	2	0.4	0.41	0.48	0.17	0.21	0.31	0.31	0.07	####	0.58	0.69	0.42	0.15	0.91	0.74	0.61	0.73	0.00
Q9HBW1	Leucine-rich repeat-containing protein 4 OS=Homo sapiens GN=LRRC4 PE=1 SV=2 - [LRRC4_HUMAN]	1	0.4	0.53	0.88	0.73	0.47	0.06	0.43	0.22	0.00	####	0.36	0.13	####	0.55	0.92	0.80	0.48	0.00
Q86YV0	RAS protein activator like-3 OS=Homo sapiens GN=RASAL3 PE=1 SV=2 - [RASL3_HUMAN]	1	0.4	0.32	0.23	1.17	0.68	####	####	0.54	0.09	####	####	0.38	0.00	0.85	0.77	1.74	1.20	0.02
P35443	Thrombospondin-4 OS=Homo sapiens GN=THBS4 PE=1 SV=2 - [TSP4_HUMAN]	2	0.4	1.24	0.48	0.36	0.34	0.74	0.16	####	0.15	0.53	0.06	####	0.21	1.21	0.37	0.37	0.33	0.00
P02655	Apolipoprotein C-II OS=Homo sapiens GN=APOC2 PE=1 SV=1 - [APOC2_HUMAN]	8	0.4	0.80	0.89	0.80	0.38	0.48	0.56	0.45	0.03	####	####	####	####	0.75	0.84	0.82	0.39	0.00
P05090	Apolipoprotein D OS=Homo sapiens GN=APOD PE=1 SV=1 - [APOD_HUMAN]	14	0.4	0.68	1.00	0.58	0.43	0.44	0.79	0.26	0.23	0.40	0.64	####	0.16	0.27	0.51	0.08	0.00	0.00
Q92802	NEDD4-binding protein 2-like 2 OS=Homo sapiens GN=N4BP2L2 PE=1 SV=1 - [N42L2_HUMAN]	1	0.4	####	0.15	0.96	0.44	####	####	0.61	0.13	####	0.12	0.85	0.44	0.37	0.60	1.44	0.87	0.01
P19652	Alpha-1-acid glycoprotein 2 OS=Homo sapiens GN=ORM2 PE=1 SV=2 - [A1AG2_HUMAN]	11	0.4	1.01	0.78	0.89	0.10	0.86	0.73	0.70	####	####	####	####	####	1.04	0.89	0.94	0.18	0.03
Q6XPR3	Repetin OS=Homo sapiens GN=RPTN PE=1 SV=1 - [RPTN_HUMAN]	1	0.4	0.46	1.13	0.49	0.99	0.26	0.95	0.26	0.79	####	0.21	####	0.09	0.09	0.77	0.17	0.61	0.01
P22304	Iduronate 2-sulfatase OS=Homo sapiens GN=IDS PE=1 SV=1 - [IDS_HUMAN]	1	0.4	0.40	####	1.58	1.07	####	####	0.98	0.52	0.05	####	1.17	0.77	####	####	1.11	0.55	0.04
Q96J65	Multidrug resistance-associated protein 9 OS=Homo sapiens GN=ABCC12 PE=1 SV=2 - [MRP9_HUMAN]	2	0.4	0.42	####	0.51	0.27	1.12	0.14	1.16	0.96	0.53	####	0.56	0.42	0.54	####	0.67	0.38	0.01
P36402	Transcription factor 7 OS=Homo sapiens GN=TCF7 PE=1 SV=3 - [TCF7_HUMAN]	1	0.4	1.10	1.69	0.78	0.69	0.44	1.05	0.08	0.03	####	0.32	####	####	0.44	1.04	0.16	0.02	0.03
P22352	Glutathione peroxidase 3 OS=Homo sapiens GN=GPX3 PE=1 SV=2 - [GPX3_HUMAN]	10	0.4	1.05	0.90	0.33	0.22	0.75	0.64	####	####	1.21	0.91	0.11	0.17	0.42	0.33	####	####	0.01
P11169	Solute carrier family 2, facilitated glucose transporter member 3 OS=Homo sapiens GN=SLC2A3 PE=1 SV=1 - [GTR3_HUMAN]	1	0.4	####	####	####	0.90	####	0.00	####	0.93	0.50	1.07	0.74	2.03	####	0.40	0.20	1.33	0.05
P42566	Epidermal growth factor receptor substrate 15 OS=Homo sapiens GN=EPS15 PE=1 SV=2 - [EPS15_HUMAN]	1	0.4	0.46	0.51	0.04	####	0.75	0.97	0.44	0.21	0.39	0.56	0.22	####	0.73	0.90	0.45	0.13	0.00
Q96NE9	FERM domain-containing protein 6 OS=Homo sapiens GN=FRMD6 PE=1 SV=1 - [FRMD6_HUMAN]	1	0.4	0.46	0.23	1.14	0.16	0.12	####	0.76	####	0.46	0.26	1.08	0.22	0.32	0.11	1.04	0.02	0.00
P05165	Propionyl-CoA carboxylase alpha chain, mitochondrial OS=Homo sapiens GN=PCCA PE=1 SV=4 - [PCCA_HUMAN]	1	0.4	0.75	1.19	####	0.05	0.61	1.03	####	0.04	####	1.24	0.54	####	0.30	1.16	####	####	0.02

Q9Y2H9	Microtubule-associated serine/threonine-protein kinase 1 OS=Homo sapiens GN=MAST1 PE=1 SV=2 - [MAST1_HUMAN]	2	0.4	0.86	0.57	0.40	0.91	0.86	0.58	0.36	0.90	####	####	####	####	0.76	0.48	0.34	0.80	0.02
P04208	Ig lambda chain V-I region WAH OS=Homo sapiens PE=1 SV=1 - [LV106_HUMAN]	4	0.4	0.29	0.31	0.83	0.51	####	####	0.36	0.11	0.33	0.36	0.72	0.61	0.28	0.28	0.73	0.44	0.00
Q12907	Vesicular integral-membrane protein VIP36 OS=Homo sapiens GN=LMAN2 PE=1 SV=1 - [LMAN2_HUMAN]	1	0.4	0.44	0.98	0.54	0.54	0.11	0.55	0.14	0.06	####	0.28	####	####	0.52	1.02	0.67	0.56	0.00
Q9UHI5	Large neutral amino acids transporter small subunit 2 OS=Homo sapiens GN=SLC7A8 PE=1 SV=1 - [LAT2_HUMAN]	1	0.4	1.28	0.91	0.72	0.35	0.68	0.33	0.07	####	0.04	####	####	####	1.32	0.97	0.81	0.39	0.04
P05160	Coagulation factor XIII B chain OS=Homo sapiens GN=F13B PE=1 SV=3 - [F13B_HUMAN]	2	0.4	0.82	0.28	####	0.20	0.30	####	####	0.20	1.71	1.37	0.77	1.23	0.35	####	####	0.16	0.04
Q9UL68	Myelin transcription factor 1-like protein OS=Homo sapiens GN=MYT1L PE=2 SV=3 - [MYT1L_HUMAN]	2	0.4	####	0.27	0.30	0.71	0.24	0.13	0.57	1.04	0.01	####	####	0.83	0.18	0.21	0.60	0.96	0.00
P02775	Platelet basic protein OS=Homo sapiens GN=PPBP PE=1 SV=3 - [CXCL7_HUMAN]	8	0.4	0.22	0.04	0.20	0.93	0.03	####	####	0.69	0.30	0.20	0.21	1.12	0.35	0.20	0.37	1.06	0.00
P59665	Neutrophil defensin 1 OS=Homo sapiens GN=DEFA1 PE=1 SV=1 - [DEF1_HUMAN]	3	0.4	0.47	0.13	0.48	####	0.30	####	0.17	####	1.30	0.94	1.18	0.75	0.31	####	0.32	####	0.01
Q14123	Calcium/calmodulin-dependent 3',5'-cyclic nucleotide phosphodiesterase 1C OS=Homo sapiens GN=PDE1C PE=1 SV=1 - [PDE1C_HUMAN]	2	0.4	####	0.57	0.38	0.21	0.05	0.74	0.49	0.37	####	0.26	####	####	0.44	1.12	0.96	0.74	0.00
Q9UBR2	Cathepsin Z OS=Homo sapiens GN=CTSZ PE=1 SV=1 - [CATZ_HUMAN]	2	0.4	0.26	0.78	0.99	1.03	####	0.25	0.52	0.48	####	0.12	0.17	0.41	####	0.38	0.60	0.61	0.00
Q15021	Condensin complex subunit 1 OS=Homo sapiens GN=NCAPD2 PE=1 SV=3 - [CND1_HUMAN]	1	0.4	0.59	0.38	0.50	0.86	0.05	####	####	0.33	0.69	0.51	0.56	1.01	0.10	####	0.06	0.36	0.00
P01033	Metalloproteinase inhibitor 1 OS=Homo sapiens GN=TIMP1 PE=1 SV=1 - [TIMP1_HUMAN]	3	0.4	0.27	0.38	####	0.24	0.02	0.29	####	0.13	1.21	1.28	0.66	1.20	0.24	0.26	####	0.22	0.01
P06744	Glucose-6-phosphate isomerase OS=Homo sapiens GN=GPI PE=1 SV=4 - [G6PI_HUMAN]	2	0.4	0.30	0.54	0.43	0.73	1.15	0.87	1.42	0.91	0.44	0.10	0.69	0.00	####	####	####	####	0.04
P01613	Ig kappa chain V-I region Ni OS=Homo sapiens PE=1 SV=1 - [KV121_HUMAN]	1	0.4	0.17	0.28	0.38	####	0.88	0.82	0.89	0.40	0.70	0.65	0.80	0.44	####	0.01	0.11	####	0.01
Q9ULI3	Protein HEG homolog 1 OS=Homo sapiens GN=HEG1 PE=1 SV=3 - [HEG1_HUMAN]	6	0.4	0.74	####	1.03	####	0.76	####	1.00	####	0.52	####	0.69	####	1.15	0.16	1.33	0.19	0.04
O76096	Cystatin-F OS=Homo sapiens GN=CST7 PE=1 SV=1 - [CYTF_HUMAN]	1	0.3	0.65	0.91	0.41	0.33	0.59	0.88	0.32	0.28	####	0.19	####	####	0.53	0.81	0.34	0.20	0.00
O94856	Neurofascin OS=Homo sapiens GN=NFASC PE=1 SV=4 - [NFASC_HUMAN]	1	0.3	0.65	0.64	1.08	0.30	0.41	0.42	0.80	0.06	0.30	0.31	0.67	####	####	####	0.43	####	0.00
A2RU48	Single-pass membrane and coiled-coil domain-containing protein 3 OS=Homo sapiens GN=SMCO3 PE=2 SV=1 - [SMCO3_HUMAN]	1	0.3	0.76	0.10	1.05	0.06	1.00	0.49	1.25	0.67	0.35	####	0.58	0.06	####	####	0.30	####	0.02
P04179	Superoxide dismutase [Mn], mitochondrial OS=Homo sapiens GN=SOD2 PE=1 SV=2 - [SODM_HUMAN]	4	0.3	0.76	0.65	0.56	0.35	0.51	0.59	0.26	0.10	####	####	####	####	0.85	0.91	0.53	0.53	0.01
P04207	Ig kappa chain V-III region CLL OS=Homo sapiens PE=1 SV=2 - [KV308_HUMAN]	2	0.3	0.49	0.09	0.50	####	0.81	0.42	0.89	0.41	1.19	0.47	0.97	0.48	0.06	####	0.08	####	0.03
P06887	Ig lambda chain V-I region MEM OS=Homo sapiens PE=1 SV=1 - [LV108_HUMAN]	1	0.3	0.15	0.78	0.88	####	####	0.67	0.74	####	0.25	0.84	1.22	0.70	####	####	0.35	####	0.02

Q13332	Receptor-type tyrosine-protein phosphatase S OS=Homo sapiens GN=PTPRS PE=1 SV=3 - [PTPRS_HUMAN]	1	0.3	0.47	0.64	0.89	0.24	0.37	0.52	0.73	0.11	####	0.12	0.31	####	0.13	0.35	0.59	####	0.00
P50552	Vasodilator-stimulated phosphoprotein OS=Homo sapiens GN=VASP PE=1 SV=3 - [VASP_HUMAN]	1	0.3	1.47	0.35	1.22	0.95	0.71	####	0.20	0.05	0.45	####	0.30	0.14	0.40	####	0.22	####	0.04
P55895	V(D)J recombination-activating protein 2 OS=Homo sapiens GN=RAG2 PE=1 SV=1 - [RAG2_HUMAN]	1	0.3	0.74	0.15	0.61	0.03	0.94	0.41	0.78	0.24	0.88	0.09	0.58	0.11	0.31	####	####	####	0.01
O60888	Protein CutA OS=Homo sapiens GN=CUTA PE=1 SV=2 - [CUTA_HUMAN]	1	0.3	1.34	1.09	0.88	0.40	0.35	0.14	####	####	0.87	0.59	0.12	####	0.47	0.15	####	####	0.04
P48634	Protein PRRC2A OS=Homo sapiens GN=PRRC2A PE=1 SV=3 - [PRC2A_HUMAN]	3	0.3	0.98	0.27	####	0.81	0.02	0.08	####	0.57	0.39	0.46	0.28	0.99	0.52	####	####	0.34	0.01
Q9Y279	V-set and immunoglobulin domain-containing protein 4 OS=Homo sapiens GN=VSIG4 PE=1 SV=1 - [VSIG4_HUMAN]	1	0.3	0.13	0.41	0.41	####	0.30	0.58	0.53	0.15	####	0.09	0.00	####	0.62	0.90	0.93	0.46	0.00
Q8IWE5	Pleckstrin homology domain-containing family M member 2 OS=Homo sapiens GN=PLEKHM2 PE=1 SV=2 - [PKHM2_HUMAN]	1	0.3	####	####	0.40	0.05	0.72	0.23	1.14	0.83	0.67	0.19	1.07	0.83	####	####	0.33	####	0.04
Q9UMX5	Neudesin OS=Homo sapiens GN=NENF PE=1 SV=1 - [NENF_HUMAN]	2	0.3	0.69	0.42	1.02	0.51	0.20	####	0.49	0.03	####	####	0.23	####	0.53	0.27	0.91	0.35	0.01
P02749	Beta-2-glycoprotein 1 OS=Homo sapiens GN=APOH PE=1 SV=3 - [APOH_HUMAN]	19	0.3	0.25	0.61	0.43	0.31	0.25	0.62	0.42	0.29	0.14	0.53	0.04	0.09	####	0.36	0.39	0.16	0.00
O75179	Ankyrin repeat domain-containing protein 17 OS=Homo sapiens GN=ANKRD17 PE=1 SV=3 - [ANR17_HUMAN]	2	0.3	0.69	0.45	0.16	####	0.50	0.28	####	####	1.03	0.82	0.45	0.07	0.75	0.53	0.27	####	0.01
P49815	Tuberin OS=Homo sapiens GN=TSC2 PE=1 SV=2 - [TSC2_HUMAN]	1	0.3	0.56	####	0.59	####	0.61	####	0.60	0.01	0.78	0.01	0.75	0.22	0.61	####	0.68	0.00	0.01
P02787	Serotransferrin OS=Homo sapiens GN=TF PE=1 SV=3 - [TRFE_HUMAN]	55	0.3	0.45	0.43	0.01	0.52	0.30	0.32	####	0.40	0.39	0.34	####	0.48	0.46	0.38	0.05	0.55	0.00
P09486	SPARC OS=Homo sapiens GN=SPARC PE=1 SV=1 - [SPRC_HUMAN]	9	0.3	0.27	0.18	0.53	0.87	####	####	0.10	0.58	0.11	####	0.04	0.65	0.21	0.15	0.58	0.89	0.00
Q5VTL8	Pre-mRNA-splicing factor 38B OS=Homo sapiens GN=PRPF38B PE=1 SV=1 - [PR38B_HUMAN]	1	0.3	0.59	0.49	####	0.40	0.81	0.73	####	0.62	0.35	0.27	####	0.21	0.65	0.56	####	0.45	0.01
P02790	Hemopexin OS=Homo sapiens GN=HPX PE=1 SV=2 - [HEMO_HUMAN]	35	0.3	0.32	0.26	####	0.14	0.35	0.33	####	0.22	0.69	0.69	####	0.49	0.54	0.50	0.09	0.38	0.00
Q68EN5	Uncharacterized protein KIAA0895-like OS=Homo sapiens GN=KIAA0895L PE=2 SV=1 - [K895L_HUMAN]	1	0.3	0.02	0.13	0.60	0.18	0.30	0.43	0.83	0.46	0.31	0.45	0.83	0.52	####	####	0.30	####	0.00
Q9UJ14	Gamma-glutamyltransferase 7 OS=Homo sapiens GN=GGT7 PE=1 SV=2 - [GGT7_HUMAN]	1	0.3	0.92	0.69	0.79	1.32	####	####	####	0.34	0.06	####	####	0.50	0.21	####	0.12	0.60	0.02
O75636	Ficolin-3 OS=Homo sapiens GN=FCN3 PE=1 SV=2 - [FCN3_HUMAN]	8	0.3	0.47	0.14	0.08	####	0.56	0.18	####	####	1.27	0.78	0.87	0.27	0.85	0.41	0.29	####	0.04
Q14624	Inter-alpha-trypsin inhibitor heavy chain H4 OS=Homo sapiens GN=ITIH4 PE=1 SV=4 - [ITIH4_HUMAN]	45	0.3	0.41	0.62	0.05	####	0.47	0.68	0.07	####	0.55	0.67	####	####	0.62	0.90	0.32	0.07	0.01
Q13635	Protein patched homolog 1 OS=Homo sapiens GN=PTCH1 PE=1 SV=2 - [PTC1_HUMAN]	1	0.3	0.07	####	####	0.08	0.31	0.16	0.06	0.32	####	####	####	0.02	1.17	1.01	1.00	1.17	0.04
Q8WU39	Marginal zone B- and B1-cell-specific protein OS=Homo sapiens GN=MZB1 PE=1 SV=1 - [MZB1_HUMAN]	1	0.3	0.64	0.79	1.36	0.81	####	####	0.29	####	####	0.16	0.65	0.20	####	0.02	0.63	0.02	0.03

P20933	N(4)-(beta-N-acetylglucosaminyl)-L-asparaginase OS=Homo sapiens GN=AGA PE=1 SV=2 - [ASPG_HUMAN]	1	0.3	0.88	0.81	1.09	0.92	0.14	0.01	0.30	0.30	0.13	####	0.37	0.12	####	####	0.17	####	0.02
P01603	Ig kappa chain V-I region Ka OS=Homo sapiens PE=1 SV=1 - [KV111_HUMAN]	2	0.3	0.12	0.10	0.31	0.00	0.29	0.48	0.86	0.45	0.44	0.61	0.89	0.76	####	####	0.07	####	0.01
Q9Y446	Plakophilin-3 OS=Homo sapiens GN=PKP3 PE=1 SV=1 - [PKP3_HUMAN]	1	0.3	0.45	0.99	0.61	0.34	0.19	0.75	0.31	0.08	####	0.40	####	####	0.04	0.60	0.25	####	0.01
P02768	Serum albumin OS=Homo sapiens GN=ALB PE=1 SV=2 - [ALBU_HUMAN]	81	0.3	0.22	0.52	####	0.16	0.36	0.66	0.11	0.30	0.48	0.74	0.00	0.36	0.09	0.40	####	0.09	0.00
Q14142	Tripartite motif-containing protein 14 OS=Homo sapiens GN=TRIM14 PE=2 SV=2 - [TRI14_HUMAN]	1	0.3	0.99	####	0.47	0.52	0.90	####	0.34	0.43	0.28	####	####	####	1.03	####	0.56	0.56	0.05
P02766	Transthyretin OS=Homo sapiens GN=TTR PE=1 SV=1 - [TTHY_HUMAN]	14	0.3	0.31	0.67	0.27	0.25	0.32	0.67	0.23	0.23	####	0.12	####	####	0.43	0.81	0.41	0.39	0.00
P06311	Ig kappa chain V-III region IARC/BL41 OS=Homo sapiens PE=1 SV=1 - [KV311_HUMAN]	2	0.3	0.47	0.22	0.44	####	0.54	0.40	0.67	0.20	0.77	####	0.30	0.04	0.38	0.24	0.34	####	0.00
P04211	Ig lambda chain V region 4A OS=Homo sapiens PE=4 SV=1 - [LV001_HUMAN]	2	0.3	0.49	0.71	0.81	0.30	0.16	0.54	0.32	####	0.22	0.38	0.25	0.11	####	0.12	0.17	####	0.00
Q96TA1	Niban-like protein 1 OS=Homo sapiens GN=FAM129B PE=1 SV=3 - [NIBL1_HUMAN]	1	0.3	0.46	0.67	0.53	0.02	0.23	0.45	0.26	####	0.25	0.49	0.27	####	0.22	0.43	0.33	####	0.00
P08922	Proto-oncogene tyrosine-protein kinase ROS OS=Homo sapiens GN=ROS1 PE=1 SV=3 - [ROS1_HUMAN]	1	-0.3	####	0.29	####	0.40	####	0.02	####	0.13	####	####	####	0.04	####	####	####	####	0.02
P01780	Ig heavy chain V-III region JON OS=Homo sapiens PE=1 SV=1 - [HV319_HUMAN]	1	-0.3	####	0.02	####	####	####	0.08	0.08	0.10	####	####	####	0.01	####	####	0.06	####	0.02
P0CG06	Ig lambda-3 chain C regions OS=Homo sapiens GN=IGLC3 PE=1 SV=1 - [LAC3_HUMAN]	1	-0.3	####	####	0.02	0.36	####	####	0.05	0.52	####	####	####	####	####	####	####	0.25	0.03
Q08380	Galectin-3-binding protein OS=Homo sapiens GN=LGALS3BP PE=1 SV=1 - [LG3BP_HUMAN]	7	-0.3	####	####	####	####	####	####	####	####	0.20	####	####	####	0.47	0.04	0.22	0.38	0.04
Q6PIF6	Unconventional myosin-VIIb OS=Homo sapiens GN=MYO7B PE=1 SV=2 - [MYO7B_HUMAN]	1	-0.3	####	####	0.20	####	####	####	####	####	####	####	####	####	####	####	0.48	####	0.00
P13987	CD59 glycoprotein OS=Homo sapiens GN=CD59 PE=1 SV=1 - [CD59_HUMAN]	1	-0.3	####	####	0.53	####	####	####	0.20	####	####	####	####	####	####	####	0.40	####	0.03
P12259	Coagulation factor V OS=Homo sapiens GN=F5 PE=1 SV=4 - [FA5_HUMAN]	29	-0.3	####	####	####	####	####	####	####	####	####	####	####	####	0.30	0.38	0.18	####	0.01
Q8NFL0	UDP-GlcNAc:betaGal beta-1,3-N-acetylglucosaminyltransferase 7 OS=Homo sapiens GN=B3GNT7 PE=2 SV=1 - [B3GN7_HUMAN]	1	-0.3	####	####	0.11	####	####	####	0.28	####	####	####	####	####	####	####	0.15	####	0.00
Q8NEZ4	Histone-lysine N-methyltransferase 2C OS=Homo sapiens GN=KMT2C PE=1 SV=3 - [KMT2C_HUMAN]	1	-0.3	####	0.07	####	0.29	####	####	####	####	####	####	0.34	####	####	####	####	0.04	0.01
P01762	Ig heavy chain V-III region TRO OS=Homo sapiens PE=1 SV=1 - [HV301_HUMAN]	2	-0.3	####	####	####	####	####	####	####	####	####	####	####	####	####	####	0.00	####	0.00
O95613	Pericentrin OS=Homo sapiens GN=PCNT PE=1 SV=4 - [PCNT_HUMAN]	1	-0.3	####	####	####	####	0.26	0.02	####	####	0.68	0.45	0.15	####	####	####	####	####	0.04
P01042	Kininogen-1 OS=Homo sapiens GN=KNG1 PE=1 SV=2 - [KNG1_HUMAN]	26	-0.3	####	####	####	####	####	####	####	####	####	####	####	####	0.45	0.44	####	0.11	0.01
Q8IUE1	Homeobox protein TGIF2LX OS=Homo sapiens GN=TGIF2LX PE=1 SV=1 - [TF2LX_HUMAN]	1	-0.3	####	####	####	0.05	####	0.11	####	0.48	####	####	####	0.02	####	####	####	####	0.00

Q5H9L2	Transcription elongation factor A protein-like 5 OS=Homo sapiens GN=TCEAL5 PE=1 SV=1 - [TCAL5_HUMAN]	2	-0.3	####	####	####	####	####	####	0.02	####	####	####	####	####	####	0.41	0.29	####	0.01
P54278	Mismatch repair endonuclease PMS2 OS=Homo sapiens GN=PMS2 PE=1 SV=2 - [PMS2_HUMAN]	1	-0.3	####	####	####	####	####	0.01	####	####	####	0.01	####	####	####	####	####	####	0.00
O14929	Histone acetyltransferase type B catalytic subunit OS=Homo sapiens GN=HAT1 PE=1 SV=1 - [HAT1_HUMAN]	1	-0.3	0.54	####	0.36	####	0.50	####	0.28	####	####	####	####	####	####	####	####	####	0.05
P01594	Ig kappa chain V-I region AU OS=Homo sapiens PE=1 SV=1 - [KV102_HUMAN]	1	-0.3	####	####	####	####	0.75	0.66	0.30	0.22	####	####	####	####	####	####	####	####	0.04
Q8NBJ4	Golgi membrane protein 1 OS=Homo sapiens GN=GOLM1 PE=1 SV=1 - [GOLM1_HUMAN]	1	-0.3	0.08	####	####	####	####	####	####	####	####	####	####	####	####	####	####	####	0.00
Q14766	Latent-transforming growth factor beta-binding protein 1 OS=Homo sapiens GN=LTBP1 PE=1 SV=4 - [LTBP1_HUMAN]	3	-0.3	####	####	####	0.12	####	####	####	####	####	####	####	0.42	####	0.17	####	0.74	0.02
Q7Z628	Neuroepithelial cell-transforming gene 1 protein OS=Homo sapiens GN=NET1 PE=1 SV=1 - [ARHG8_HUMAN]	1	-0.3	####	0.01	####	####	####	####	####	0.15	####	####	####	0.65	####	####	####	####	0.01
Q75QN2	Integrator complex subunit 8 OS=Homo sapiens GN=INTS8 PE=1 SV=1 - [INT8_HUMAN]	1	-0.3	####	####	####	0.51	####	####	####	0.09	####	####	####	0.01	0.30	####	####	0.85	0.04
Q15678	Tyrosine-protein phosphatase non-receptor type 14 OS=Homo sapiens GN=PTPN14 PE=1 SV=2 - [PTN14_HUMAN]	1	-0.4	####	0.19	####	####	####	####	####	####	####	####	####	####	####	0.53	0.18	0.13	0.02
Q9Y6H5	Synphilin-1 OS=Homo sapiens GN=SNCAIP PE=1 SV=2 - [SNCAP_HUMAN]	1	-0.4	0.16	0.13	0.01	0.35	0.13	0.12	####	0.32	####	####	####	####	####	####	####	####	0.04
Q14524	Sodium channel protein type 5 subunit alpha OS=Homo sapiens GN=SCN5A PE=1 SV=2 - [SCN5A_HUMAN]	1	-0.4	0.17	####	####	####	0.50	####	####	####	0.12	####	####	####	0.35	####	####	####	0.01
P14314	Glucosidase 2 subunit beta OS=Homo sapiens GN=PRKCSH PE=1 SV=2 - [GLU2B_HUMAN]	1	-0.4	####	####	####	####	####	####	####	0.05	####	####	0.09	0.68	####	####	####	0.34	0.01
O43768	Alpha-endosulfine OS=Homo sapiens GN=ENSA PE=1 SV=1 - [ENSA_HUMAN]	1	-0.4	####	####	####	####	####	####	0.22	0.19	####	####	0.17	####	####	####	####	####	0.00
Q99741	Cell division control protein 6 homolog OS=Homo sapiens GN=CDC6 PE=1 SV=1 - [CDC6_HUMAN]	3	-0.4	####	####	####	0.24	####	0.07	0.61	0.46	0.26	####	####	####	####	####	####	####	0.03
Q15149	Plectin OS=Homo sapiens GN=PLEC PE=1 SV=3 - [PLEC_HUMAN]	2	-0.4	0.18	0.22	0.42	0.38	####	####	####	####	####	####	####	####	####	####	0.15	0.05	0.04
P04209	Ig lambda chain V-II region NIG-84 OS=Homo sapiens PE=1 SV=1 - [LV211_HUMAN]	2	-0.4	####	####	0.37	####	0.09	####	0.33	####	####	####	0.28	####	####	####	####	####	0.04
Q9P2S5	WD repeat-containing protein WRAP73 OS=Homo sapiens GN=WRAP73 PE=2 SV=1 - [WRP73_HUMAN]	1	-0.4	####	####	####	####	0.31	####	0.55	0.43	####	####	####	####	####	####	####	####	0.01
P00740	Coagulation factor IX OS=Homo sapiens GN=F9 PE=1 SV=2 - [FA9_HUMAN]	11	-0.4	####	0.14	####	0.13	####	####	####	####	####	####	####	####	####	0.19	####	0.09	0.00
Q9UKV5	E3 ubiquitin-protein ligase AMFR OS=Homo sapiens GN=AMFR PE=1 SV=2 - [AMFR_HUMAN]	1	-0.4	0.21	0.20	####	0.10	0.24	0.25	####	0.13	0.06	0.07	####	####	####	####	####	####	0.04
Q2TAL5	Smoothelin-like protein 2 OS=Homo sapiens GN=SMTNL2 PE=2 SV=1 - [SMTL2_HUMAN]	1	-0.4	####	####	0.25	####	####	0.45	0.68	####	####	0.01	0.21	####	####	####	####	####	0.03
P04406	Glyceraldehyde-3-phosphate dehydrogenase OS=Homo sapiens GN=GAPDH PE=1 SV=3 - [G3P_HUMAN]	4	-0.4	####	####	####	####	####	####	####	####	0.14	0.13	0.36	0.15	####	####	####	####	0.00

Q8IVF7	Formin-like protein 3 OS=Homo sapiens GN=FMNL3 PE=1 SV=3 - [FMNL3_HUMAN]	1	-0.4	0.40	0.27	####	0.13	####	####	####	####	####	####	####	####	0.32	0.38	####	0.18	0.04
P02747	Complement C1q subcomponent subunit C OS=Homo sapiens GN=C1QC PE=1 SV=3 - [C1QC_HUMAN]	6	-0.4	####	####	####	####	0.10	####	####	0.07	####	####	####	####	####	####	####	####	0.00
Q9C0D6	FH2 domain-containing protein 1 OS=Homo sapiens GN=FHDC1 PE=1 SV=2 - [FHDC1_HUMAN]	1	-0.4	####	####	0.51	####	####	####	0.60	0.07	####	####	0.23	####	####	####	####	####	0.03
Q9UPP5	Uncharacterized protein KIAA1107 OS=Homo sapiens GN=KIAA1107 PE=1 SV=2 - [K1107_HUMAN]	2	-0.4	0.14	####	####	####	0.74	####	####	####	0.38	####	####	####	0.64	####	####	####	0.03
Q52LC2	V-type proton ATPase subunit S1-like protein OS=Homo sapiens GN=ATP6AP1L PE=2 SV=1 - [VAS1L_HUMAN]	1	-0.4	####	####	####	####	####	####	####	####	####	####	####	0.06	0.30	####	0.99	0.39	0.01
P02538	Keratin, type II cytoskeletal 6A OS=Homo sapiens GN=KRT6A PE=1 SV=3 - [K2C6A_HUMAN]	2	-0.4	####	####	0.25	####	####	####	0.36	0.01	####	####	0.39	0.15	####	####	####	####	0.03
O60840	Voltage-dependent L-type calcium channel subunit alpha-1F OS=Homo sapiens GN=CACNA1F PE=1 SV=2 - [CAC1F_HUMAN]	2	-0.4	0.70	####	####	0.61	0.11	####	####	0.02	####	####	####	0.70	####	####	####	####	0.04
O00461	Golgi integral membrane protein 4 OS=Homo sapiens GN=GOLIM4 PE=1 SV=1 - [GOLI4_HUMAN]	2	-0.4	####	####	####	####	####	####	####	####	####	####	####	####	####	####	####	####	0.00
P63267	Actin, gamma-enteric smooth muscle OS=Homo sapiens GN=ACTG2 PE=1 SV=1 - [ACTH_HUMAN]	1	-0.5	####	####	####	####	####	####	0.04	####	####	####	####	####	0.02	####	0.00	####	0.00
Q99729	Heterogeneous nuclear ribonucleoprotein A/B OS=Homo sapiens GN=HNRNPAB PE=1 SV=2 - [ROAA_HUMAN]	2	-0.5	0.11	####	####	####	0.14	####	####	####	0.51	####	####	0.03	####	####	####	####	0.00
Q9BUK6	Protein misato homolog 1 OS=Homo sapiens GN=MSTO1 PE=1 SV=1 - [MSTO1_HUMAN]	1	-0.5	####	####	####	0.16	####	####	####	0.47	####	####	####	####	0.26	0.15	####	0.97	0.03
Q9H4A9	Dipeptidase 2 OS=Homo sapiens GN=DPEP2 PE=1 SV=2 - [DPEP2_HUMAN]	1	-0.5	####	####	####	####	####	####	####	####	####	####	####	####	####	####	####	####	0.00
P41238	C->U-editing enzyme APOBEC-1 OS=Homo sapiens GN=APOBEC1 PE=1 SV=3 - [ABEC1_HUMAN]	1	-0.5	####	####	####	####	0.15	####	0.01	####	0.78	####	0.39	####	####	####	####	####	0.01
P83436	Conserved oligomeric Golgi complex subunit 7 OS=Homo sapiens GN=COG7 PE=1 SV=1 - [COG7_HUMAN]	1	-0.5	####	####	####	####	####	0.44	0.50	0.22	####	####	####	####	####	0.16	0.32	####	0.03
Q96PL2	Beta-tectorin OS=Homo sapiens GN=TECTB PE=2 SV=1 - [TECTB_HUMAN]	1	-0.5	0.50	0.14	0.68	0.72	####	####	####	0.04	####	####	####	####	####	####	####	####	0.03
P26447	Protein S100-A4 OS=Homo sapiens GN=S100A4 PE=1 SV=1 - [S10A4_HUMAN]	1	-0.5	####	####	0.92	####	####	####	####	####	####	####	0.49	####	####	####	0.68	####	0.04
Q9Y2D4	Exocyst complex component 6B OS=Homo sapiens GN=EXOC6B PE=1 SV=3 - [EXC6B_HUMAN]	1	-0.5	####	####	####	####	0.63	0.35	0.67	0.59	####	####	####	####	####	####	####	####	0.02
Q8TE85	Grainyhead-like protein 3 homolog OS=Homo sapiens GN=GRHL3 PE=1 SV=3 - [GRHL3_HUMAN]	1	-0.5	####	####	0.83	####	####	####	0.54	####	####	####	0.10	####	####	0.04	1.40	####	0.05
P48668	Keratin, type II cytoskeletal 6C OS=Homo sapiens GN=KRT6C PE=1 SV=3 - [K2C6C_HUMAN]	2	-0.5	####	0.30	0.66	0.30	####	0.02	0.32	0.01	####	####	####	####	####	####	####	####	0.02
Q86UQ4	ATP-binding cassette sub-family A member 13 OS=Homo sapiens GN=ABCA13 PE=2 SV=3 - [ABCAD_HUMAN]	3	-0.5	####	####	####	####	####	####	####	####	####	####	####	####	0.81	0.85	####	0.71	0.05
Q04756	Hepatocyte growth factor activator OS=Homo sapiens GN=HGFAC PE=1 SV=1 - [HGFA_HUMAN]	12	-0.5	####	####	####	####	####	0.23	####	####	####	####	####	####	####	####	####	####	0.00

P01772	Ig heavy chain V-III region KOL OS=Homo sapiens PE=1 SV=1 - [HV311_HUMAN]	1	-0.5	####	####	0.37	####	####	0.18	0.29	####	####	####	0.38	####	####	0.13	0.86	####	0.03
P01008	Antithrombin-III OS=Homo sapiens GN=SERPINC1 PE=1 SV=1 - [ANT3_HUMAN]	29	-0.5	0.18	####	####	####	0.26	####	####	####	0.07	####	####	####	0.30	####	####	####	0.01
Q8N3Y3	Glycosyltransferase-like protein LARGE2 OS=Homo sapiens GN=GYLTL1B PE=1 SV=2 - [LARG2_HUMAN]	1	-0.5	####	0.21	0.00	####	####	0.59	0.32	0.10	####	0.16	####	####	####	####	####	####	0.02
O14966	Ras-related protein Rab-7L1 OS=Homo sapiens GN=RAB29 PE=1 SV=1 - [RAB7L_HUMAN]	1	-0.5	0.28	####	1.08	0.81	####	####	0.32	0.09	####	####	####	####	####	####	####	####	0.04
Q14140	SERTA domain-containing protein 2 OS=Homo sapiens GN=SERTAD2 PE=1 SV=1 - [SRTD2_HUMAN]	1	-0.5	####	####	####	####	####	####	####	####	####	####	####	####	####	0.01	0.06	####	0.00
Q9Y4F5	Centrosomal protein of 170 kDa protein B OS=Homo sapiens GN=CEP170B PE=1 SV=4 - [C170B_HUMAN]	1	-0.5	####	####	####	####	####	####	####	####	####	####	####	####	0.64	0.59	0.96	0.19	0.02
O60248	Protein SOX-15 OS=Homo sapiens GN=SOX15 PE=1 SV=1 - [SOX15_HUMAN]	1	-0.5	####	####	####	0.29	####	####	####	0.76	####	####	####	0.63	####	####	####	0.67	0.04
P17858	ATP-dependent 6-phosphofructokinase, liver type OS=Homo sapiens GN=PFKL PE=1 SV=6 - [PFKAL_HUMAN]	2	-0.5	####	####	####	####	####	####	####	0.00	####	####	####	####	####	0.48	0.27	0.59	0.01
P42356	Phosphatidylinositol 4-kinase alpha OS=Homo sapiens GN=PI4KA PE=1 SV=3 - [PI4KA_HUMAN]	3	-0.5	####	####	1.22	0.86	####	####	####	####	####	####	0.06	####	####	####	####	####	0.02
P01860	Ig gamma-3 chain C region OS=Homo sapiens GN=IGHG3 PE=1 SV=2 - [IGHG3_HUMAN]	3	-0.5	####	####	####	####	####	####	####	####	####	####	####	####	####	####	####	####	0.00
P19827	Inter-alpha-trypsin inhibitor heavy chain H1 OS=Homo sapiens GN=ITIH1 PE=1 SV=3 - [ITIH1_HUMAN]	18	-0.5	####	####	####	####	####	####	####	####	####	####	####	####	####	####	####	0.07	0.00
Q9UNF1	Melanoma-associated antigen D2 OS=Homo sapiens GN=MAGED2 PE=1 SV=2 - [MAGD2_HUMAN]	2	-0.5	0.28	####	####	####	####	####	####	####	0.27	####	####	####	0.40	####	####	0.17	0.01
P04004	Vitronectin OS=Homo sapiens GN=VTN PE=1 SV=1 - [VTNC_HUMAN]	17	-0.5	####	####	####	####	####	####	####	####	####	0.50	####	0.05	####	0.44	####	####	0.01
Q9BZA0	Putative transcript Y 10 protein OS=Homo sapiens GN=TTTY10 PE=5 SV=1 - [TTY10_HUMAN]	1	-0.5	####	####	####	####	####	####	####	####	####	0.12	####	####	0.05	0.35	####	0.08	0.01
P08571	Monocyte differentiation antigen CD14 OS=Homo sapiens GN=CD14 PE=1 SV=2 - [CD14_HUMAN]	5	-0.5	0.11	####	####	####	0.83	0.03	####	####	0.09	####	####	####	0.48	####	####	####	0.01
Q16610	Extracellular matrix protein 1 OS=Homo sapiens GN=ECM1 PE=1 SV=2 - [ECM1_HUMAN]	7	-0.5	####	0.28	####	####	####	0.62	####	0.34	####	####	####	####	####	0.53	####	0.08	0.02
P80108	Phosphatidylinositol-glycan-specific phospholipase D OS=Homo sapiens GN=GPLD1 PE=1 SV=3 - [PHLD_HUMAN]	9	-0.5	####	####	####	####	####	####	####	####	0.05	####	####	0.59	0.00	####	####	0.47	0.03
P10909	Clusterin OS=Homo sapiens GN=CLU PE=1 SV=1 - [CLUS_HUMAN]	20	-0.5	####	####	####	####	####	####	####	####	####	####	####	####	####	####	####	0.15	0.00
Q02818	Nucleobindin-1 OS=Homo sapiens GN=NUCB1 PE=1 SV=4 - [NUCB1_HUMAN]	6	-0.5	####	####	####	####	####	####	####	0.10	####	####	####	####	0.28	0.45	####	1.12	0.01
O75155	Cullin-associated NEDD8-dissociated protein 2 OS=Homo sapiens GN=CAND2 PE=1 SV=3 - [CAND2_HUMAN]	2	-0.5	0.72	####	####	####	0.58	####	####	####	0.36	####	####	####	0.80	####	####	####	0.02
P06858	Lipoprotein lipase OS=Homo sapiens GN=LPL PE=1 SV=1 - [LIPL_HUMAN]	2	-0.5	####	####	0.86	####	####	####	0.11	####	####	####	0.22	####	####	####	0.81	####	0.01
Q8WVM7	Cohesin subunit SA-1 OS=Homo sapiens GN=STAG1 PE=1 SV=3 - [STAG1_HUMAN]	1	-0.5	####	####	####	0.60	####	####	####	0.14	####	####	####	####	####	0.29	####	0.90	0.02

Q03591	Complement factor H-related protein 1 OS=Homo sapiens GN=CFHR1 PE=1 SV=2 - [FHR1_HUMAN]	3	-0.5	0.28	0.29	####	####	####	####	####	####	####	####	####	####	####	####	####	####	0.00
Q9BZQ8	Protein Niban OS=Homo sapiens GN=FAM129A PE=1 SV=1 - [NIBAN_HUMAN]	1	-0.6	0.41	####	####	####	0.53	0.01	####	####	0.02	####	####	####	####	####	####	####	0.01
P00742	Coagulation factor X OS=Homo sapiens GN=F10 PE=1 SV=2 - [FA10_HUMAN]	16	-0.6	####	####	####	####	####	####	####	####	####	####	####	####	0.48	0.39	####	0.51	0.00
P48740	Mannan-binding lectin serine protease 1 OS=Homo sapiens GN=MASP1 PE=1 SV=3 - [MASP1_HUMAN]	3	-0.6	####	####	####	####	####	####	####	####	####	0.03	####	####	0.78	0.39	####	0.33	0.02
Q92954	Proteoglycan 4 OS=Homo sapiens GN=PRG4 PE=1 SV=2 - [PRG4_HUMAN]	17	-0.6	####	####	####	####	####	####	####	####	####	####	####	####	0.12	0.20	####	####	0.00
O75962	Triple functional domain protein OS=Homo sapiens GN=TRIO PE=1 SV=2 - [TRIO_HUMAN]	2	-0.6	####	####	####	####	####	####	####	####	####	####	####	####	0.54	0.22	0.81	0.44	0.01
P14543	Nidogen-1 OS=Homo sapiens GN=NID1 PE=1 SV=3 - [NID1_HUMAN]	1	-0.6	####	####	####	####	####	####	####	####	####	####	####	####	0.35	####	####	0.14	0.00
P35858	Insulin-like growth factor-binding protein complex acid labile subunit OS=Homo sapiens GN=IGFALS PE=1 SV=1 - [ALS_HUMAN]	12	-0.6	####	####	####	####	####	####	####	####	####	####	####	####	0.32	0.05	####	0.40	0.00
Q96IY4	Carboxypeptidase B2 OS=Homo sapiens GN=CPB2 PE=1 SV=2 - [CBPB2_HUMAN]	3	-0.6	0.28	####	####	####	0.28	####	####	####	0.49	####	####	0.13	0.59	####	####	0.11	0.03
Q8N823	Zinc finger protein 611 OS=Homo sapiens GN=ZNF611 PE=2 SV=2 - [ZN611_HUMAN]	1	-0.6	####	####	0.05	####	####	####	####	####	####	####	####	####	####	0.05	0.27	####	0.00
P10599	Thioredoxin OS=Homo sapiens GN=TXN PE=1 SV=3 - [THIO_HUMAN]	2	-0.6	0.97	0.39	####	0.23	####	####	####	####	####	####	####	####	0.71	0.14	####	####	0.03
P00751	Complement factor B OS=Homo sapiens GN=CFB PE=1 SV=2 - [CFAB_HUMAN]	26	-0.6	0.05	####	####	0.09	####	####	####	####	####	####	####	####	0.21	####	####	0.27	0.00
Q3MJ40	Coiled-coil domain-containing protein 144B OS=Homo sapiens GN=CCDC144B PE=2 SV=1 - [C144B_HUMAN]	2	-0.6	####	####	####	0.08	####	####	####	####	####	####	####	####	####	####	####	####	0.00
Q12805	EGF-containing fibulin-like extracellular matrix protein 1 OS=Homo sapiens GN=EFEMP1 PE=1 SV=2 - [FBLN3_HUMAN]	4	-0.6	####	####	####	####	####	0.03	####	####	####	0.15	####	####	0.21	0.59	####	####	0.00
P68871	Hemoglobin subunit beta OS=Homo sapiens GN=HBB PE=1 SV=2 - [HBB_HUMAN]	7	-0.6	####	####	0.65	####	####	####	####	####	####	####	1.21	0.14	####	####	0.68	####	0.04
Q9UF56	F-box/LRR-repeat protein 17 OS=Homo sapiens GN=FBXL17 PE=2 SV=3 - [FXL17_HUMAN]	1	-0.6	####	0.50	####	####	####	0.20	####	####	####	0.98	####	0.29	####	####	####	####	0.02
P18464	HLA class I histocompatibility antigen, B-51 alpha chain OS=Homo sapiens GN=HLA-B PE=1 SV=1 - [1B51_HUMAN]	2	-0.6	####	####	####	####	####	####	####	####	####	####	####	####	0.83	0.60	####	0.04	0.01
Q9Y5H9	Protocadherin alpha-2 OS=Homo sapiens GN=PCDHA2 PE=1 SV=1 - [PCDA2_HUMAN]	1	-0.6	####	####	0.58	1.17	####	####	0.09	0.73	####	####	####	0.00	####	####	####	0.40	0.05
Q8IXL6	Extracellular serine/threonine protein kinase FAM20C OS=Homo sapiens GN=FAM20C PE=1 SV=2 - [DMP4_HUMAN]	1	-0.6	####	####	####	####	####	####	####	####	####	####	####	####	0.07	0.44	####	0.49	0.00
Q68CZ6	HAUS augmin-like complex subunit 3 OS=Homo sapiens GN=HAUS3 PE=1 SV=1 - [HAUS3_HUMAN]	1	-0.6	0.03	####	####	####	####	####	####	####	1.80	0.19	####	0.30	0.31	####	####	####	0.03
P58166	Inhibin beta E chain OS=Homo sapiens GN=INHBE PE=1 SV=1 - [INHBE_HUMAN]	2	-0.6	####	####	####	####	####	####	####	####	####	####	####	####	####	####	0.13	0.14	0.00

P26927	Hepatocyte growth factor-like protein OS=Homo sapiens GN=MST1 PE=1 SV=2 - [HGFL_HUMAN]	9	-0.6	0.17	####	####	####	0.84	0.63	####	####	####	####	####	####	####	####	####	####	0.01
P02748	Complement component C9 OS=Homo sapiens GN=C9 PE=1 SV=2 - [CO9_HUMAN]	16	-0.6	####	####	####	####	####	####	####	####	####	####	####	####	0.23	0.05	####	####	0.00
P08779	Keratin, type I cytoskeletal 16 OS=Homo sapiens GN=KRT16 PE=1 SV=4 - [K1C16_HUMAN]	4	-0.6	####	0.20	0.28	####	####	####	0.01	####	####	0.22	0.29	####	####	####	####	####	0.01
P01602	Ig kappa chain V-I region HK102 (Fragment) OS=Homo sapiens GN=IGKV1-5 PE=4 SV=1 - [KV110_HUMAN]	1	-0.6	####	####	####	####	1.60	1.27	0.15	0.58	0.05	####	####	####	####	####	####	####	0.04
Q9Y345	Sodium- and chloride-dependent glycine transporter 2 OS=Homo sapiens GN=SLC6A5 PE=1 SV=3 - [SC6A5_HUMAN]	1	-0.6	####	####	0.36	####	####	####	0.32	####	####	####	####	####	####	####	####	####	0.00
A4FU01	Myotubularin-related protein 11 OS=Homo sapiens GN=MTMR11 PE=2 SV=2 - [MTMRB_HUMAN]	1	-0.6	####	####	0.22	####	####	####	####	####	####	####	####	####	####	####	0.12	####	0.00
P01011	Alpha-1-antichymotrypsin OS=Homo sapiens GN=SERPINA3 PE=1 SV=2 - [AACT_HUMAN]	15	-0.6	####	####	####	####	####	####	####	####	####	####	####	####	0.57	0.31	####	####	0.01
Q9Y272	Dexamethasone-induced Ras-related protein 1 OS=Homo sapiens GN=RASD1 PE=1 SV=1 - [RASD1_HUMAN]	1	-0.6	####	####	####	####	####	####	####	####	####	0.33	####	0.77	####	0.71	####	1.12	0.03
Q9NQX4	Unconventional myosin-Vc OS=Homo sapiens GN=MYO5C PE=1 SV=2 - [MYO5C_HUMAN]	2	-0.6	####	####	####	####	####	####	####	####	####	####	####	####	0.41	####	0.48	0.42	0.03
P01699	Ig lambda chain V-I region VOR OS=Homo sapiens PE=1 SV=1 - [LV101_HUMAN]	2	-0.6	####	####	0.21	0.28	####	####	0.18	0.36	####	####	0.12	0.15	####	####	####	####	0.01
Q7Z2Z1	Treslin OS=Homo sapiens GN=TICRR PE=1 SV=2 - [TICRR_HUMAN]	2	-0.6	0.35	####	####	####	0.16	####	####	####	1.20	####	0.16	####	####	####	####	####	0.01
Q8IWK6	Probable G-protein coupled receptor 125 OS=Homo sapiens GN=GPR125 PE=1 SV=2 - [GP125_HUMAN]	1	-0.7	0.28	####	####	####	0.71	####	####	####	0.16	####	####	####	####	####	####	####	0.00
Q96DM3	Uncharacterized protein C18orf8 OS=Homo sapiens GN=C18orf8 PE=2 SV=2 - [MIC1_HUMAN]	1	-0.7	####	####	####	####	1.00	0.11	0.59	####	####	####	####	####	####	####	####	####	0.01
O75976	Carboxypeptidase D OS=Homo sapiens GN=CPD PE=1 SV=2 - [CBPD_HUMAN]	1	-0.7	####	####	1.07	####	####	####	####	####	####	####	1.03	####	####	####	0.97	####	0.04
Q96AQ6	Pre-B-cell leukemia transcription factor-interacting protein 1 OS=Homo sapiens GN=PBXIP1 PE=1 SV=1 - [PBIP1_HUMAN]	2	-0.7	####	####	####	####	0.15	0.27	####	0.37	####	####	####	####	0.02	####	####	0.24	0.01
Q8WX92	Negative elongation factor B OS=Homo sapiens GN=NELFB PE=1 SV=1 - [NELFB_HUMAN]	1	-0.7	0.11	####	####	####	####	####	####	####	####	####	####	####	####	####	####	####	0.00
P07357	Complement component C8 alpha chain OS=Homo sapiens GN=C8A PE=1 SV=2 - [CO8A_HUMAN]	6	-0.7	####	####	####	####	####	####	####	####	####	####	####	####	####	####	####	####	0.00
Q5VT97	Rho GTPase-activating protein SYDE2 OS=Homo sapiens GN=SYDE2 PE=1 SV=2 - [SYDE2_HUMAN]	1	-0.7	####	####	####	0.46	####	####	####	0.38	####	####	####	0.05	####	####	####	1.29	0.01
P08603	Complement factor H OS=Homo sapiens GN=CFH PE=1 SV=4 - [CFAH_HUMAN]	32	-0.7	####	####	####	####	####	####	####	####	####	####	####	####	0.09	0.23	0.05	####	0.00
Q6ZTR5	Uncharacterized protein CXorf22 OS=Homo sapiens GN=CXorf22 PE=2 SV=3 - [CX022_HUMAN]	2	-0.7	0.09	####	####	####	0.86	####	####	0.04	####	####	####	####	0.83	####	####	0.00	0.01
Q9NQX3	Gephyrin OS=Homo sapiens GN=GPHN PE=1 SV=1 - [GEPH_HUMAN]	2	-0.7	####	####	####	####	####	####	####	####	####	####	####	####	####	0.43	####	0.42	0.01
P50461	Cysteine and glycine-rich protein 3 OS=Homo sapiens GN=CSRP3 PE=1 SV=1 - [CSRP3_HUMAN]	1	-0.7	####	####	####	####	####	0.12	####	0.51	####	####	####	####	####	####	####	0.36	0.02

Q9BPW4	Apolipoprotein L4 OS=Homo sapiens GN=APOL4 PE=2 SV=3 - [APOL4_HUMAN]	1	-0.7	####	####	####	####	####	####	####	####	####	####	####	####	0.07	####	####	####	0.00
Q96A19	Coiled-coil domain-containing protein 102A OS=Homo sapiens GN=CCDC102A PE=1 SV=2 - [C102A_HUMAN]	1	-0.7	####	####	####	####	####	####	####	####	####	####	####	####	0.37	0.74	0.77	0.42	0.01
O15195	Villin-like protein OS=Homo sapiens GN=VILL PE=2 SV=3 - [VILL_HUMAN]	1	-0.7	####	####	####	####	####	####	####	####	####	####	####	####	####	0.10	####	0.06	0.00
P04114	Apolipoprotein B-100 OS=Homo sapiens GN=APOB PE=1 SV=2 - [APOB_HUMAN]	125	-0.7	####	####	####	####	####	####	####	####	####	####	####	####	####	####	####	0.42	0.00
Q8N4Q0	Zinc-binding alcohol dehydrogenase domain-containing protein 2 OS=Homo sapiens GN=ZADH2 PE=1 SV=1 - [ZADH2_HUMAN]	1	-0.7	####	####	####	0.49	####	####	####	0.32	####	####	####	0.62	####	####	####	####	0.00
P06733	Alpha-enolase OS=Homo sapiens GN=ENO1 PE=1 SV=2 - [ENOA_HUMAN]	3	-0.7	####	####	####	####	####	####	####	####	####	####	####	####	0.03	####	####	0.18	0.00
Q7Z3T8	Zinc finger FYVE domain-containing protein 16 OS=Homo sapiens GN=ZFYVE16 PE=1 SV=3 - [ZFY16_HUMAN]	1	-0.7	0.06	####	####	0.28	####	####	####	####	####	####	####	0.18	0.25	####	####	0.46	0.01
Q13136	Liprin-alpha-1 OS=Homo sapiens GN=PPFIA1 PE=1 SV=1 - [LIPA1_HUMAN]	1	-0.7	####	####	####	####	####	####	####	####	####	####	####	0.29	####	####	####	####	0.00
P23468	Receptor-type tyrosine-protein phosphatase delta OS=Homo sapiens GN=PTPRD PE=1 SV=2 - [PTPRD_HUMAN]	1	-0.7	####	####	0.18	####	####	####	0.17	####	####	####	0.48	####	####	####	0.52	####	0.03
Q06033	Inter-alpha-trypsin inhibitor heavy chain H3 OS=Homo sapiens GN=ITIH3 PE=1 SV=2 - [ITIH3_HUMAN]	16	-0.7	####	####	####	####	####	####	####	####	####	####	####	####	####	0.00	####	0.05	0.00
P10643	Complement component C7 OS=Homo sapiens GN=C7 PE=1 SV=2 - [CO7_HUMAN]	7	-0.7	####	####	####	####	####	####	####	####	####	####	####	####	####	0.02	####	0.14	0.00
P27169	Serum paraoxonase/arylesterase 1 OS=Homo sapiens GN=PON1 PE=1 SV=3 - [PON1_HUMAN]	11	-0.7	####	####	####	####	####	####	####	####	0.36	0.23	####	0.47	0.36	0.09	####	0.26	0.03
Q9GZN2	Homeobox protein TGIF2 OS=Homo sapiens GN=TGIF2 PE=1 SV=1 - [TGIF2_HUMAN]	1	-0.7	####	####	####	####	####	####	####	####	####	0.23	0.05	0.49	####	####	####	####	0.00
P06396	Gelsolin OS=Homo sapiens GN=GSN PE=1 SV=1 - [GELS_HUMAN]	20	-0.7	####	####	####	####	####	####	####	####	####	####	####	####	####	####	####	0.28	0.00
P01815	Ig heavy chain V-II region COR OS=Homo sapiens PE=1 SV=1 - [HV202_HUMAN]	1	-0.7	####	####	####	####	####	####	####	0.01	####	####	####	0.64	####	####	####	####	0.00
Q14515	SPARC-like protein 1 OS=Homo sapiens GN=SPARCL1 PE=1 SV=2 - [SPRL1_HUMAN]	6	-0.8	####	####	####	####	####	####	####	####	####	####	####	####	####	####	####	####	0.00
Q68DK2	Zinc finger FYVE domain-containing protein 26 OS=Homo sapiens GN=ZFYVE26 PE=1 SV=3 - [ZFY26_HUMAN]	1	-0.8	0.08	####	####	####	0.11	####	####	####	####	####	####	####	####	####	####	####	0.00
Q5SVZ6	Zinc finger MYM-type protein 1 OS=Homo sapiens GN=ZMYM1 PE=2 SV=1 - [ZMYM1_HUMAN]	2	-0.8	####	####	####	0.74	####	####	####	0.06	####	####	####	####	0.14	####	####	0.99	0.01
P13671	Complement component C6 OS=Homo sapiens GN=C6 PE=1 SV=3 - [CO6_HUMAN]	11	-0.8	####	####	####	####	####	####	####	####	####	####	####	####	####	####	####	####	0.00
O00463	TNF receptor-associated factor 5 OS=Homo sapiens GN=TRAF5 PE=1 SV=2 - [TRAF5_HUMAN]	1	-0.8	0.22	####	####	####	0.25	####	####	####	0.26	####	####	####	0.80	0.40	####	####	0.03
P0CG05	Ig lambda-2 chain C regions OS=Homo sapiens GN=IGLC2 PE=1 SV=1 - [LAC2_HUMAN]	2	-0.8	####	####	0.60	0.03	####	####	0.27	####	####	####	0.20	####	####	####	0.26	0.00	0.03
P00736	Complement C1r subcomponent OS=Homo sapiens GN=C1R PE=1 SV=2 - [C1R_HUMAN]	9	-0.8	####	####	####	####	####	####	####	####	####	####	####	####	0.09	####	####	####	0.00

Q15431	Synaptonemal complex protein 1 OS=Homo sapiens GN=SYCP1 PE=1 SV=2 - [SYCP1_HUMAN]	1	-0.8	####	####	####	####	####	####	####	####	####	####	####	####	0.06	0.04	####	0.76	0.00
P22792	Carboxypeptidase N subunit 2 OS=Homo sapiens GN=CPN2 PE=1 SV=3 - [CPN2_HUMAN]	5	-0.8	####	####	####	####	####	####	####	####	####	####	####	####	####	0.51	####	0.56	0.00
Q9P2F5	Storkhead-box protein 2 OS=Homo sapiens GN=STOX2 PE=2 SV=2 - [STOX2_HUMAN]	2	-0.8	####	####	####	0.05	####	####	####	0.18	####	####	####	0.78	####	####	####	0.50	0.01
Q9ULI1	NACHT and WD repeat domain-containing protein 2 OS=Homo sapiens GN=NWD2 PE=2 SV=3 - [NWD2_HUMAN]	1	-0.8	####	0.17	####	####	0.02	0.42	0.32	0.29	####	####	####	####	####	####	####	####	0.01
Q5T9A4	ATPase family AAA domain-containing protein 3B OS=Homo sapiens GN=ATAD3B PE=1 SV=1 - [ATD3B_HUMAN]	1	-0.8	####	####	0.38	####	####	0.03	0.36	####	####	####	####	####	####	####	####	####	0.01
P06310	Ig kappa chain V-II region RPMI 6410 OS=Homo sapiens PE=4 SV=1 - [KV206_HUMAN]	2	-0.8	####	####	0.72	0.20	####	####	0.79	0.67	####	####	0.71	0.41	####	####	0.24	0.11	0.05
Q9GZV1	Ankyrin repeat domain-containing protein 2 OS=Homo sapiens GN=ANKRD2 PE=1 SV=3 - [ANKR2_HUMAN]	1	-0.8	0.22	####	####	####	0.36	####	####	####	####	####	####	####	0.07	####	####	####	0.00
P49908	Selenoprotein P OS=Homo sapiens GN=SEPP1 PE=1 SV=3 - [SEPP1_HUMAN]	9	-0.8	####	####	####	####	####	####	####	####	####	####	####	####	####	0.07	####	####	0.00
Q02040	A-kinase anchor protein 17A OS=Homo sapiens GN=AKAP17A PE=1 SV=2 - [AK17A_HUMAN]	1	-0.8	####	####	0.74	####	####	####	0.59	####	####	####	0.52	####	####	####	0.21	####	0.01
A0FGR9	Extended synaptotagmin-3 OS=Homo sapiens GN=ESYT3 PE=1 SV=1 - [ESYT3_HUMAN]	1	-0.8	####	####	####	####	0.98	0.46	0.14	0.34	####	####	####	####	####	####	####	####	0.00
Q9BYW2	Histone-lysine N-methyltransferase SETD2 OS=Homo sapiens GN=SETD2 PE=1 SV=3 - [SETD2_HUMAN]	1	-0.8	0.22	####	####	####	0.01	####	####	####	1.03	0.21	####	####	####	####	####	####	0.00
Q14520	Hyaluronan-binding protein 2 OS=Homo sapiens GN=HABP2 PE=1 SV=1 - [HABP2_HUMAN]	11	-0.8	####	####	####	####	####	0.07	####	####	0.73	0.66	####	####	####	####	####	####	0.02
Q96PD5	N-acetylmuramoyl-L-alanine amidase OS=Homo sapiens GN=PGLYRP2 PE=1 SV=1 - [PGRP2_HUMAN]	9	-0.8	####	####	####	####	####	####	####	0.07	####	####	####	####	####	####	####	####	0.00
P02675	Fibrinogen beta chain OS=Homo sapiens GN=FGB PE=1 SV=2 - [FIBB_HUMAN]	2	-0.8	####	####	####	0.52	####	####	####	0.53	####	####	####	####	####	####	####	0.04	0.01
Q9NZ52	ADP-ribosylation factor-binding protein GGA3 OS=Homo sapiens GN=GGA3 PE=1 SV=1 - [GGA3_HUMAN]	1	-0.8	####	####	####	####	####	####	####	####	####	0.01	####	####	####	####	####	####	0.00
P12724	Eosinophil cationic protein OS=Homo sapiens GN=RNASE3 PE=1 SV=2 - [ECP_HUMAN]	1	-0.8	####	####	####	####	####	####	####	####	####	####	####	####	####	####	0.30	0.05	0.00
Q9UIA9	Exportin-7 OS=Homo sapiens GN=XPO7 PE=1 SV=3 - [XPO7_HUMAN]	1	-0.8	####	####	####	####	####	####	####	0.52	####	####	####	0.08	####	####	####	1.00	0.00
Q96KN2	Beta-Ala-His dipeptidase OS=Homo sapiens GN=CNDP1 PE=1 SV=4 - [CNDP1_HUMAN]	10	-0.8	####	####	####	####	####	####	####	0.04	####	####	####	####	0.16	####	####	0.76	0.00
P15822	Zinc finger protein 40 OS=Homo sapiens GN=HIVEP1 PE=1 SV=3 - [ZEP1_HUMAN]	1	-0.8	####	####	####	####	####	####	####	0.31	####	####	####	0.26	####	####	####	0.24	0.01
Q86U17	Serpin A11 OS=Homo sapiens GN=SERPINA11 PE=2 SV=2 - [SPA11_HUMAN]	3	-0.8	####	####	####	####	####	####	####	####	####	####	####	####	####	####	####	0.00	0.00
Q9UII4	E3 ISG15--protein ligase HERC5 OS=Homo sapiens GN=HERC5 PE=1 SV=2 - [HERC5_HUMAN]	2	-0.8	####	####	####	0.55	####	####	####	####	1.16	0.95	0.79	0.77	####	####	####	####	0.05
P01861	Ig gamma-4 chain C region OS=Homo sapiens GN=IGHG4 PE=1 SV=1 - [IGHG4_HUMAN]	4	-0.8	####	####	####	####	####	####	####	####	####	####	####	####	####	####	####	####	0.00

P00450	Ceruloplasmin OS=Homo sapiens GN=CP PE=1 SV=1 - [CERU_HUMAN]	46	-0.9	####	####	####	####	####	####	####	####	0.37	####	####	0.17	0.32	####	####	0.30	0.01
Q6ZSR9	Uncharacterized protein FLJ45252 OS=Homo sapiens PE=1 SV=2 - [YJ005_HUMAN]	1	-0.9	0.18	####	0.14	####	0.62	####	0.54	0.33	####	####	####	####	0.17	####	0.17	####	0.04
A7E2Y1	Myosin-7B OS=Homo sapiens GN=MYH7B PE=1 SV=3 - [MYH7B_HUMAN]	3	-0.9	####	####	####	####	####	####	####	####	0.33	####	0.01	####	####	####	####	####	0.00
Q8TD84	Down syndrome cell adhesion molecule-like protein 1 OS=Homo sapiens GN=DSCAML1 PE=1 SV=2 - [DSCL1_HUMAN]	1	-0.9	####	0.42	####	####	####	####	####	####	####	####	####	####	####	####	####	####	0.00
Q96MH2	Protein HEXIM2 OS=Homo sapiens GN=HEXIM2 PE=1 SV=1 - [HEXI2_HUMAN]	1	-0.9	####	####	####	####	####	####	####	####	0.55	####	####	0.36	0.12	####	####	####	0.00
Q14571	Inositol 1,4,5-trisphosphate receptor type 2 OS=Homo sapiens GN=ITPR2 PE=1 SV=2 - [ITPR2_HUMAN]	1	-0.9	####	####	####	0.12	####	####	####	####	####	####	####	####	####	####	####	0.31	0.00
Q9NZD4	Alpha-hemoglobin-stabilizing protein OS=Homo sapiens GN=AHSP PE=1 SV=1 - [AHSP_HUMAN]	1	-0.9	####	####	0.97	####	####	####	0.18	####	####	####	0.47	####	####	####	0.38	####	0.00
P26639	Threonine--tRNA ligase, cytoplasmic OS=Homo sapiens GN=TARS PE=1 SV=3 - [SYTC_HUMAN]	1	-0.9	0.34	####	####	####	0.59	####	####	####	0.25	####	####	####	####	####	####	####	0.00
Q9Y4A0	Jerky protein homolog-like OS=Homo sapiens GN=JRKL PE=2 SV=2 - [JERKL_HUMAN]	1	-0.9	####	####	1.09	0.18	####	####	####	####	####	####	0.82	0.02	####	####	1.01	0.05	0.02
Q7KZ85	Transcription elongation factor SPT6 OS=Homo sapiens GN=SUPT6H PE=1 SV=2 - [SPT6H_HUMAN]	1	-0.9	####	####	####	####	####	####	####	####	####	####	####	####	####	####	0.03	####	0.00
O95996	Adenomatous polyposis coli protein 2 OS=Homo sapiens GN=APC2 PE=1 SV=1 - [APC2_HUMAN]	1	-0.9	####	####	####	####	####	####	####	####	####	####	####	####	####	####	####	####	0.00
O15078	Centrosomal protein of 290 kDa OS=Homo sapiens GN=CEP290 PE=1 SV=2 - [CE290_HUMAN]	5	-0.9	0.02	####	####	####	0.10	####	####	####	####	####	####	####	####	####	####	####	0.00
Q9Y262	Eukaryotic translation initiation factor 3 subunit L OS=Homo sapiens GN=EIF3L PE=1 SV=1 - [EIF3L_HUMAN]	1	-0.9	####	####	####	0.52	####	####	####	0.24	####	####	####	0.07	0.03	####	####	0.65	0.03
P43652	Afamin OS=Homo sapiens GN=AFM PE=1 SV=1 - [AFAM_HUMAN]	21	-1.0	####	####	####	####	####	####	####	####	####	####	####	####	####	0.09	####	####	0.01
Q9BV73	Centrosome-associated protein CEP250 OS=Homo sapiens GN=CEP250 PE=1 SV=2 - [CP250_HUMAN]	3	-1.0	####	####	####	####	####	####	####	####	####	####	####	####	2.14	2.16	0.80	####	0.02
Q5JWR5	Protein dopey-1 OS=Homo sapiens GN=DOPEY1 PE=2 SV=1 - [DOP1_HUMAN]	1	-1.0	####	####	####	0.00	####	####	0.16	0.24	####	####	####	####	####	####	0.31	0.29	0.02
Q76LX8	A disintegrin and metalloproteinase with thrombospondin motifs 13 OS=Homo sapiens GN=ADAMTS13 PE=1 SV=1 - [ATS13_HUMAN]	2	-1.0	####	####	####	####	####	####	####	####	####	####	####	####	0.19	0.68	####	0.39	0.00
Q9UKN7	Unconventional myosin-XV OS=Homo sapiens GN=MYO15A PE=1 SV=2 - [MYO15_HUMAN]	1	-1.0	####	####	####	####	1.67	1.63	0.03	0.57	####	0.75	####	####	####	####	####	####	0.02
P09848	Lactase-phlorizin hydrolase OS=Homo sapiens GN=LCT PE=1 SV=3 - [LPH_HUMAN]	1	-1.0	####	####	####	####	####	####	####	####	####	####	####	####	####	####	####	0.16	0.00
Q6UXB8	Peptidase inhibitor 16 OS=Homo sapiens GN=PI16 PE=1 SV=1 - [PI16_HUMAN]	3	-1.0	####	####	####	####	####	####	####	####	####	####	####	####	####	####	####	0.06	0.00
Q8NFY4	Semaphorin-6D OS=Homo sapiens GN=SEMA6D PE=2 SV=1 - [SEM6D_HUMAN]	1	-1.0	####	####	####	####	####	####	####	####	####	####	####	####	0.12	####	####	0.25	0.00

Q8NCX0	Coiled-coil domain-containing protein 150 OS=Homo sapiens GN=CCDC150 PE=2 SV=2 - [CC150_HUMAN]	1	-1.0	####	####	####	0.06	####	####	####	####	####	####	####	####	####	0.02	####	0.80	0.00
Q6DN90	IQ motif and SEC7 domain-containing protein 1 OS=Homo sapiens GN=IQSEC1 PE=1 SV=1 - [IQEC1_HUMAN]	1	-1.0	####	####	####	0.64	####	####	####	0.75	####	####	####	####	####	####	####	0.35	0.00
O14986	Phosphatidylinositol 4-phosphate 5-kinase type-1 beta OS=Homo sapiens GN=PIP5K1B PE=1 SV=2 - [PI51B_HUMAN]	1	-1.0	####	####	####	####	0.28	####	0.24	0.67	0.21	####	0.15	0.64	####	####	####	####	0.02
P09871	Complement C1s subcomponent OS=Homo sapiens GN=C1S PE=1 SV=1 - [C1S_HUMAN]	17	-1.0	####	####	####	####	####	####	####	####	####	####	####	####	0.07	0.13	####	0.10	0.00
Q8NFW1	Collagen alpha-1(XXII) chain OS=Homo sapiens GN=COL22A1 PE=1 SV=2 - [COMA1_HUMAN]	2	-1.0	####	####	####	0.02	####	####	####	####	####	####	####	####	####	####	####	0.70	0.00
P02751	Fibronectin OS=Homo sapiens GN=FN1 PE=1 SV=4 - [FINC_HUMAN]	33	-1.0	####	####	####	####	####	####	####	####	####	####	####	####	####	####	####	####	0.00
Q9HAR2	Latrophilin-3 OS=Homo sapiens GN=LPHN3 PE=1 SV=2 - [LPHN3_HUMAN]	1	-1.0	####	####	1.02	1.08	####	####	####	0.05	####	####	1.44	1.60	####	####	####	####	0.05
Q9UN37	Vacuolar protein sorting-associated protein 4A OS=Homo sapiens GN=VPS4A PE=1 SV=1 - [VPS4A_HUMAN]	1	-1.0	####	####	####	####	####	####	####	####	####	####	####	####	####	####	####	0.05	0.00
O95477	ATP-binding cassette sub-family A member 1 OS=Homo sapiens GN=ABCA1 PE=1 SV=3 - [ABCA1_HUMAN]	1	-1.0	####	####	####	0.31	####	####	####	0.29	####	####	0.33	0.16	####	####	####	####	0.02
Q96HR3	Mediator of RNA polymerase II transcription subunit 30 OS=Homo sapiens GN=MED30 PE=1 SV=1 - [MED30_HUMAN]	1	-1.0	####	####	####	####	####	####	####	####	####	####	####	####	####	####	####	####	0.00
Q96LM5	Uncharacterized protein C4orf45 OS=Homo sapiens GN=C4orf45 PE=2 SV=3 - [CD045_HUMAN]	1	-1.0	0.27	####	####	####	0.62	####	####	0.23	0.14	####	####	####	####	####	####	####	0.00
O00337	Sodium/nucleoside cotransporter 1 OS=Homo sapiens GN=SLC28A1 PE=1 SV=2 - [S28A1_HUMAN]	1	-1.0	####	####	####	0.33	####	####	####	0.56	####	####	####	####	####	####	####	0.54	0.00
Q8NFJ8	Class E basic helix-loop-helix protein 22 OS=Homo sapiens GN=BHLHE22 PE=2 SV=1 - [BHE22_HUMAN]	2	-1.0	####	####	####	####	####	####	####	####	0.48	0.61	####	####	####	####	####	####	0.00
Q6NUI6	Chondroadherin-like protein OS=Homo sapiens GN=CHADL PE=2 SV=2 - [CHADL_HUMAN]	1	-1.1	####	####	0.36	####	####	####	####	####	####	####	0.96	####	####	####	0.74	####	0.00
Q4ZG55	Protein GREB1 OS=Homo sapiens GN=GREB1 PE=2 SV=1 - [GREB1_HUMAN]	1	-1.1	####	####	####	0.20	####	####	####	####	####	####	####	####	####	####	####	0.76	0.00
Q96QH2	PML-RARA-regulated adapter molecule 1 OS=Homo sapiens GN=PRAM1 PE=1 SV=2 - [PRAM_HUMAN]	1	-1.1	####	####	####	####	####	####	####	####	####	####	####	0.07	####	####	####	####	0.00
Q5VYS8	Terminal uridylyltransferase 7 OS=Homo sapiens GN=ZCCHC6 PE=1 SV=1 - [TUT7_HUMAN]	1	-1.1	####	####	####	####	####	####	####	0.08	####	####	####	0.20	####	####	####	####	0.00
Q9Y3R0	Glutamate receptor-interacting protein 1 OS=Homo sapiens GN=GRIP1 PE=1 SV=3 - [GRIP1_HUMAN]	1	-1.1	####	####	####	####	0.04	0.61	####	####	####	0.07	####	####	####	0.28	####	####	0.00
Q9P2J5	Leucine--tRNA ligase, cytoplasmic OS=Homo sapiens GN=LARS PE=1 SV=2 - [SYLC_HUMAN]	1	-1.1	####	####	####	####	####	####	####	####	####	####	####	####	####	####	####	####	0.00
P20848	Putative alpha-1-antitrypsin-related protein OS=Homo sapiens GN=SERPINA2 PE=1 SV=1 - [A1ATR_HUMAN]	1	-1.1	####	####	0.31	####	####	####	####	####	####	####	####	####	0.07	####	0.97	####	0.00

P04196	Histidine-rich glycoprotein OS=Homo sapiens GN=HRG PE=1 SV=1 - [HRG_HUMAN]	19	-1.1	####	####	####	####	####	####	####	####	####	####	####	####	####	####	####	####	0.00
P18509	Pituitary adenylate cyclase-activating polypeptide OS=Homo sapiens GN=ADCYAP1 PE=1 SV=3 - [PACA_HUMAN]	2	-1.1	0.53	####	####	0.10	0.32	####	####	0.24	0.25	####	####	####	####	####	####	####	0.00
Q14C87	Transmembrane protein 132D OS=Homo sapiens GN=TMEM132D PE=1 SV=1 - [T132D_HUMAN]	1	-1.1	####	####	####	####	####	####	####	0.53	####	####	####	####	####	####	####	0.20	0.00
Q9NZP8	Complement C1r subcomponent-like protein OS=Homo sapiens GN=C1RL PE=1 SV=2 - [C1RL_HUMAN]	5	-1.1	####	####	####	0.01	####	####	####	0.27	####	####	####	####	####	####	####	0.43	0.00
Q9UK55	Protein Z-dependent protease inhibitor OS=Homo sapiens GN=SERPINA10 PE=1 SV=1 - [ZPI_HUMAN]	13	-1.1	####	####	####	####	####	####	####	####	####	####	####	####	0.32	####	####	0.30	0.00
P46736	Lys-63-specific deubiquitinase BRCC36 OS=Homo sapiens GN=BRCC3 PE=1 SV=2 - [BRCC3_HUMAN]	1	-1.1	####	####	####	####	0.32	####	0.08	0.77	####	####	####	0.42	####	####	####	####	0.00
Q9H4S2	GS homeobox 1 OS=Homo sapiens GN=GSX1 PE=2 SV=1 - [GSX1_HUMAN]	1	-1.1	####	####	####	0.68	####	####	####	####	####	####	####	####	####	####	####	0.46	0.00
Q9H1D0	Transient receptor potential cation channel subfamily V member 6 OS=Homo sapiens GN=TRPV6 PE=1 SV=2 - [TRPV6_HUMAN]	1	-1.1	####	####	0.36	0.43	####	####	####	0.08	####	####	0.47	0.64	####	####	0.36	0.37	0.05
P00748	Coagulation factor XII OS=Homo sapiens GN=F12 PE=1 SV=3 - [FA12_HUMAN]	13	-1.1	####	####	####	####	####	####	####	####	####	####	####	####	0.02	####	####	####	0.00
A6NJ08	Putative methyl-CpG-binding domain protein 3-like 5 OS=Homo sapiens GN=MBD3L5 PE=5 SV=1 - [MB3L5_HUMAN]	1	-1.1	####	####	####	####	0.66	####	####	####	1.12	0.06	####	0.68	####	####	####	####	0.01
O14981	TATA-binding protein-associated factor 172 OS=Homo sapiens GN=BTAF1 PE=1 SV=2 - [BTAF1_HUMAN]	1	-1.1	####	####	####	####	####	####	####	####	####	####	####	####	####	####	####	####	0.00
Q9NP73	Putative bifunctional UDP-N-acetylglucosamine transferase and deubiquitinase ALG13 OS=Homo sapiens GN=ALG13 PE=1 SV=2 - [ALG13_HUMAN]	1	-1.1	0.68	####	####	####	0.35	####	####	####	0.67	####	####	####	0.22	####	####	####	0.00
Q5W0Q7	SUMO-specific isopeptidase USPL1 OS=Homo sapiens GN=USPL1 PE=1 SV=1 - [USPL1_HUMAN]	2	-1.2	####	####	####	####	0.43	####	####	0.12	####	####	####	####	####	####	####	####	0.00
B3KS81	Serine/arginine repetitive matrix protein 5 OS=Homo sapiens GN=SRRM5 PE=1 SV=3 - [SRRM5_HUMAN]	1	-1.2	####	####	####	####	####	####	####	####	####	####	####	####	0.26	0.28	####	####	0.00
P46379	Large proline-rich protein BAG6 OS=Homo sapiens GN=BAG6 PE=1 SV=2 - [BAG6_HUMAN]	1	-1.2	####	####	####	0.12	####	####	####	####	####	####	####	####	####	####	####	####	0.00
Q7Z5N4	Protein sidekick-1 OS=Homo sapiens GN=SDK1 PE=1 SV=3 - [SDK1_HUMAN]	1	-1.2	####	####	####	####	0.76	####	####	0.45	####	####	####	####	####	####	####	####	0.00
A6NDK9	Putative golgin subfamily A member 6C OS=Homo sapiens GN=GOLGA6C PE=5 SV=1 - [GOG6C_HUMAN]	1	-1.2	####	####	####	####	####	####	####	####	####	0.29	####	0.39	####	####	####	####	0.00
P29459	Interleukin-12 subunit alpha OS=Homo sapiens GN=IL12A PE=1 SV=2 - [IL12A_HUMAN]	1	-1.2	####	####	####	####	####	####	####	0.07	####	####	####	####	####	####	####	####	0.00
Q96K62	Zinc finger and BTB domain-containing protein 45 OS=Homo sapiens GN=ZBTB45 PE=2 SV=1 - [ZBT45_HUMAN]	1	-1.2	####	####	####	####	####	####	0.62	0.05	####	####	0.45	####	####	####	####	####	0.00

Q3V6T2	Girdin OS=Homo sapiens GN=CCDC88A PE=1 SV=2 - [GRDN_HUMAN]	2	-1.2	####	####	####	####	####	####	####	####	####	####	####	####	####	####	####	0.29	0.00
Q8IVH8	Mitogen-activated protein kinase kinase kinase kinase 3 OS=Homo sapiens GN=MAP4K3 PE=1 SV=1 - [M4K3_HUMAN]	1	-1.2	####	####	####	0.54	####	####	####	0.33	####	####	####	####	####	####	####	0.47	0.00
Q96KS0	Egl nine homolog 2 OS=Homo sapiens GN=EGLN2 PE=1 SV=1 - [EGLN2_HUMAN]	1	-1.2	####	####	####	####	####	####	####	####	####	####	####	####	####	####	####	####	0.00
P15169	Carboxypeptidase N catalytic chain OS=Homo sapiens GN=CPN1 PE=1 SV=1 - [CBPN_HUMAN]	12	-1.2	####	####	####	####	####	####	####	####	####	####	####	0.03	####	####	####	0.07	0.00
P00338	L-lactate dehydrogenase A chain OS=Homo sapiens GN=LDHA PE=1 SV=2 - [LDHA_HUMAN]	1	-1.2	####	####	####	####	####	####	####	####	####	####	####	####	####	####	####	####	0.00
Q92839	Hyaluronan synthase 1 OS=Homo sapiens GN=HAS1 PE=1 SV=2 - [HYAS1_HUMAN]	1	-1.2	####	####	####	####	0.74	####	####	0.21	####	####	####	####	0.01	####	####	####	0.00
Q9NST1	Patatin-like phospholipase domain-containing protein 3 OS=Homo sapiens GN=PNPLA3 PE=1 SV=2 - [PLPL3_HUMAN]	1	-1.3	####	####	####	####	####	####	####	####	####	####	####	####	####	####	####	####	0.00
Q9UHG3	Prenylcysteine oxidase 1 OS=Homo sapiens GN=PCYOX1 PE=1 SV=3 - [PCYOX_HUMAN]	1	-1.3	####	####	####	0.49	####	####	####	0.18	####	####	####	####	####	####	####	0.70	0.00
Q8WU90	Zinc finger CCCH domain-containing protein 15 OS=Homo sapiens GN=ZC3H15 PE=1 SV=1 - [ZC3HF_HUMAN]	1	-1.3	####	####	####	####	####	####	####	####	####	####	####	####	####	####	####	####	0.00
P19823	Inter-alpha-trypsin inhibitor heavy chain H2 OS=Homo sapiens GN=ITIH2 PE=1 SV=2 - [ITIH2_HUMAN]	46	-1.3	####	####	####	####	####	####	####	####	####	####	####	####	####	####	####	####	0.00
Q86SQ0	Pleckstrin homology-like domain family B member 2 OS=Homo sapiens GN=PHLDB2 PE=1 SV=2 - [PHLB2_HUMAN]	1	-1.3	####	####	####	####	####	####	####	####	####	####	####	####	####	####	####	####	0.00
O75094	Slit homolog 3 protein OS=Homo sapiens GN=SLIT3 PE=2 SV=3 - [SLIT3_HUMAN]	1	-1.3	####	####	####	####	0.48	####	####	####	####	####	####	####	0.66	0.16	0.11	####	0.01
Q6NSI8	Uncharacterized protein KIAA1841 OS=Homo sapiens GN=KIAA1841 PE=2 SV=2 - [K1841_HUMAN]	1	-1.3	####	####	####	####	####	####	####	####	####	####	####	####	####	0.03	####	####	0.00
P02144	Myoglobin OS=Homo sapiens GN=MB PE=1 SV=2 - [MYG_HUMAN]	2	-1.4	####	####	####	####	####	####	####	0.06	####	####	####	####	####	####	####	####	0.00
P55058	Phospholipid transfer protein OS=Homo sapiens GN=PLTP PE=1 SV=1 - [PLTP_HUMAN]	7	-1.4	####	####	####	####	####	####	####	####	####	####	####	####	0.16	####	####	####	0.00
Q9NQ79	Cartilage acidic protein 1 OS=Homo sapiens GN=CRTAC1 PE=1 SV=2 - [CRAC1_HUMAN]	2	-1.4	####	####	####	####	####	####	####	####	####	####	####	####	####	####	####	####	0.00
Q15154	Pericentriolar material 1 protein OS=Homo sapiens GN=PCM1 PE=1 SV=4 - [PCM1_HUMAN]	3	-1.4	####	####	####	####	####	####	####	####	####	0.35	0.44	0.31	####	0.15	####	0.85	0.00
Q9UG56	Phosphatidylserine decarboxylase proenzyme OS=Homo sapiens GN=PISD PE=2 SV=4 - [PISD_HUMAN]	1	-1.4	####	0.41	####	####	####	0.55	####	####	####	1.28	####	####	####	1.04	####	####	0.02
Q8IYD9	Lung adenoma susceptibility protein 2 OS=Homo sapiens GN=LAS2 PE=2 SV=1 - [LAS2_HUMAN]	1	-1.4	####	####	####	####	####	####	####	####	####	####	####	####	####	####	####	####	0.00
Q9NU22	Midasin OS=Homo sapiens GN=MDN1 PE=1 SV=2 - [MDN1_HUMAN]	2	-1.4	####	####	####	####	####	####	####	0.26	####	####	####	####	####	####	####	####	0.00
Q9HDC9	Adipocyte plasma membrane-associated protein OS=Homo sapiens GN=APMAP PE=1 SV=2 - [APMAP_HUMAN]	7	-1.5	####	####	####	####	####	####	####	####	####	####	####	####	####	####	####	0.81	0.00

P26006	Integrin alpha-3 OS=Homo sapiens GN=ITGA3 PE=1 SV=5 - [ITA3_HUMAN]	1	-1.5	####	####	####	####	####	1.10	####	####	####	####	####	####	####	####	####	####	0.00
P01009	Alpha-1-antitrypsin OS=Homo sapiens GN=SERPINA1 PE=1 SV=3 - [A1AT_HUMAN]	41	-1.5	####	####	####	####	####	####	####	####	####	####	####	####	0.28	####	####	####	0.00
P23470	Receptor-type tyrosine-protein phosphatase gamma OS=Homo sapiens GN=PTPRG PE=1 SV=4 - [PTPRG_HUMAN]	1	-1.5	####	####	####	####	####	####	####	####	####	####	####	####	####	####	####	####	0.00
Q53GL0	Pleckstrin homology domain-containing family O member 1 OS=Homo sapiens GN=PLEKHO1 PE=1 SV=2 - [PKHO1_HUMAN]	1	-1.5	####	####	####	####	####	####	####	0.18	####	####	####	####	####	####	####	####	0.00
Q8NBU5	ATPase family AAA domain-containing protein 1 OS=Homo sapiens GN=ATAD1 PE=1 SV=1 - [ATAD1_HUMAN]	1	-1.5	####	####	####	0.11	####	####	####	####	####	####	####	####	####	####	####	####	0.00
Q8N5R6	Coiled-coil domain-containing protein 33 OS=Homo sapiens GN=CCDC33 PE=1 SV=3 - [CCD33_HUMAN]	1	-1.5	####	####	####	####	####	####	####	####	####	####	####	####	0.96	0.03	####	####	0.00
P01019	Angiotensinogen OS=Homo sapiens GN=AGT PE=1 SV=1 - [ANGT_HUMAN]	13	-1.6	####	####	####	####	####	####	####	####	0.04	####	####	####	####	####	####	####	0.00
Q13576	Ras GTPase-activating-like protein IQGAP2 OS=Homo sapiens GN=IQGAP2 PE=1 SV=4 - [IQGA2_HUMAN]	1	-1.6	####	####	####	####	####	####	####	####	####	####	####	####	####	####	####	####	0.00
Q9BY12	S phase cyclin A-associated protein in the endoplasmic reticulum OS=Homo sapiens GN=SCAPER PE=1 SV=2 - [SCAPE_HUMAN]	2	-1.6	####	####	####	0.30	####	####	####	0.34	####	####	####	0.12	####	####	####	0.40	0.00
Q14573	Inositol 1,4,5-trisphosphate receptor type 3 OS=Homo sapiens GN=ITPR3 PE=1 SV=2 - [ITPR3_HUMAN]	1	-1.6	####	####	####	####	0.83	####	####	####	####	####	####	####	####	####	####	####	0.00
O14974	Protein phosphatase 1 regulatory subunit 12A OS=Homo sapiens GN=PPP1R12A PE=1 SV=1 - [MYPT1_HUMAN]	2	-1.6	####	####	####	0.02	####	####	####	####	####	####	####	####	####	####	####	####	0.00
A8MVX0	Rho guanine nucleotide exchange factor 33 OS=Homo sapiens GN=ARHGEF33 PE=2 SV=2 - [ARG33_HUMAN]	2	-1.7	####	####	####	0.94	####	####	####	0.57	####	####	####	1.20	####	####	####	####	0.00
P01031	Complement C5 OS=Homo sapiens GN=C5 PE=1 SV=4 - [CO5_HUMAN]	44	-1.7	####	####	####	####	####	####	####	####	####	####	####	####	####	####	####	####	0.00
O95831	Apoptosis-inducing factor 1, mitochondrial OS=Homo sapiens GN=AIFM1 PE=1 SV=1 - [AIFM1_HUMAN]	1	-1.7	####	####	0.05	0.84	####	####	####	####	####	####	####	0.22	####	####	####	####	0.00
O94967	WD repeat-containing protein 47 OS=Homo sapiens GN=WDR47 PE=1 SV=1 - [WDR47_HUMAN]	2	-1.8	####	####	####	2.31	####	####	####	1.23	####	####	####	####	####	####	####	0.17	0.00
Q9UKA4	A-kinase anchor protein 11 OS=Homo sapiens GN=AKAP11 PE=1 SV=1 - [AKA11_HUMAN]	1	-1.8	####	####	####	####	####	####	####	####	####	####	####	####	####	####	####	####	0.00
Q7Z2Y8	Interferon-induced very large GTPase 1 OS=Homo sapiens GN=GVINP1 PE=2 SV=2 - [GVIN1_HUMAN]	2	-1.9	####	####	####	####	####	####	####	####	####	####	####	####	####	####	####	####	0.00
P0C7X3	Putative cyclin-Y-like protein 3 OS=Homo sapiens GN=CCNYL3 PE=2 SV=1 - [CCYL3_HUMAN]	1	-1.9	####	####	####	####	####	####	####	0.40	####	####	####	####	####	####	####	####	0.00
P61647	Alpha-2,8-sialyltransferase 8F OS=Homo sapiens GN=ST8SIA6 PE=2 SV=1 - [SIA8F_HUMAN]	1	-2.0	####	####	####	0.26	####	####	####	####	####	####	####	0.01	####	####	####	0.42	0.00
Q569H4	Protein Largen OS=Homo sapiens GN=PRR16 PE=2 SV=1 - [LARGN_HUMAN]	1	-2.0	0.44	####	####	####	0.68	####	####	####	0.14	####	####	####	####	####	####	####	0.00

Q9BYJ1	Hydroperoxide isomerase ALOXE3 OS=Homo sapiens GN=ALOXE3 PE=1 SV=1 - [LOXE3_HUMAN]	1	-2.2	####	####	####	####	####	####	####	####	####	####	####	####	####	####	####	####	0.00
O60344	Endothelin-converting enzyme 2 OS=Homo sapiens GN=ECE2 PE=1 SV=4 - [ECE2_HUMAN]	1	-2.3	0.44	####	####	####	0.61	####	####	####	1.08	####	####	####	####	####	####	####	0.00
Q96EP5	DAZ-associated protein 1 OS=Homo sapiens GN=DAZAP1 PE=1 SV=1 - [DAZP1_HUMAN]	1	-2.4	####	####	####	####	####	####	####	####	####	####	####	####	####	####	####	####	0.00
Q16799	Reticulon-1 OS=Homo sapiens GN=RTN1 PE=1 SV=1 - [RTN1_HUMAN]	1	-2.5	####	####	####	####	####	####	####	####	0.59	####	####	####	####	####	####	####	0.00
